# Supplementary material for: Knowledge, attitudes, practices (KAP) and control of rabies among community households and health practitioners at the human-wildlife interface in Limpopo National Park, Massingir District, Mozambique
Source: PLoS Negl Trop Dis. 2022 Mar 7;16(3):e0010202. doi: 10.1371/journal.pntd.0010202 (PMC8929695; doi:10.1371/journal.pntd.0010202)
Supplement: S1 Table — (DOCX) [file pntd.0010202.s001.docx]

**S1 Table. Healthcare practices after dog bite in households in the preceding 12 months.**

| Healthcare practices after dog bite | Frequency (%) | 95% CI | *P* value |
| --- | --- | --- | --- |
| Source of biting dog in previous 12 months |  |  |  |
| Neighbours’ dogs | 27 (65.9) | 49.4-79.9 | .106 |
| Household’s dogs | 13 (31.7) | 18.1-48.1 |  |
| Unidentified strange dog | 1 (2.4) | 0.0-12.9 |  |
| Treatment applied after bite |  |  |  |
| Local bite area treatment | 28 (68.3) | 51.9-81.9 | 1.000 |
| Rabies PEP | 5 (12.2) | 4.1-26.2 |  |
| Traditional treatment | 4 (9.8) | 2.7-23.1 |  |
| Medical wound treatment | 3 (7.3) | 1.5-19.9 |  |
| Nothing | 1 (2.4) | 0.0-.12.9 |  |
| Not known | 1 (2.4) | 0.0-12.9 |  |
| Person in charge of treatment |  |  |  |
| Nurse | 23 (56.1) |  | .429 |
| Physician | 7 (17.1) |  |  |
| Traditional healer | 4 (9.8) |  |  |
| Family member | 3 (7.3) |  |  |
| Self | 2 (4.9) |  |  |
| Community member | 1 (2.4) |  |  |
| Dog’s status after biting |  |  |  |
| Healthy (alive) | 24 (58.5) |  | .861 |
| Died | 13 (31.7) |  |  |
| Don’t know | 4 (9.8) |  |  |
