# Supplementary material for: Knowledge, attitudes, practices (KAP) and control of rabies among community households and health practitioners at the human-wildlife interface in Limpopo National Park, Massingir District, Mozambique
Source: PLoS Negl Trop Dis. 2022 Mar 7;16(3):e0010202. doi: 10.1371/journal.pntd.0010202 (PMC8929695; doi:10.1371/journal.pntd.0010202)
Supplement: S2 Table — (DOCX) [file pntd.0010202.s002.docx]

**S2. Table. Frequency of dog vaccination by village and reasons for non-vaccinating dogs against rabies**

| Dog rabies vaccination and reasons for non-vaccinating | Frequency (%) | | | CI (95%) | *P* value |
| --- | --- | --- | --- | --- | --- |
| Village | Unvaccinated | Vaccinated | No dogs |  |  |
| Mavoze | 27 (11.6) | 14 (6.0) | 2 (0.9) |  |  |
| Macaringue | 27 (11.6) | 7 (3.0) | 7 (3.0) |  |  |
| Bingo | 26 (11.2) | 3 (1.3) | 0 (0.0) |  |  |
| Machamba | 18 (7.7) | 0 (0.0) | 5 (2.1) |  |  |
| Cunze | 16 (6.9) | 7 (3.0) | 2 (0.9) |  |  |
| Munhamane | 13 (5.6) | 12 (5.2) | 4 (1.7) |  |  |
| Madingane | 12 (5.2) | 13 (5.6) | 1 (0.4) |  |  |
| Mahlaúle | 9 (3.9) | 4 (1.7) | 4 (1.7) |  |  |
| Reasons for not having the dog vaccinated |  |  |  |  |  |
| Never had information on any vaccination campaign | 105 (62.1) | - | - | 54.3-69.5 | 1.000 |
| Absent at time of vaccination campaign | 17 (10.1) | - | - | 6.0-15.6 |  |
| Not aware of need to vaccinate | 16 (9.5) | - | - | 5.5-14.9 |  |
| Was still a puppy | 5 (3.0) | - | - | 1.0-6.8 |  |
| Was aggressive | 3 (1.8) | - | - | 0.4-5.1 |  |
| Don’t know where to get the vaccine | 2 (1.2) | - | - | 0.1-4.2 |  |
| Lack of time | 1 (0.6) | - | - | 0.0-3.3 |  |
