# Supplementary material for: Knowledge, attitudes, practices (KAP) and control of rabies among community households and health practitioners at the human-wildlife interface in Limpopo National Park, Massingir District, Mozambique
Source: PLoS Negl Trop Dis. 2022 Mar 7;16(3):e0010202. doi: 10.1371/journal.pntd.0010202 (PMC8929695; doi:10.1371/journal.pntd.0010202)
Supplement: S3 Table — (DOCX) [file pntd.0010202.s003.docx]

**S3. Table Knowledge of rabies causative agent, main animal source of rabies in Mozambique, mode of transmissions, clinical features, incubation and concept of rabies categories of exposure among Health practitioners, Massingir district**

| Variables | Frequency (%) |
| --- | --- |
| Causative agent of rabies |  |
| Virus | 32 (76.2) |
| Bacteria | 3 (7.1) |
| Don’t know | 7 (16.7) |
| Most important source of rabies in Mozambique |  |
| Dog | 36 (85.7) |
| Cat | 2 (4.8) |
| Wild animals | 2 (4.8) |
| Monkeys | 1 (2.4) |
| Other animal species | 4 (9.5) |
| Don’t know | 3 (7.1) |
| Mode of rabies transmission to humans |  |
| Bites | 37 (88.1) |
| Scratches by claws (nails) | 11 (26.2) |
| Contact with infected saliva | 11 (26.2) |
| Eating raw meat | 1 (2.4) |
| Blood contact | 1 (2.4) |
| Don’t know | 1 (2.4) |
| Incubation period of rabies virus in humans |  |
| < 1Week | 16 (38.1) |
| 1-5 Weeks | 7 (16.7) |
| < 3 Months | 1 (2.4) |
| Don’t know | 18 (42.9) |
| Clinical signs of rabies in humans |  |
| Death | 35 (83.3) |
| Hypersalivation | 28 (66.7) |
| Fever | 27 (64.3) |
| Hydrophobia | 21 (50.0) |
| Headache | 19 (45.2) |
| Hallucinations | 18 (42.9) |
| Anxiety | 16 (38.1) |
| Restlessness | 14 (33.3) |
| Nausea | 8 (19.0) |
| Insomnia | 7 (16.7) |
| Paralysis | 6 (14.3) |
| Dysphagia | 5 (11.9) |
| Don’t know | 2 (4.8) |
| Knowledge on the association between severity/location of the bite and onset of clinical signs |  |
| Yes | 7 (16.7) |
| No | 24 (57.1) |
| Don't know/Declined to answer | 9 (21.4) |
| Unsure | 2 (4.8) |
| Concept of rabies categories of exposure |  |
| Yes | 29 (69.1) |
| No | 10 (23.8) |
| Don't know/Declined to answer | 3 (7.1) |
