# Supplementary material for: Knowledge, attitudes, practices (KAP) and control of rabies among community households and health practitioners at the human-wildlife interface in Limpopo National Park, Massingir District, Mozambique
Source: PLoS Negl Trop Dis. 2022 Mar 7;16(3):e0010202. doi: 10.1371/journal.pntd.0010202 (PMC8929695; doi:10.1371/journal.pntd.0010202)
Supplement: Attachment — Submitted filename: PNTD-D-20-01733_R2_reviewer GM.pdf [file pntd.0010202.s008.pdf]

PLOS Neglected Tropical Diseases

Knowledge, attitudes, practices (KAPs) and control of rabies among community households and health practitioners at the human-wildlife interface in Limpopo National Park, Massingir District, Mozambique

--Manuscript Draft--

**Manuscript Number:**	PNTD-D-20-01733R2
**Full Title:**	Knowledge, attitudes, practices (KAPs) and control of rabies among community households and health practitioners at the human-wildlife interface in Limpopo National Park, Massingir District, Mozambique
**Short Title:**	Knowledge, attitudes, practices (KAPs) and control of rabies in Limpopo National Park
**Article Type:**	Research Article
**Keywords:**	awareness; dog; bite; rabies; households; health practitioners; Limpopo National Park; Massingir; Mozambique
**Abstract:**	Background<br>Rabies is a deadly viral and zoonotic disease that kills more than 26,000 people each year in Africa. In Mozambique, poverty and inadequate surveillance result in gross underreporting and ineffective control of the disease in animals and people. Little is known of the role of human attitudes and behaviour in prevention or control of rabies, thus this study was undertaken to assess the knowledge, attitudes and practices amongst selected households and health practitioners in one affected area, the Limpopo National Park (LNP), Massingir district.<br><br>Methodology<br>A cross-sectional study was conducted of 233 households in eight villages in LNP and among 42 health practitioners from eight health facilities in Massingir between 2016 and 2018. Permission for the study was first obtained from village leaders before consenting household representatives aged 18 years or over were purposively selected. A pre-trialled questionnaire was administered to obtain information on dog ownership and knowledge of rabies, host species affected, modes of transmission, symptoms, recommended treatment and preventive methods.<br>Health practitioners were purposively selected and completed the questionnaire during the investigator's visit. The questionnaire sought information on knowledge of rabies, management of bite wounds, vaccination sites and schedules of pre- and post-exposure prophylaxis. Descriptive and inferential data analyses were performed using SPSS software version 18.0.<br>Results<br>Approximately twenty per cent (18.9%; 95% CI: 14.3 – 24.3) and 13.3% (95% CI: 9.4 – 18.1) of households had good knowledge and practices of rabies respectively. For health practitioners, only 16.7% (95% CI: 7.5 – 31.9) had good knowledge, whilst 33.3% (95% CI: 20.0 – 49.7) adopted adequate attitudes/practices towards the disease.<br><br>Conclusions/significance<br>A lack of community-based education and professional retraining courses contribute significantly to poor awareness of rabies in the LNP of Mozambique. Enhancing public health knowledge should consequently reduce dog-mediated human rabies deaths in this country.
**Additional Information:**	
**Question**	**Response**
**Financial Disclosure**<br><br>Enter a financial disclosure statement that describes the sources of funding for the work included in this submission. Review the submission guidelines for detailed	University of Pretoria and Institute of Tropical Medicine of Antwerp (Project "Communities on the move: animal and human health challenges").<br>The funders had no role in study design, data collection and analysis, decision to publish, or preparation of the manuscript.

requirements. View published research articles from *PLOS NTDs* for specific examples.<br><br>This statement is required for submission and **will appear in the published article** if the submission is accepted. Please make sure it is accurate.<br><br>**Unfunded studies**<br>Enter: *The author(s) received no specific funding for this work.*<br><br>**Funded studies**<br>Enter a statement with the following details:<br>• Initials of the authors who received each award<br>• Grant numbers awarded to each author<br>• The full name of each funder<br>• URL of each funder website<br>• Did the sponsors or funders play any role in the study design, data collection and analysis, decision to publish, or preparation of the manuscript?<br>• **NO** - Include this sentence at the end of your statement: *The funders had no role in study design, data collection and analysis, decision to publish, or preparation of the manuscript.*<br>• **YES** - Specify the role(s) played.<br><br>* typeset	
**Competing Interests**<br><br>Use the instructions below to enter a competing interest statement for this submission. On behalf of all authors, disclose any competing interests that could be perceived to bias this work—acknowledging all financial support and any other relevant financial or non-	The authors have declared that no competing interests exist.

financial competing interests.<br><br>This statement **will appear in the published article** if the submission is accepted. Please make sure it is accurate. View published research articles from *PLOS NTDs* for specific examples.<br><br>**NO authors have competing interests**<br><br>Enter: *The authors have declared that no competing interests exist.*<br><br>**Authors with competing interests**<br><br>Enter competing interest details beginning with this statement:<br><br>*I have read the journal's policy and the authors of this manuscript have the following competing interests: [insert competing interests here]*<br><br>* typeset<br><br>This statement is required for submission and **will appear in the published article** if the submission is accepted. Please make sure it is accurate and that any funding sources listed in your Funding Information later in the submission form are also declared in your Financial Disclosure statement.	
**Data Availability**	Yes - all data are fully available without restriction

Authors are required to make all data underlying the findings described fully available, without restriction, and from the time of publication. PLOS allows rare exceptions to address legal and ethical concerns. See the PLOS Data Policy and FAQ for detailed information.<br><br>A Data Availability Statement describing where the data can be found is required at submission. Your answers to this question constitute the Data Availability Statement and **will be published in the article**, if accepted.<br><br>**Important:** Stating 'data available on request from the author' is not sufficient. If your data are only available upon request, select 'No' for the first question and explain your exceptional situation in the text box.<br><br>Do the authors confirm that all data underlying the findings described in their manuscript are fully available without restriction?	
**Describe where the data may be found in full sentences. If you are copying our sample text, replace any instances of XXX with the appropriate details.**<br><br>• If the data are **held or will be held in a public repository**, include URLs, accession numbers or DOIs. If this information will only be available after acceptance, indicate this by ticking the box below. For example: *All XXX files are available from the XXX database (accession number(s) XXX, XXX.*).<br>• If the data are all contained **within the**	All relevant data are within the manuscript and its Supporting Information files.

Powered by Editorial Manager® and ProduXion Manager® from Aries Systems Corporation

**manuscript and/or Supporting Information files**, enter the following: *All relevant data are within the manuscript and its Supporting Information files.*<br>• If neither of these applies but you are able to provide **details of access elsewhere**, with or without limitations, please do so. For example:<br><br>*Data cannot be shared publicly because of [XXX]. Data are available from the XXX Institutional Data Access / Ethics Committee (contact via XXX) for researchers who meet the criteria for access to confidential data.*<br><br>*The data underlying the results presented in the study are available from (include the name of the third party and contact information or URL).*<br>• This text is appropriate if the data are owned by a third party and authors do not have permission to share the data.<br><br>* typeset	
Additional data availability information:	

Powered by Editorial Manager® and ProduXion Manager® from Aries Systems Corporation

II clean version Click here to access/download;Manuscript;PNTD-D-20-01733 KAPs Rabies II Clean Version.docx

Knowledge, attitudes, practices (KAPs) and control of rabies among community households and health practitioners at the human-wildlife interface in Limpopo National Park, Massingir District, Mozambique

Milton Mapatse[1*], Claude Sabeta[2,3], José Fafetine[1,4], Darrell Abernethy[5,6]

[1] Veterinary Faculty, University Eduardo Mondlane, Maputo, Mozambique

[2] Agricultural Research Council-Onderstepoort Veterinary Institute. OIE Rabies Reference Laboratory, Pretoria, South Africa

[3] University of Pretoria, Department of Veterinary Tropical Diseases,, Pretoria, South Africa

[4] Centre of Biotechnology (CB-UEM), Maputo, Mozambique

[5] Centre for Veterinary Wildlife Studies, University of Pretoria, Pretoria, South Africa

[6] Aberystwyth School of Veterinary Science, Institute of Biological, Environmental and Rural Sciences, Aberystwyth University, Aberystwyth, SY 23 3EB, United Kingdom

***** Corresponding author

E-mail: miltonkitovet@gmail.com

Abstract

Background

Rabies is a viral zoonotic disease that kills more than 26,000 people each year in Africa. In Mozambique, poverty and inadequate surveillance result in gross underreporting and ineffective control of the disease in animals and people. Little is known of the role of human attitudes and behaviour in prevention or control of rabies, thus this study was undertaken to assess the knowledge, attitudes and practices amongst selected

households and health practitioners in one affected area, the Limpopo National Park (LNP), Massingir district.

Methodology

A cross-sectional study was conducted of 233 households in eight villages in LNP and among 42 health practitioners from eight health facilities in Massingir between 2016 and 2018. Permission for the study was first obtained from village leaders before consenting household representatives aged 18 years or over were purposively selected. A pre-trialled questionnaire was administered to obtain information on dog ownership and knowledge of rabies, host species affected, modes of transmission, symptoms, recommended treatment and preventive methods.

Health practitioners were purposively selected and completed the questionnaire during the investigator's visit. The questionnaire sought information on knowledge of rabies, management of bite wounds, vaccination sites and schedules of pre- and post-exposure prophylaxis. Descriptive and inferential data analyses were performed using SPSS software version 18.0.

Results

Approximately twenty per cent (18.9%; 95% CI: 14.3 – 24.3) and 13.3% (95% CI: 9.4 – 18.1) of households had good knowledge and practices of rabies respectively. For health practitioners, only 16.7% (95% CI: 7.5 – 31.9) had good knowledge, whilst 33.3% (95% CI: 20.0 – 49.7) adopted adequate attitudes/practices towards the disease.

Conclusions/significance

A lack of community-based education and professional retraining courses contribute significantly to poor awareness of rabies in the LNP of Mozambique. Enhancing public health knowledge should consequently reduce dog-mediated human rabies deaths in this country.

Author summary

In Mozambique, rabies is maintained primarily by the domestic dog, the principal vector and host species responsible for the majority of human cases dating back to the early 1900s. Control of animal rabies has historically been undertaken by government veterinary authorities, with limited involvement of the health and environment sectors. In the Massingir District in general, and in Limpopo National Park (LNP) in particular, parenteral dog rabies mass-vaccination campaigns and the provision of post-exposure prophylaxis are inconsistent. Limited resources for dog vaccination campaigns, insufficient veterinary field staff, inefficient disease notification procedures and inadequate training of health practitioners constraint effective rabies control across the country. Awareness of good practices regarding management of bite wounds among local community members and health practitioners is crucial to reducing rabies deaths. The results obtained in this study will inform government policy on practical interventions in the control of dog and human rabies.

Introduction

Rabies is one of the oldest infectious diseases in medical history, caused by a member of the *Lyssavirus* genus and which can potentially infect all warm-blooded vertebrates, including humans. Today, rabies remains a major public and veterinary health problem in most developing countries, particularly in the tropical and subtropical regions of

Africa and Asia [1,2] where it is considered a neglected but re-emerging disease [3,4]. Upon entry into a susceptible host, the highly neurotropic virus induces an acute, progressive and fatal encephalomyelitis, mainly through bites but also, to a lesser extent, through scratches or contact with mucous membranes [5]. The causative agent, responsible for more than 95% of human rabies deaths, is a classical rabies virus (RABV) and one of the 17 members in the genus *Lyssavirus*, family *Rhabdoviridae* [6-8]. Members of this genus are characterized by non-segmented negative-sense RNA genomes of approximately 12 kb in size [9]. Globally, human mortality is estimated to be at least 59 000 deaths per year, 56% of these occurring in Asia and 44% in Africa, especially among rural communal areas with large stray dog populations [10-12] and inconsistent dog mass vaccinations. Poverty and poor awareness of rabies are generally associated with an increased vulnerability to the disease and are, consequently, major obstacles in prevention and control, especially in rural areas [13,14]. A general understanding about dog behaviour, responsible pet ownership, appropriate health service-seeking behaviour following dog bites and rabies prevention are all crucial in raising public awareness and reducing the number of deaths [15,16]. The National Strategy for the Control of Rabies in Mozambique was approved by the Council of Ministers in 2010 to control rabies in animal populations by limiting its expansion and subsequent spill over into humans. Despite this initiative, rabies deaths continue to be reported annually from all provinces of this country [17,18]. Between 1989 and 2007, 1,001 human cases were reported in Mozambique with a two-fold increase in deaths from 2010 (n=44) to 2016 (n=94) and 2017 (n=89) [19]. Furthermore, in the last four decades, there has been a general population movement to occupy some of the conservation areas, including Massingir [20]. These remote communities now reside at the human-wildlife interfaces with their dogs, exposed to the threat of rabies from wild

species such as African wild dogs, bat-eared fox, jackals, mongoose and hyenas [17,21, 22, 23]. The risk of rabies was described by Osofsky et al. [24] using the LNP as an example, where a the lack of general disease control including the parenteral vaccination of dogs, was of critical importance.

There is a paucity of data in Mozambique on the knowledge of, and attitudes to, rabies [25,26]. Similar to other African countries, children under 15 years are the main victims of rabies, but are not aware of preventive actions following bite contacts with rabies-suspect dogs [27,28]. The majority of victims seek help from traditional healers rather than health treatment centres [29,30] and suspect rabid dogs are destroyed without quarantine [31-33] or submitting relevant samples for laboratory confirmation. In Tanzania for example, bite management centres have been set up and have significantly improved the surveillance of rabies in dogs [34,35]. Enhancing public health awareness in the complex socio-ecological systems of protected wildlife is a prerequisite to addressing the identified gaps, cultural beliefs and behavioural patterns. Similarly, designing relevant public health educational campaigns, supported by appropriate planning, implementation and evaluation of national control programmes [36,37] will assist in the elimination of dog-mediated human rabies. The Mozambique National Health Plan was designed to strengthen primary health care but the lack of adequate health facilities, poor infrastructure and inadequate training of medical staff, particularly on the management of dog bites and rabies cases [38], undermines its objectives. In the Massingir District, the ratio of medical doctors is similar to the rest of the country (1 physician per 10 000 population versus 0.7 per 10 000 respectively; 2018 data), implying that healthcare services are mostly provided by technicians with elementary to intermediate skills in medical care [39,40].

It is crucial that caregivers, both household members and health professionals, have the correct level of knowledge and ability to prevent rabies and manage animal bites when they occur. Hence, the objectives of this study were to determine the awareness, knowledge, attitudes and practices and awareness of rabies, including prevention and control measures applied to the disease on the ground among selected households and healthcare practitioners in a human-wildlife interface area, the Limpopo National Park (LNP).

Materials and Methods

Characteristics of the study area and population

The study was undertaken in the Limpopo National Park (LNP, Fig 1), Mozambique, between 2016 and 2018. The LNP is located to the west of Gaza Province and is delimited by 190 km of fenced international border with South Africa (Kruger National Park) to the west and by both Limpopo (about 260 km) and Elefantes (about 85 km) rivers in the east and south, respectively [41,42]. The LNP includes 13 villages situated along three major rivers, namely the Limpopo River (Macaringue, Munhamane, Cunze, Chipanzo and Maconguele villages); the Shingwedzi River (Bingo, Machamba, Chimangue and Mavoze villages) and Elefantes River (Madingane, Mahlaúle, Chibotane and Macuachane villages). Three villages (Machamba, Bingo and Mavoze) are located inside the national park with the remainder in the Buffer Zone, an area considered to be within the park boundaries. These settlements comprise agricultural lands (crop) and 70% of the livestock [43]. The Buffer Zone extends westwards from the Limpopo River for eight to ten kilometres and northwards from the Elefantes River in the area between the latter's confluence with the Limpopo and Massingir Dam [44].

Fig 1. Limpopo National Park Map showing the study area (villages included in the study are indicated by rectangles).

The human population of the LNP was estimated in 2001 at approximately 27,000 [45] with most inhabitants concentrated in areas with arable alluvial soils [44]. Based on a 2015 census of animals by the District Services of Economic Activities of Massingir, there were approximately 38,000 cattle, 19,000 goats; 7,000 sheep; 2,000 pigs; 1,500 dogs and 51 donkeys in the LNP.

There are eight health facilities in Massingir District, three inside the park, three in the Buffer Zone and two outside [86] (Fig 2).

Fig 2. Location of all eight Health Facilities of Massingir District [86]

Study design and sampling framework

Household (HHs) Survey

A cross-sectional study was undertaken among 233 households selected within eight of the 13 LNP villages between 2016 and 2018. The villages were selected using stratified multi-stage random sampling. A sampling frame of all the villages was constructed and the “RAND” function in Microsoft Excel 2010 used to generate random entries. The selected villages were Machamba, Bingo and Mavoze inside the park and Macaringue, Mahlaúle, Madingane, Cunze and Munhamane in the buffer zone.

Sample size

The sample size was calculated using the single population proportion formula as described by Dohoo et al. [46], assuming an estimated weighted measure (87.0%) of

respondents with knowledge of rabies based on two previous community-based cross-sectional studies of rabies awareness in central and northern Mozambique [25,47], a 95.0% confidence interval, 5% margin of error and 80.0% power. With 1968 households in the study area [48], the sample size representative of the Limpopo National Park region was estimated to be 160. The non-response rate was estimated to be 9.0% and an additional 59 households were added due to positive responses to the interviewer in the villages, providing a total of 233. 233 . The weighted sample per village was estimated using the following formula:

w = n*nh/tnh

Where: w = weighted sample per village; n = required sample size (n = 174); nh -total of households per village; tnh – total number of households

Recruitment and interviews

Pre-survey visits were undertaken to obtain consent from village leaders and to request their assistance in mobilising their communities to participate in the study. Purposive sampling was used to select households. Community leaders identified representatives of each household, to whom the study team explained the objectives of the study before obtaining their oral consent. Respondents had to be 18 years of age or older and available to participate at the time of the visit. Face-to-face interviews were conducted in Changana, the local language spoken in Massingir region, for appropriateness and ease among study participants. The answers were, however, recorded in Portuguese.

Health practitioner (HPs) survey

A cross-sectional study was conducted among all eight health facilities in Massingir District, Gaza Province (2016 to 2018) and included all 42 working personnel, comprised of two doctors, eight physician's assistants, 19 nurses, 11 caregivers and two technicians. Participants were purposively selected [49] among those who deal directly with patients on a daily basis (clinical, nursing and laboratory personnel). The lead investigator personally visited the HPs at their workplaces, explained the purpose of the study and assured them of confidentiality with regard to their responses. After oral consent was granted, they then proceeded to complete the self-administered questionnaires.

Ethics statement

The study was approved by the Biotechnology Centre scientific board (University Eduardo Mondlane, Mozambique) (21.10.16) and by the Animal Ethics Committee of University of Pretoria (V133-16).

Data entry and analysis

Two databases were created for the two study populations in Epi-Info, version 7 (CDC, USA). Data were analysed using SPSS Statistics for Windows software, version 18.0 (SPSS Inc., Chicago, Ill., USA). The analysis focused on descriptive statistics to determine the frequency, proportions, medians and means of the responses regarding both target groups (households and health practitioners).

To assess the dimension of responses (knowledge, attitudes and practices), all potential factors were grouped and scored as in the scoring systems used in similar previous studies [37,50-52], with some slight modifications. The scoring systems used measured the completeness and accuracy of respondents' answers according to the nature of the question.

Depending on the type of question, scores ranged from 0 to 3. For example, for questions on households' knowledge assessment, the maximum overall score stipulated was 13, while it was 10 and 8 respectively, for attitudes and practices. Knowledge scores were categorized into "Poor" (0-6) and "Good" (7-13); attitudes into "Negative" (0-5) and "Positive" (6-10) and practice scores into "Poor" (0-3) and "Good" (4-8). For health practitioners, the maximum overall score was 27 for "knowledge" and five for attitudes/practices. Knowledge scores regarding health practitioners were categorized into "Poor" (0-13) and "Good" (14-27), and attitudes/practices into "Inadequate" (0-2) and "Adequate" (3-5).

The KAP on rabies (dependent variables) were compared against HH respondents' village, gender, educational level, and occupation, health practitioners' health post, years of service and occupation (independent variables). Inferential analysis was done using the Chi-square test. All analyses were performed using 95% confidence intervals and *P* value <.05 considered as statistically significant.

Results

Households (HHs) characteristics and dog ownership

The socio-demographic characteristics of households are shown in Table 1. The majority of interviewees were male (63.1%). Most participants were aged between 50 and 59 years, comprising 40.3% of study population. Over 60.0% of respondents had at least a primary school education and 82.8% were farmers. Nine in ten of the households across all villages owned at least one dog 89.3% and of these, 20.2% of the dogs were sterilized. The most frequent reason for keeping dogs was to provide protection of the crop fields against monkeys (68.8%) while the least popular reason was for a companion pet (1.0%).

Table 1. Socio-demographic characteristics of the study participants in LNP, Mozambique

Variables		Frequency (%)
Gender	Male	147 (63.1)
	Female	86 (36.9)
Age (years)	≤ 19	27 (11.6)
	20-29	34 (14.6)
	30-39	41 (17.6)
	40-49	33 (14.2)
	50-59	94 (40.3)
	≥ 60	4 (1.7)
Education	None	85 (36.5)
	Primary	141 (60.5)
	Secondary	7 (3.0)
Occupation	Farmer	193 (82.8)
	Non-farmer	40 (7.2)
Reasons for keeping a dog	Protect crop fields against monkeys	143 (68.8)
	Security	45 (21.6)
	Herding	30 (14.4)
	Hunting	10 (4.8)
	As pet	2 (1.0)

Dog confinement, community dog bite experience and healthcare-seeking behaviour

Of households owning dogs, 80.3% never confined the dogs, while 3.4% restricted them to a yard or enclosure. There was no statistical difference in the proportion of households with a single dog (22.2%) that confined them, compared to those with multiple dogs (16.5%) (*P* =.605).

Most respondents (91.0%) would first seek help at health centres for the treatment of bite wounds while 4.7% would consult traditional healers. At the home level, 64.8% explained that they would wash the bite wounds with water, while 24.5% correctly

indicated that they would wash the bite wounds with soapy water. The results on bite cases experienced in the previous 12 months, the respective measures taken by victims, as well as the attitudes towards future bite wounds and rabies prevention, are provided in Table 2. Of all respondents, 17.6% recalled a family member having been bitten by a dog in the previous 12 months. A few victims (12.2%) sought post-exposure vaccination. In most cases (56.1%), a nurse provided such treatment, while a traditional healer assisted in four cases (9.8%). Where the respondent knew the status of the dog after the incident (90.2%), in 35.1% of cases the dog had died while in the majority of cases (64.9%), the dogs had remained healthy.

Table 2. Dog bite experience, treatment applied and health-seeking behaviours to be adopted within households

Variables		Frequency (%)
Source of biting dog in previous 12 months	Neighbours' dogs	27 (65.9)
	Household's dogs	13 (31.7)
	Unidentified strange dog	1 (2.4)
Treatment applied after bite	Local bite area treatment	28 (68.3)
	Rabies PEP	5 (12.2)
	Traditional treatment	4 (9.8)
	Medical wound treatment	3 (7.3)
	Other	3 (7.3)
	Nothing	1 (2.4)
	Not known	1 (2.4)
Initial site for first aid for dog bites	Health centre	212 (91.0)
	Traditional healer	11 (4.7)
	Home level + health centre	4 (1.7)
	Home level	3 (1.3)
	None	3 (1.3)
First aid actions at home level if bitten	Wash wound with water	151 (64.8)
	Wash wound with water + soap	57 (24.5)
	Traditional/spiritual medicine	13 (5.6)
	Do nothing	12 (5.2)

Animal health care seeking behaviour including dog vaccination status

Approximately one third (30.8%) of households who owned dogs visited an animal care health centre at least once a year. There was no statistical difference in the proportion of households who visited the animal health officer based on whether they had one or multiple dogs (29.7% versus 31.3% respectively; *P*=.829). The most common reason for visiting was for dog vaccination (82.8% of visits), while visits prompted by a dog being rabies-suspect or for aggressive behaviour accounted for only 9.4% . The extent of dog vaccination (using anti-rabies vaccination certificates as proof or laboratory results of rabies antibody levels) differed significantly among villages, with Mavoze having the highest proportion of vaccinated dogs (6.0%; *P*=.001) and Machamba (0) the lowest [(data presented as supporting information (S1). The most frequent reason for non-vaccination was lack of information of a campaign (62.1%), followed by absence during a campaign (10.1%) and not being aware of the need to vaccinate (9.5%).

Households' rabies awareness

Knowledge of rabies, its transmission, clinical signs and disease outcome are presented as supporting information (S2). In summary, 97.9% of the respondents had heard of rabies and 51.8% of these had heard of this through family elders. When asked about the source of rabies, 96.5% of the respondents correctly identified dogs, while 4.8% mentioned cats, but significantly fewer correctly identified other species to be susceptible to rabies virus infection (Fig 3). Twenty-three (10.1%) respondents correctly identified a virus as the causative agent of rabies, whilst 31.1% incorrectly believed the disease was of psychological origin. Almost all respondents (98.2%) stated correctly that dogs could be affected by rabies, but significantly fewer correctly identified other

warm-blooded species as being susceptible to rabies virus infection. More than eighty per cent (82.9%) of households were aware of the zoonotic nature of rabies (Fig 3). When asked about the mode of rabies transmission to humans, a significant majority of respondents (88.4%) correctly mentioned bites from an infected animal as an important transmission route . Ninety percent of respondents stated they had observed rabies in animals while 1.5% indicated they had seen cases of human rabies. All participants correctly identified rabid dogs as the most common species observed with clinical signs of the disease. Aggression and aimless wandering, accounted for 50.0% of all observed symptoms and were the most commonly reported signs (Fig 4). Of all study respondents, 82.0% were aware that rabies is fatal following the onset of clinical signs.

Fig 3. Frequency of households' responses regarding animals affected by rabies

Fig 4. Frequency of households' responses regarding symptoms observed in rabies cases

Knowledge and attitudes towards rabies prevention

Results on the prevention of rabies are presented as supporting information (S3). In brief, 54.8% of respondents mentioned rabies was preventable through parenteral dog vaccination. Half of the participants were aware of the inevitable fatality of the disease following onset of symptoms while 25.9% incorrectly thought it was curable through unspecified oral or injectable drugs. Only 1.6% of respondents were aware of animal population control (confining dogs) as one of the preventive measures. When asked about practices adopted for animals with behaviour suggestive of rabies, 96.9% of respondents said that they would either kill or expel them from their homes or neighbourhood, while 3.5% would not do anything. Almost all (96.2%) respondents who owned dogs expressed their willingness to register their dogs. Nearly all (99.1%)

were willing to participate in future rabies vaccination campaigns and of these, 95.3% would seek help from health or veterinary authorities if bitten by a suspect rabid dog. A high proportion (93.0%) of the respondents said they would euthanize their dog if it were rabid.

Level of knowledge, attitudes and practices towards rabies within households

In general, respondents had a good knowledge (18.9%) and practices (13.3%) on rabies, and a positive attitude (87.1%) towards the disease.

Table 3 shows statistically significant differences (*P* <.05) between knowledge of rabies, location of villages and educational level of the respondents. Respondents living in Mavoze village, for example, demonstrated a good knowledge (27.9%), adopted positive attitudes (90.7%) and most (25.6%) implemented good practices towards rabies. No households in Madingane and only 3.4% in Bingo had good knowledge and good practices towards rabies, respectively. More male respondents had good knowledge (19.0%), positive attitudes (88.4%) and good practices (13.6%) than females (18.6%, 84.9% and 12.8%, respectively). The 30 to 39 years age group had the most respondents with 22.0% demonstrating good knowledge of rabies. For attitudes and good practices, the best results were observed in individuals between 20-29 years (91.2%) and 50-59 years (19.1%). It appeared that those with secondary education, showed good knowledge (28.6%) and positive attitudes (100.0%) towards rabies. Only 10.6% of the respondents without education and none with secondary education, adopted good practices in relation to rabies. Eight non-farmers (20.0%) had the best level of knowledge of rabies, while only 10.0% displayed good practices.

Table 3 Knowledge, attitudes and practices on rabies of the study participants in LNP based on location, gender, age, education and occupation

Factors	Knowledge			Attitudes			Practices		
	Good (%)	Poor (%)	*P* value	Positive (%)	Negative (%)	*P* value	Good (%)	Poor (%)	*P* value
Villages									
Macaringue	12 (29.3)	29 (70.7)	.032	35 (85.4)	6 (14.6)	.910	5 (12.2)	36 (87.8)	.133
Machamba	6 (26.1)	17 (73.9)		20 (87.0)	3 (13.0)		1 (4.3)	22 (95.7)	
Munhamane	5 (17.2)	24 (82.8)		25 (86.2)	4 (13.8)		5 (17.2)	24 (82.8)	
Mavoze	12 (27.9)	31 (72.1)		39 (90.7)	4 (9.3)		11 (25.6)	32 (74.4)	
Madingane	0 (0)	26 (100)		21 (80.8)	5 (19.2)		3 (11.5)	23 (88.5)	
Mahlaúle	1 (5.9)	16 (94.1)		15 (88.2	2 (11.8)		1 (5.9)	16 (94.1)	
Cunze	5 (20.0)	20 (80.0)		21 (84.0)	4 (16.0)		4 (16.0)	21 (84.0)	
Bingo	3 (10.3)	26 (89.7)		27 (93.1)	2 (6.9)		1 (3.4)	28 (96.6)	
Gender									
Male	28 (19.0)	119 (81.0)	.934	130 (88.4)	17 (11.6)	.435	20 (13.6)	127 (86.4)	.860
Female	16 (18.6)	70 (81.4)		73 (84.9)	13 (15.1)		11 (12.8)	75 (87.2)	
Age in years									
≤ 19	4 (14.8)	35 (85.2)	.847	24 (88.9)	3 (11.1)	.931	0 (0)	27 (100.0)	.142
20-29	6 (17.6)	28 (82.4)		31 (91.2)	3 (8.8)		3 (8.8)	31 (91.2)	
30-39	9 (22.0)	32 (78.0)		36 (87.8)	5 (12.2)		5 (12.2)	36 (87.8)	
40-49	4 (12.1)	29 (87.9)		28 (84.8)	5 (15.2)		5 (15.2)	28 (84.8)	
50-59	20 (21.3)	74 (78.7)		81 (86.2)	13 (13.8)		18 (19.1)	76 (80.9)	
≥ 60	1 (25.0)	3 (75.0)		3 (75.0)	1 (25.0)		0 (0)	4 (100)	
Education									

Primary	35 (24.8)	106 (75.2)	.007	123 (87.2)	18 (12.8)	.562	22 (15.6)	119 (84.4)	.322
Secondary	2 (28.6)	5 (71.4)		7 (100)	0 (0)		0 (0)	7 (100)	
None	7 (8.2)	78 (91.8)		73 (85.9)	12 (14.1)		9 (10.6)	76 (89.4)	
Occupation									
Farmer	36 (18.7)	157 (81.3)	.843	170 (88.1)	23 (11.9)	.337	27 (14.0)	166 (86.0)	.499
Non-farmer	8 (20.0)	32 (80.0)		33 (82.5)	7 (17.5)		4 (10.0)	36 (90.0)	

Health Practitioners (HPs)

Forty-two medical staff from eight health centres completed the questionnaire. Nurses comprised the greatest proportion (45.0%) (Fig 5). Twenty-four (57.1%) of the respondents were female. The average length of service was 4.57 years (median: 2 years; range: 1 to 30 years).

Fig 5. Professional role of Health practitioner respondents of Massingir district

Health practitioners' knowledge of rabies

HPs' knowledge of the causative agent, principal sources of rabies in Mozambique, mode of transmissions, clinical features and incubation period are outlined in the supporting information (S4). In brief, 76.2% of participants correctly identified rabies virus as the causative agent of the disease, while 85.7% correctly stated the dog as the most important source of rabies in Mozambique. Of those who incorrectly identified the source, five were in their first year and one was in the second year of service. When asked about transmission routes of the disease, 88.1% correctly identified dog bites as the important transmission route. However, only 26.2% correctly identified saliva and 2.4% suggested raw meat as a possible threat. Almost all (95.2%) respondents believed they knew symptoms associated with rabies and most (87.5%) knew the disease was fatal. However, only 40.0% correctly identified the early, flu-like symptoms of rabies in humans. Similarly, 50.0% of interviewees correctly indicated hydrophobia as a symptom but only 14.3% identified paralysis. In all these cases, there was no significant

difference between those with more advanced training (e.g. nurses) and the assistants (P >.05).

Health practitioners' knowledge of wound categorization

Twenty nine (69.0%) of the 42 interviewees confirmed they knew about the World Health Organization (WHO) classification of rabies bite exposures and, of these, most (65.5%) correctly identified mild (Category I) exposure. However, only three (10.3%) correctly identified all the three exposure categories (Table 4). Over half (57.1%) were not aware about the relationship between the site/severity of the bite wound and the period of RABV incubation.

Table 4. Health practitioners' knowledge of wound categorization in Massingir District

**Variables**	**Frequency (%)**				**Guidelines**
	**CAT I**	**CAT II**	**CAT III**	**Don't know**	
Rabies exposure categories					
Touching or feeding animals, licks on intact skin	19 (65.5)	0	0	23 (35.5)	CAT I
Nibbling of uncovered skin, minor scratches/abrasions without bleeding	0	9 (27.6)	0	33 (73.4)	CAT II
Single transdermal bites or scratches, licks on broken skin	0	0	11 (34.5)	31 (66.5)	CAT III
Multiple transdermal bites or scratches, licks on broken skin	0	0	17 (58.6)	25 (42.4)	
Contamination of mucous membranes or broken skin with saliva	0	0	8 (27.6)	34 (73.4)	

Health practitioners' practices towards wound bite

Thirty-eight (90.5%) respondents confirmed they knew about wound management. Thirty-seven (97.4%) suggested that bite wounds should be washed with soapy water, while 18.4% felt that antiseptic was necessary. Interviewees with more than two years' experience (33.3%) were more likely to know this than their less experienced counterparts (12.5%) (*P* = .136) (Fig 6).

Fig 6. Health practitioners' practices regarding bite wound practices (n=42)

Thirty-four HPs (89.4%) were aware of the advice that should be given to patients exposed to a dog suspected of rabies. However, only thirteen (30.9%) correctly identified isolating the suspect-rabid dogs for a period of up to ten days. A significantly higher proportion (56.3%) of interviewees with more than two years experience were more likely to know this than their less experienced colleagues (22.2%) (*P* = .044).

Knowledge of rabies prevention methods

Regarding rabies prevention, most respondents (83.3%) correctly reported that parenteral dog vaccination was an important preventive measure as dogs were the source of the disease. Fewer study participants identified other useful measures such as destruction of suspect animals (33.3%), public education (26.2%) or pre-exposure prophylaxis (16.7%).

Knowledge of WHO guidelines for the recommended route(s) and site(s) for the anti-rabies vaccination

Based on the WHO recommendations for the route and sites for administration of the rabies vaccine, 59.5% of HPs stated correctly that the rabies vaccine should be administered via the intramuscular (IM) route, while 21.4% correctly reported intradermal (ID) route as an alternative method. On the other hand, 57.1% of HPs, correctly answered that the site of application of the anti-rabies vaccine was the deltoid muscle and 21.4% also mentioned correctly the thigh as the other region.

Knowledge of post-exposure prophylaxis (PEP) management according to the category of exposure

When asked about the best strategy to handle Category I, II and III patients, relatively few respondents identified the correct approaches. For Category I – 7.1% , for Category II – 16.7% and for Category III – 28.6% (Table 5).

Table 5. Health practitioners who answered correctly about WHO recommended rabies treatment

Variables*	CAT I	CAT II	CAT III	Guidelines
N	3 (7.1%)	-	-	CAT I (N)
WM+V	-	7 (16.7%)	-	CAT II (WM+V)
WM+V+RIG	-	-	12 (28.6%)	CAT III (WM+V+RIG)

*Variables (N-Nothing; WM-Wound management; V-Vaccine; RIG-Rabies immunoglobulin)

Level of knowledge, attitudes/practices within health practitioners

Of the 42 interviewees, 16.7% respondents had a good knowledge, whilst 33.3% adopted adequate attitudes/practices towards the disease.

Associations between variables under study within health practitioners

The results in Table 6 show statistically significant differences between knowledge of rabies, gender and occupation. One respondent (100.0%) of Machamba and Cubo Health posts demonstrated a good knowledge and adopted adequate attitudes/practices of rabies, respectively. There were more male respondents who had good knowledge (33.3%) and adequate attitudes/practices (50.0%) than females. The 36-40 year age group was the one with the most respondents (50%) and demonstrated good knowledge of rabies, while for attitudes/practices, best results were observed in the 31-35 year old individuals (66.7%), respectively. With regard to years of service, those with 11-15 years of practice (100.0%) had good knowledge and 100.0% , HPs with 11 years onwards adopted adequate attitudes/practices towards rabies. All physicians (100.0%) and 62.5% physician assistant were knowledgeable and had adequate attitudes/practices of rabies.

Table 6. HPs' level of knowledge, attitudes and practices towards rabies, according to location, gender, age, years of service and occupation, in Massingir district, Mozambique

Factors	Knowledge				Attitudes/Practices	
	Good (%)	Poor (%)	*P* value	Adequate (%)	Inadequate (%)	*P* value
Villages						
Macaringue	0 (0)	4 (100)	.226	2 (50.0)	2 (50.0)	.283
Machamba	1 (100)	0 (0)		0 (0)	1 (100)	
Massingir-Sede	5 (22.7)	17 (77.3)		8 (36.4)	14 (63.6)	
Mavoze	0 (0)	2 (100)		0 (0)	2 (100)	
Cubo	0 (0)	1 (100)		1 (100)	0 (0)	
Chibotane	0 (0)	3 (100)		2 (66.7)	1 (33.3)	
Mucatine	0 (0)	6 (100)		0 (0)	6 (100)	
Zulo	1 (33.3)	2 (66.7)		1 (33.3)	2 (66.7)	
Gender						
Male	6 (33.3)	12 (66.7)	.031	9 (50.0)	9 (50.0)	.096
Female	1 (4.2)	23 (95.8)		5 (20.8)	19 (79.2)	
Age						
18-24	1 (9.1)	10 (90.9)	.549	2 (18.2)	9 (81.8)	.396
25-30	4 (16.4)	20 (83.3)		9 (37.5)	15 (62.5)	
31-35	1 (33.3)	2 (66.7)		2 (66.7)	1 (33.3)	
36-40	1 (50.0)	1 (50.0)		1 (50.0)	1 (50.0)	
>40	0 (0)	2 (100)		0 (0)	2 (100)	
Years of service						
1-5	4 (12.9)	27(87.1)	.085	9 (29.0)	22 (71.0)	.078
6-10	2 (28.6)	5 (71.4)		5 (71.4)	2 (28.6)	

11-15	1 (100)	0 (0)		0 (0)	1 (100)	
> 15	0 (0)	3 (100)		0 (0)	3 (100)	
Occupation						
Physician	2 (100)	0 (0)	.019	1 (50.0)	1 (50.0)	.241
Physician assistant	2 (25.0)	6 (75.0)		5 (62.5)	3 (37.5)	
Nurse	2 (10.5)	17 (89.5)		6 (31.6)	13 (68.4)	
Care assistant	1 (9.1)	10 (90.9)		2 (18.2)	9 (81.8)	
Technician	0 (0)	2 (100)		0 (0)	2 (100)	

Discussion

Establishing effective rabies prevention and control programs involves an assessment of levels of knowledge, attitudes and practices regarding the disease within local communities [53,54]. For example, knowledge about rabies being a preventable disease is important as it can assist in mitigating risks. However, this must be coupled with adequate practice, which is insufficient occur in Mozambique, where in 2018 for example, the average vaccination coverage was 8.2% of the 3 million animals at risk [55], significantly less than the target of 70% set by the WHO to reach adequate herd immunity.

The low number of respondents with sterilised dogs demonstrated not only a lack of awareness of the importance of this procedure on animal rabies control [56], but equally importantly, a lack of financial and logistical resources among LNP communities to mobilise sterilisation campaigns. According to Kitala et al. [57] surgical sterilization of dogs is a specialized procedure and conceivably out of reach of most of rural populations. Nevertheless, the new strategic plan of the Mozambican Ministry of Agriculture and Rural Development (2020-2024) already foresees the sterilisation of dogs.

Most LNP dogs spend their time roaming freely, foraging for food, but always returning to their household of origin. The low socio-economic status, cultural and rural settlement (houses with no fences) characteristics of the LNP communities also mitigate against confinement of dogs. These practices are similar to those reported elsewhere, for example, in Kenya (69.0%) [57,58], 79.0% in Madagascar [59] and 55.0% in Mali [60]. Confinement, rather than a measure of responsible dog ownership, can also minimize

the risk of contracting or spreading rabies, in addition to helping to plan more effective parenteral vaccination campaigns.

The general willingness shown by most respondents to seek treatment from a health centre following dog bite(s) is commendable and a good starting point for mitigating the risk of contracting rabies. However, these actions must be reinforced by effective practices of wound washing with soapy water, including the minimum washing time. In this survey, as also reported from Ethiopia [61,62], few respondents correctly indicated that washing dog-bite wounds with soapy water is a simple first aid measure that substantially reduces (by five-fold) the risk of contracting rabies [63].

Poor knowledge and practices regarding rabies within the LNP communities are a matter of a great concern, especially the lack of knowledge regarding the causative agent, clinical signs and methods of prevention. Awareness and educational campaigns are a key intervention strategy and should be carried out more frequently and consistently in the region. World Rabies Day, for example, could be an opportune event to disseminate specific messages. In LNP and similar to what was reported in Ethiopia by Alie et al. [64] and Digafe et al. [61], the elders of the family play a key role in disseminating knowledge about rabies. Therefore, education campaigns should seek to better educate elders or provide alternative means of raising awareness. Most study respondents correctly mentioned the mode of transmission of the disease to humans, which might reflect that most respondents had a primary school education. Despite this, the role of other species, in particular wild carnivores, was least known, and is a worrying finding given the proximity of these villages to human-wildlife interfaces. A lack of knowledge of clinical signs in both animals and humans was also evident. Although death is the final outcome following the onset of rabies symptoms, greater

awareness and community education on early detection could significantly reduce the risk of exposure and enhance communities' abilities to make early and correct decisions in suspected cases. Although >75.0% of respondents were aware of vaccination of dogs as a preventive measure, this did not result in action as only 20% of respondents were able to demonstrate a vaccination certificate for the previous year. This was consistent with other published studies such as in Kenya [65], Sri Lanka [66] and Rwanda [67] where between 73.9% to 88.1% of the respondents were aware of rabies prevention. Regardless of the reasons behind the low proportion of vaccinated dogs, a minimum target of 70.0% vaccination coverage combined with PEP are necessary prerequisites to eliminate dog-mediated human rabies deaths by 2030. Such a targets can be achieved through joint action of local government and various partners. It should also include community education, for example, via radio, television and community meetings, on the importance of vaccinating dogs as well as free or subsidised vaccines for use in rural areas where the majority of the population cannot cover vaccination costs.

Destruction of rabies-suspect animals is not unique to Mozambique but has also been reported in Tanzania, Kenya and the Democratic Republic of Congo [65,68,69]. This negative practice contributes to under-reporting of rabies cases. Carcasses will not be examined by laboratory personnel and appropriate samples will not be submitted for laboratory testing and confirmation. Cleaveland et al. [68] also pointed out that the elimination of suspect rabies dogs reduces the number of people who should receive the PEP.

Mavoze village demonstrated the best understanding of rabies knowledge and had good attitudes and practices. This likely reflects its status as the most populous and developed of the eight villages in the study. This was similar to findings by Awuni et al. [70] in

Ghana, that highlighted the influence of the location of households on knowledge-related variables. Similarly, studies from multiple countries, for example, Nigeria [71], Brazil [50], Morocco [72] and Ethiopia [64], show the positive association between some level of education and better knowledge of rabies compared to those who never attended school.

Epidemiological surveillance of rabies can be greatly improved if rural veterinary extension services and communities actively collaborate to control the disease. For instance, over 95% of the study population were willing to register and vaccinate their dogs, thus providing opportunities for improved control if government agencies were able to fund and support vaccination campaigns. In LNP, communities gather, with their livestock and dogs, for animal health related events such as vaccination and acaricidal baths points, or in their crop fields. These points can be considered, together with churches and schools, as strategic places for the implementation of community-based awareness and education campaigns.

One objective of our investigation was to raise awareness and subsequently inform politicians and managers in the health sectors about the need for further training and strengthening the skills of HPs on prevention and PrEP/PEP guidelines. The implementation of a One-Health approach to not only rabies, but to other zoonotic diseases, cannot be over-emphasised. The study revealed that nearly a quarter of HPs respondents were not aware of the causative agent of rabies and the major animal source of the disease in Mozambique. Other studies have shown similar results for the “viral” cause (62%-77.5%) in India and in Pakistan [75,76] respectively, while awareness of the dog as the primary vector species responsible for human rabies cases was higher in India (n=100) [77] than in this study (98% versus 85.7%). For a health professional,

mastery of such concepts is essential as they underpin correct decision-making on optimal therapeutic or prophylactic approaches. Again, this underscores the need for training, retraining courses and capacity building actions. There are valuable courses on the Global Alliance for Rabies Control Education Platform (https://rabiesalliance.org) that could be utilised.

It is also crucial for health professionals to be alert to any case report of patients with a history of wound bites, scratches, or other contact with a potentially infectious vector. Apart from death, few respondents mentioned other clinical signs indicative of encephalitis due to rabies. Our findings differed from those of Kishore et al. [52], where hydrophobia was one of the clinical signs most recognized by the health workers of Uttarakhand, India (95.7%). Symptoms of rabies in people may be similar to other encephalitic diseases such as malaria and and HPs must consider the former as a differential diagnosis. Mallewa et al. [78] in Malawi pointed out that a central nervous system infection should not only be attributed to cerebral malaria, although most of the clinical signs are similar to those of rabies. Wound management and rabies PEP depend on appropriate pre-assessment and correct wound classification [79], and the lack of general awareness among HPs in this regard may delay or worsen timeous and appropriate post-exposure measures. The low proportion of HPs who could correctly advise the monitoring of biting animals for ten days may be due to the fact that most LNP houses are not fenced. In addition, the apparent lack of communication and co-ordination between HPs and animal health officers is an obstacle when it comes to the issue of specific recommendations for the confinement and monitoring of the suspect animal. It is the responsibility of the veterinary sector's staff to confine and closely monitor animals suspected of rabies or those responsible for human bite contact. The

lack of a One Health approach in advice given by the HPs to patients was also observed by Malhotra et al. [80, 81], in India, and by Niang et al. [82] in Senegal.

The association between the site of bite and the wound severity (scratch versus deep skin penetration) is likely to affect the outcome of clinical rabies [30]. In the present study, there was a lack of perception of this association. Furthermore, a considerable percentage of study participants were not aware of guidelines on both the site and route of rabies vaccinations, including the pre- and post-exposure prophylaxis scheme. The introduction of refresher and re-training courses on neglected diseases such as rabies may improve their understanding. In Massingir District, less than half of HPs had specific background training in nursing and lacked access to practical training on anti-viral prophylaxis. The weak and slow development of the health sector, including the scarcity of rabies vaccine and RIGs, may also influence the lack of awareness of its use within the health staff. The same was observed in India, (Tiwari et al. [83]) where it was evident that even physicians were not adequately informed about the importance of administering vaccines and human rabies immunoglobulins. In 2004, MISAU (Ministry of Health) launched a rabies handbook edited by Barreto et al.[84], in which all the basic information about the disease was described for health professionals to understand and apply the knowledge. Surprisingly, the health practitioners still have gaps in their knowledge of rabies and its prevention, as well as in the management of bites.

Conclusions and recommendations

Based on this study, we conclude that rabies is truly a neglected disease in Limpopo National Park, in particular, and in Massingir District in general. The rural communities of LNP displayed a poor level of knowledge and adopted bad practices towards rabies and this may increase the risk of rabies in the community. Health practitioners had poor

knowledge, attitudes and practices on the control and prevention of rabies. Lack of community-based education and medical retraining courses were determinant factors contributing to the poor awareness. Local education and public awareness concerning rabies prevention and control, and the management of bite wounds, should be implemented in the short term by LNP veterinary and health authorities especially if the plan is to end human deaths due to dog-mediated rabies by 2030. The results presented in this study should assist LNP governmental and non-governmental entities in continuously improving rabies prevention and control programmes, including permanent community education and awareness campaigns on the importance of adopting responsible dog ownership behaviours and adherence to regular parenteral dog rabies vaccination campaigns. The findings also emphasise the need to invest in training and refresher courses for professionals regarding pre- and post-exposure prophylaxis measures and encouraging exposed patients to confine animals suspected of rabies while seeking medical help and veterinary assistance.

Study limitations

A major limitation of this study was the fact that there is still a tension between the government and the residents of LNP over the resettlement process that was started in 2006. This process created animosity, mistrust and a general lack of openness to anyone who wants to work in such communities (Milgroom and Spierenburg [85]). Furthermore, a low number of health professionals were interviewed, directly reflecting the shortage of human resources in the Massingir district health sector. This aspect has been a decisive factor in delaying the efforts of the Government of Mozambique to expand and improve the provision of basic health services.

Acknowledgements

We are grateful to Dr Sérgio Salomão Bié (Limpopo National Park) for providing some data related to the Limpopo National Park, to Dr Guilhermina Sitoe (Former Health Director of the Massingir District) for allowing us to work at health post/centres of Massingir District. We are thankful to the communities of all the villages and to the health workers of all post and health centers in Massingir, especially to those who orally consented to participate in this study. We thank the Biotechnology Centre for the logistics and to dr Remígio Mungói (District Services for Economic Activities of Massingir), Dr Vlademiro Magaia, Dr Ofélia Nhambirre, André Nhambir and Iara Gomes for their help in the field. We are grateful to Dr Abel Chilundo and Professor Alberto Pondja for assisting in the insertion, organisation and interpretation of the statistical data.

Author Contributions

M M: conceived and wrote the manuscript. C T S: participated in the study design, and helped to write and edit the manuscript. J M F: participated in the project management and helped to write and edit the final manuscript. D A: participated in the study design, statistical analysis, and reviewing of the manuscript.

Conflicts of Interest

The authors declare no conflict of interest. The funders had no influence in the design of the study, in the collection, analyses, or interpretation of data, in the writing of the manuscript, or in the decision to publish the results.

Funding

University of Pretoria and Institute of Tropical Medicine of Antwerp (Project "Communities on the move: animal and human health challenges").

The funders had no role in study design, data collection and analysis, decision to publish, or preparation of the manuscript

References

1. Banyard AC, Horton DL, Freuling C, Müller T, Fooks AR. Control and prevention of canine rabies: The need for building laboratory-based surveillance capacity. Antiviral Research 2013 Jun;98(3):357-64.

2. Chiou HY, Hsieh CH, Jeng CR, Chan FT, Wang HY, Pang VF. Molecular Characterization of Cryptically Circulating Rabies Virus from Ferret Badgers, Taiwan. Emerg Infect Dis 2014 May;20(5):790-8.

3. Daszak P, Cunningham AA, Hyatt AD. Emerging infectious diseases of wildlife--threats to biodiversity and human health. science 2000;287(5452):443-9.

4. Rupprecht CE, Hanlon CA, Hemachudha T. Rabies re-examined. The Lancet Infectious Diseases 2002;2(6):327-43.

5. Mani RS, Madhusudana SN. Laboratory diagnosis of human rabies: recent advances. The Scientific World Journal 2013;2013.

6. Horton DL, McElhinney LM, Marston DA, Wood JL, Russell CA, Lewis N, et al. Quantifying antigenic relationships among the lyssaviruses. J Virol 2010 Nov;84(22):11841-8.

7. Wallace RM, Mehal J, Nakazawa Y, Recuenco S, Bakamutumaho B, Osinubi M, et al. The impact of poverty on dog ownership and access to canine rabies vaccination: results from a knowledge, attitudes and practices survey, Uganda 2013. Infectious diseases of poverty 2017;6(1):97.

8. Weyer J, Szmyd-Potapczuk AV, Blumberg LH, Leman PA, Markotter W, Swanepoel R, et al. Epidemiology of human rabies in South Africa, 1983-2007. Virus Res 2011 Jan;155(1):283-90.

9. Tordo N, Poch O, Ermine A, Keith G+. Primary structure of leader RNA and nucleoprotein genes of the rabies genome: segmented homology with VSV. Nucleic Acids Research 1986;14(6):2671-83.

10. Hampson K, Coudeville L, Lembo T, Sambo M, Kieffer A, Attlan M, et al. Estimating the global burden of endemic canine rabies. PLoS Negl Trop Dis 2015;9.

11. Jackman J, Rowan AN. Free-roaming dogs in developing countries: The benefits of capture, neuter, and return programs. In: Deborah Salem AR, editor. The state of the animals IV.Washington DC: Humane Society Press; 2007. p. 55-78.

12. Singh R, Singh KP, Cherian S, Saminathan M, Kapoor S, Manjunatha Reddy GB, et al. Rabies - epidemiology, pathogenesis, public health concerns and advances in diagnosis and control: a comprehensive review. Vet Q 2017 Dec;37(1):212-51.

13. Dodet B, Goswami A, Gunasekera A, de GF, Jamali S, Montalban C, et al. Rabies awareness in eight Asian countries. Vaccine 2008 Nov 25;26(50):6344-8.

14. Felgate M. An Update from Mozambique. Rabid Bytes 2011;(21):6.

15. Hasanov E, Zeynalova S, Geleishvili M, Maes E, Tongren E, Marshall E, et al. Assessing the impact of public education on a preventable zoonotic disease: rabies. Epidemiol Infect 2018 Jan;146(2):227-35.

16. Nilsson M. Effect of rabies education programs on rabies awareness, attitudes towards dogs and animal welfare among children in Lilongwe, Malawi. Uppsala: Swedish University of Agricultural Sciences; 2014.

17. Government of Mozambique. Strategy for the Control of Rabies (2010-2014). Council of Ministers 2010;42nd Session(23 November):1-19.

18. Ministry of Agriculture and Food Security. National Agriculture Investment Plan 2014-2018. (Comprehensive Africa Agriculture Development Programme) 2014;1-100.

19. Bilaide SF. Retrospective study of animal and human rabies occurences in Mozambique [Monograph] University Eduardo Mondlane. Maputo; 2019.

20. Cuco ES, Liesegang G. Conflito homem fauna bravia: Caso do Parque nacional de Limpopo University Eduardo Mondlane; 2012.

21. Bekker JL, Hoffman LC, Jooste PJ. Wildlife-associated zoonotic diseases in some southern African countries in relation to game meat safety: a review. The Onderstepoort journal of veterinary research 2012 Dec 5;79(1):E1-E12.

22. Vial F, Cleaveland S, Rasmussen G, Haydon DT. Development of vaccination strategies for the management of rabies in African wild dogs. Biological Conservation 2006;131(2):180-92.

23. WHO. Mozambique. Rabies country profile. World Health Organization 2013;https://www.who.int/rabies/epidemiology/Rabies_CP_Mozambique_09_2013.pdf.

24. Osofsky SA, Cumming DHM, Kock MD. Transboundary management of natural resources and the importance of a 'One Health' approach. In: Fearn E, Woods W, editors. State of the Wild 2008-2009: A Global Portrait of Wildlife, Wildlands, and Oceans.Washington DC: Island Press; 2008. p. 89-98.

25. Bragança LFJ. Caracterização do conhecimento da Raiva pelos estudantes da EP2 e Informantes chave na cidade e dois distritos da província de Nampula. Contributo para o Desenvolvimento de Estratégias de profilaxia da doença em Moçambique. Maputo: Universidade Eduardo Mondlane; 2005.

26. Guimarães ELAM, Moreira SM. Variação da incidência da raiva humana no Brasil e em Moçambique de 2001 a 2007. Revista Científica Eletrônica de Medicina Veterinária 2012;18.

27. Dzikwi AA, Ibrahim AS, Umoh JU. Knowledge, attitude and practice about rabies among children receiving formal and informal education in Samaru, Zaria, Nigeria. Global journal of health science 2012 Aug 9;4(5):132-9.

28. Lembo T, Hampson K, Kaare MT, Ernest E, Knobel D, Kazwala RR, et al. The feasibility of canine rabies elimination in Africa: dispelling doubts with data. PLoS Negl Trop Dis 2010;4(2):e626.

29. Tamirat K, Alemayehu L, Mulualem T. Community Perception towards Traditional Healers and Health Centers on Management of Dog Bites and its Relation with Veterinary Public Health Activities. J Vet Sci Ani Husb 2016;4(2):202.

30. Tschopp R, Bekele S, Aseffa A. Dog Demography, Animal Bite Management and Rabies Knowledge-Attitude and Practices in the Awash Basin, Eastern Ethiopia. PLoS Negl Trop Dis 2016 Feb 22;10(2):e0004471.

31. Ghosh S, Chowdhury S, Haider N, Bhowmik RK, Rana M, Prue Marma AS, et al. Awareness of rabies and response to dog bites in a Bangladesh community. Vet Med Sci 2016 Mar 11;2(3):161-9.

32. Salomao C, Nacima A, Cuamba L, Gujral L, Amiel O, Baltazar C, et al. Epidemiology, clinical features and risk factors for human rabies and animal bites during an outbreak of rabies in Maputo and Matola cities, Mozambique, 2014: Implications for public health interventions for rabies control. PLoS Negl Trop Dis 2017 Jul;11(7):e0005787.

33. WHO. Rabies. World Health Organization 2019.

34. Lushasi K, Steenson R, Bernard J, Changalucha JJ, Govella NJ, Haydon DT, et al. One Health in practice: using Integrated Bite Case Management to increase detection of rabid animals in Tanzania. Frontiers in public health 2020;8:13.

35. Sambo M, Cleaveland S, Ferguson H, Lembo T, Simon C, Urassa H, et al. The burden of rabies in Tanzania and its impact on local communities. PLoS Negl Trop Dis 2013;7(11):e2510.

36. Hudson EG, Dhand N, Dürr S, Ward MP. A Survey of Dog Owners in Remote Northern Australian Indigenous Communities to Inform Rabies Incursion Planning. PLoS Negl Trop Dis 2016 Apr 26;10(4):e0004649.

37. Sambo M, Lembo T, Cleaveland S, Ferguson HM, Sikana L, Simon C, et al. Knowledge, attitudes and practices (KAP) about rabies prevention and control: a community survey in Tanzania. PLoS Negl Trop Dis 2014;8(12):e3310.

38. Schwitters A, Lederer P, Zilversmit L, Gudo PS, Ramiro I, Cumba L, et al. Barriers to health care in rural Mozambique: a rapid ethnographic assessment of planned mobile health clinics for ART. Glob Health Sci Pract 2015 Mar;3(1):109-16.

39. INE. Anuário Estatístico 2016 - Moçambique (Statistical Yearbook 2016 – Mozambique). Instituto Nacional de Estatísticas (National Statistics Institute) 2017;9-108.

40. INE. Anuário Estatístico 2018 - Província de Gaza. Instituto Nacional de Estatísticas (National Statistics Institute) 2018;1-56.

41. Cambule AH, Smaling EMA. Assessment of soil organic carbon stocks in the Limpolo National Park: from legacy data to digital soil mapping University of Twente; 2013.

42. Stalmans M, Gertenbach WPD, Carvalho-Serfontein F. Plant communities and landscapes of the Parque Nacional do Limpopo, Moçambique. Koedoe 2004;47(2):61-81.

43. Massé F. Wildlife, cattle, and people in the Limpopo National Park: A more-than-human political ecology of conservation-induced displacement and resettlement. Toronto, Ontario: York University; 2013.

44. Le Bel S, Murwira A, Mukamuri B, La Grange M, Czudek R, Taylor R. Human wildlife conflicts in southern Africa: riding the whirl wind in Mozambique and in Zimbabwe. In: J.López-Pujol, editor. The Importance of Biological Interactions in the Study of Biodiversity.Rijeka, Croatia: INTECH Open Access Publisher; 2011. p. 283-322.

45. Witter R. Elephant-induced displacement and the power of choice: moral narratives about resettlement in Mozambique's Limpopo National Park. Conservation and Society 2013;11(4):406.

46. Dohoo IR, Martin W, Stryhn HE. Veterinary epidemiologic research. Charlottetown: AVC Inc.; 2003.

47. Simone E. Evaluation of the level of protection against Rabies in dogs and the awareness of dog owners regarding the disease in Manica Province, Mozambique [Preventive Veterinary Medicine]. Maputo. Mozambique: Faculty of Veterinary. University Eduardo Mondlane; 2016.

48. Parque Nacional do Limpopo. Composição de agregados familiares de algumas zonas do Parque Nacional do Limpopo no âmbito do reassentamento (2014-2015). Ministério da Terra, Ambiente e Desenvolvimento Comunitário 2017.

49. Thrusfield M. Veterinary Epidemiology. 3 ed. Oxford, UK: Blackwell Publishing company; 2007.

50. da Costa LJC, Fernandes MEB. Rabies: knowledge and practices regarding rabies in rural communities of the Brazilian Amazon Basin. PLoS Negl Trop Dis 2016;10(2):e0004474.

51. Kapoor P, Baig VN, Kacker S, Sharma M, Sharma M. A cross-sectional study of knowledge regarding rabies among attendees of anti-rabies clinic of a teaching hospital, Jaipur. Journal of family medicine and primary care 2019;8(1):194.

52. Kishore S, Singh R, Ravi SK. Knowledge, attitude and practice assessment in health workers regarding rabies disease and its prevention in district Dehradun of Uttarakhand. Indian Journal of Community Health 2015;27(3):381-5.

53. Kisiel LM, Jones-Bitton A, Sargeant JM, Coe JB, Flockhart DT, Palomar AR, et al. Owned dog ecology and demography in Villa de Tezontepec, Hidalgo, Mexico. Preventive veterinary medicine 2016;135:37-46.

54. Morters MK, McKinley TJ, Restif O, Conlan AJ, Cleaveland S, Hampson K, et al. The demography of free-roaming dog populations and applications to disease and population control. J Appl Ecol 2014 Aug;51(4):1096-106.

55. National Livestock Development Directorate. Mozambique relevant country data. Ministry of Agriculture and Rural Development; 2019. Report No.: In Timeframe (e.g. annual reports, total over the past 2 years).

56. Reece JF, Chawla SK. Control of rabies in Jaipur, India, by the sterilisation and vaccination of neighbourhood dogs. Veterinary Record 2006;159(12):379-83.

57. Kitala P, McDermott J, Kyule M, Gathuma J, Perry B, Wandeler A. Dog ecology and demography information to support the planning of rabies control in Machakos District, Kenya. Acta tropica 2001;78(3):217-30.

58. Muriuki BJ. Knowledge, Attitude and Practices on Rabies in Kisumu and Siaya Counties, Kenya. MSc Thesis University of Nairobi 2016;52-5.

59. Ratsitorahina M, Rasambainarivo JH, Raharimanana S, Rakotonandrasana H, Andriamiarisoa MP, Rakalomanana FA, et al. Dog ecology and demography in Antananarivo, 2007. BMC Veterinary Research 2009;5(1):21.

60. Mauti S, Traoré A, Sery A, Bryssinckx W, Hattendorf J, Zinsstag J. First study on domestic dog ecology, demographic structure and dynamics in Bamako, Mali. Preventive veterinary medicine 2017;146:44-51.

61. Digafe RT, Kifelew LG, Mechesso AF. Knowledge, attitudes and practices towards rabies: questionnaire survey in rural household heads of Gondar Zuria District, Ethiopia. BMC Res Notes 2015;8(1):400.

62. Kabeta T, Deresa B, Tigre W, Ward MP, Mor SM. Knowledge, Attitudes and Practices of Animal Bite Victims Attending an Anti-rabies Health Center in Jimma Town, Ethiopia. PLoS Negl Trop Dis 2015 Jun 26;9(6):e0003867.

63. Hampson K, Dobson A, Kaare M, Dushoff J, Magoto M, Sindoya E, et al. Rabies Exposures, Post-Exposure Prophylaxis and Deaths in a Region of Endemic Canine Rabies. PLoS Negl Trop Dis 2008 Nov 25;2(11):e339.

64. Alie A, Assefa A, Derso S, Ayele B. Assessment of knowledge, attitude and practice on rabies in and around Debretabor, South Gondar, Northwest Ethiopia. Int J Basic Appl Virol 2015;4(1):28-34.

65. Mucheru GM, Kikuvi GM, Amwayi SA. Knowledge and practices towards rabies and determinants of dog rabies vaccination in households: a cross sectional study in an area with high dog bite incidents in Kakamega County, Kenya, 2013. The Pan African medical journal 2014;19.

66. Matibag GC, Kamigaki T, Kumarasiri PV, Wijewardana TG, Kalupahana AW, Dissanayake DR, et al. Knowledge, attitudes, and practices survey of rabies in a community in Sri Lanka. Environ Health Prev Med 2007 Mar;12(2):84-9.

67. Ntampaka P, Nyaga PN, Niragire F, Gathumbi JK, Tukei M. Knowledge, attitudes and practices regarding rabies and its control among dog owners in Kigali city, Rwanda. PloS one 2019;14(8).

68. Cleaveland S, Kaare M, Tiringa P, Mlengeya T, Barrat J. A dog rabies vaccination campaign in rural Africa: impact on the incidence of dog rabies and human dog-bite injuries. Vaccine 2003 May 16;21(17ΓÇô18):1965-73.

69. Mbilo C+, Kabongo JB, Pyana PP, Nlonda L+, Nzita RW, Luntadila B, et al. Dog Ecology, Bite Incidence, and Disease Awareness: A Cross-Sectional Survey among a Rabies-Affected Community in the Democratic Republic of the Congo. Vaccines (Basel) 2019 Aug 26;7(3):98.

70. Awuni B, Tarkang E, Manu E, Amu H, Ayanore MA, Aku FY, et al. Dog OwnersΓÇÖ Knowledge about Rabies and Other Factors That Influence Canine Anti-Rabies Vaccination in the Upper East Region of Ghana. Tropical medicine and infectious disease 2019;4(3):115.

71. Ameh VO, Dzikwi AA, Umoh JU. Assessment of knowledge, attitude and practice of dog owners to canine rabies in Wukari Metropolis, Taraba State Nigeria. Global journal of health science 2014;6(5):226.

72. Bouaddi K, Bitar A, Bouslikhane M, Ferssiwi A, Fitani A, Mshelbwala PP. Knowledge, Attitudes, and Practices Regarding Rabies in El Jadida Region, Morocco. Veterinary sciences 2020;7(1):29.

73. Mbaipago N, Mindekem R, Oussiguere A, Moyengar R, Naïssengar K, Madjadinan A, et al. Rabies knowledge and practices among human and veterinary health workers in Chad. Acta tropica 2020;202:105180.

74. Bourhy H, Troupin C+, Faye O, Meslin FX, Abela-Ridder B, Kraehenbuhl JP. Customized online and onsite training for rabies-control officers. Bulletin of the world Health Organization 2015;93:503-6.

75. Bhalla S, Mehta JP, Singh A. Knowledge and practice among general practitioners of Jamnagar city regarding animal bite. Indian Journal of Community Medicine 2005;30(3):94.

76. Shah SF, Jawed M, Nooruddin S, Afzal S, Sajid F, Majeed S, et al. Knowledge and practices among the general practitioners of Karachi regarding dog bite management. JPMA The Journal of the Pakistan Medical Association 2009;59(12):861-4.

77. Amrita S, Sudip B, Chintan B, Aniruddha G, Naresh M, Dipesh P. An assessment of knowledge of prevention and management of Rabies in interns and final year students of Shri MP Shah Government Medical College, Jamnagar, Gujarat. Journal of Research in Medical and Dental Science 2013;1(2):62.

78. Mallewa M, Fooks AR, Banda D, Chikungwa P, Mankhambo L, Molyneux E, et al. Rabies encephalitis in malaria-endemic area, Malawi, Africa. Emerg Infect Dis 2007 Jan;13(1):136-9.

79. WHO. Rabies vaccines: WHO position paper, April 2018 Recommendations. Vaccine 2018;36(37):5500-3.

80. Malhotra V, Balgir RS, Watts A, Kaur S, Nirwan PS, Cheema R. Awareness regarding animal bite management among resident doctors of tertiary care institute of Northern India. Medical Journal of Dr DY Patil University 2017;10(4):359.

81. Malhotra V, Singh A, Balgir RS. Knowledge and practices about animal bite management among government doctors posted at primary health-care settings of district Patiala in Punjab. Journal of Current Research in Scientific Medicine 2018;4(1):47.

82. Niang K, Tine JA, Ndao AB, Diongue FB, Diallo AI, Faye A, et al. Knowledge and Attitudes of Health Care Providers and the Population about Rabies in Sokone Health District, Senegal. Open Journal of Preventive Medicine 2020;10(04):63.

83. Tiwari HK, Vanak AT, O'Dea M, Robertson ID. Knowledge, attitudes and practices towards dog-bite related rabies in para-medical staff at rural primary health centres in Baramati, western India. PloS one 2018;13(11):e0207025.

84. Barreto A, Matos CS, Gujral L. MANUAL DA RAIVA - PROFILAXIA E VIGILÂNCIA EPIDEMIOLÓGICA. 1st ed. Maputo: Imagem Global; 2004.

85. Milgroom J, Spierenburg M. Induced volition: Resettlement from the Limpopo National Park, Mozambique. Journal of Contemporary African Studies 2008;26(4):435-48.

86. Governo do Distrito de Massingir. Plano Local de Adaptação às Mudanças Climáticas. https://cgcmc gov mz/attachments/article/191/PLA%20MASSINGIR%20%20final%20%20(1) pdf 2015;1-52.

Supporting information

S1. Table S1. Frequency of dog vaccination per village and reasons for non-vaccinating against rabies

S2. Table S2. Knowledge of rabies, its transmission, clinical signs, and disease outcome among Limpopo National Park's households

S3. Table S3. Knowledge of rabies prevention among households, Limpopo National Park, Massingir.

S4. Table S4. Knowledge of rabies causative agent, main animal source of rabies in Mozambique, mode of transmissions, clinical features, incubation and concept of rabies categories of exposure among Health practitioners, Massingir district

Figure 2. Location of all eight Health Facilities of Massingir Districtk health centresg the study area (study villages are indicated in the rectangles).

Click here to access/download;Figure;Fig2.tif

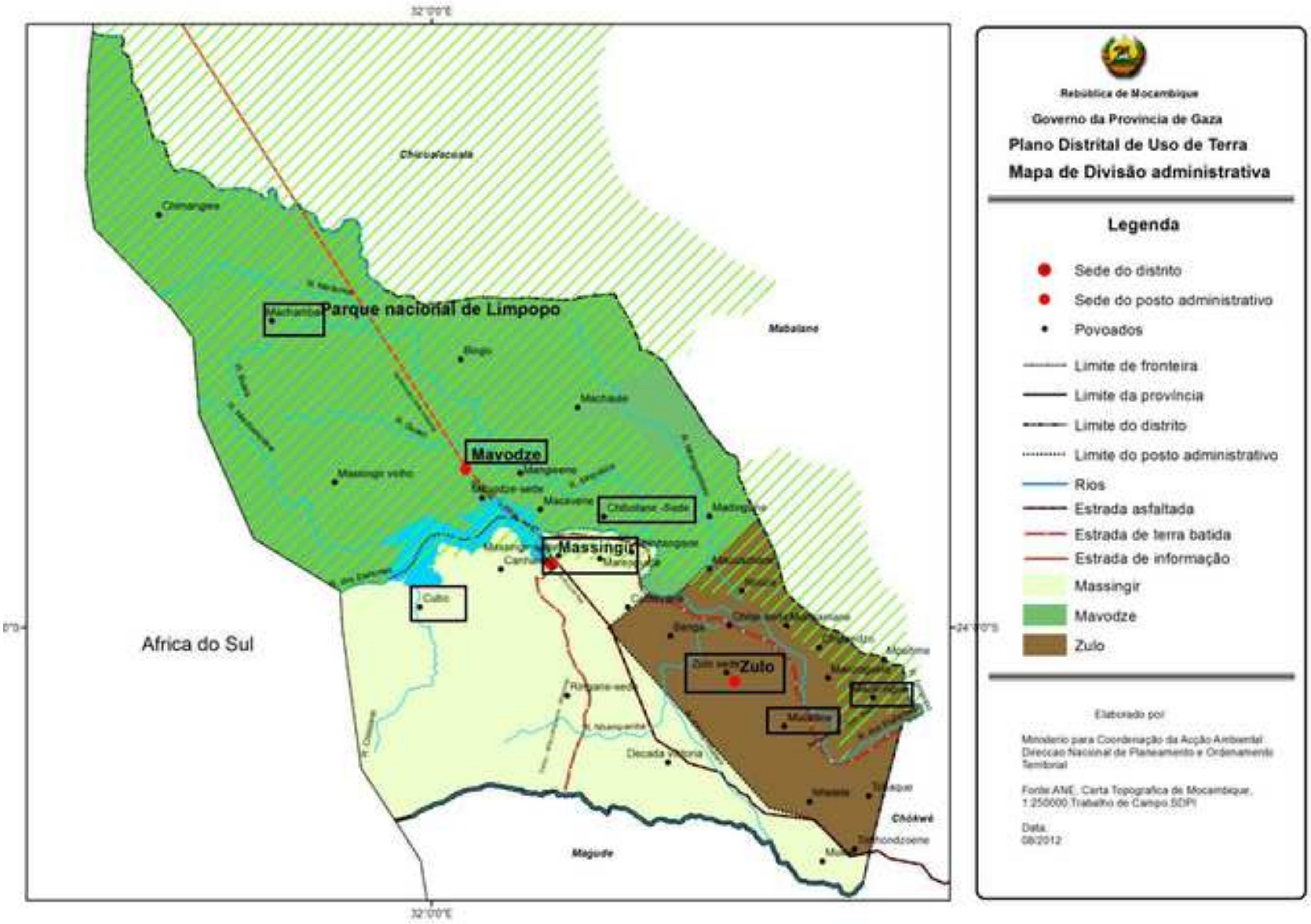

Figure 3. Frequency of households' responses regarding animals affected by rabies

Click here to access/download;Figure;Fig3.tif

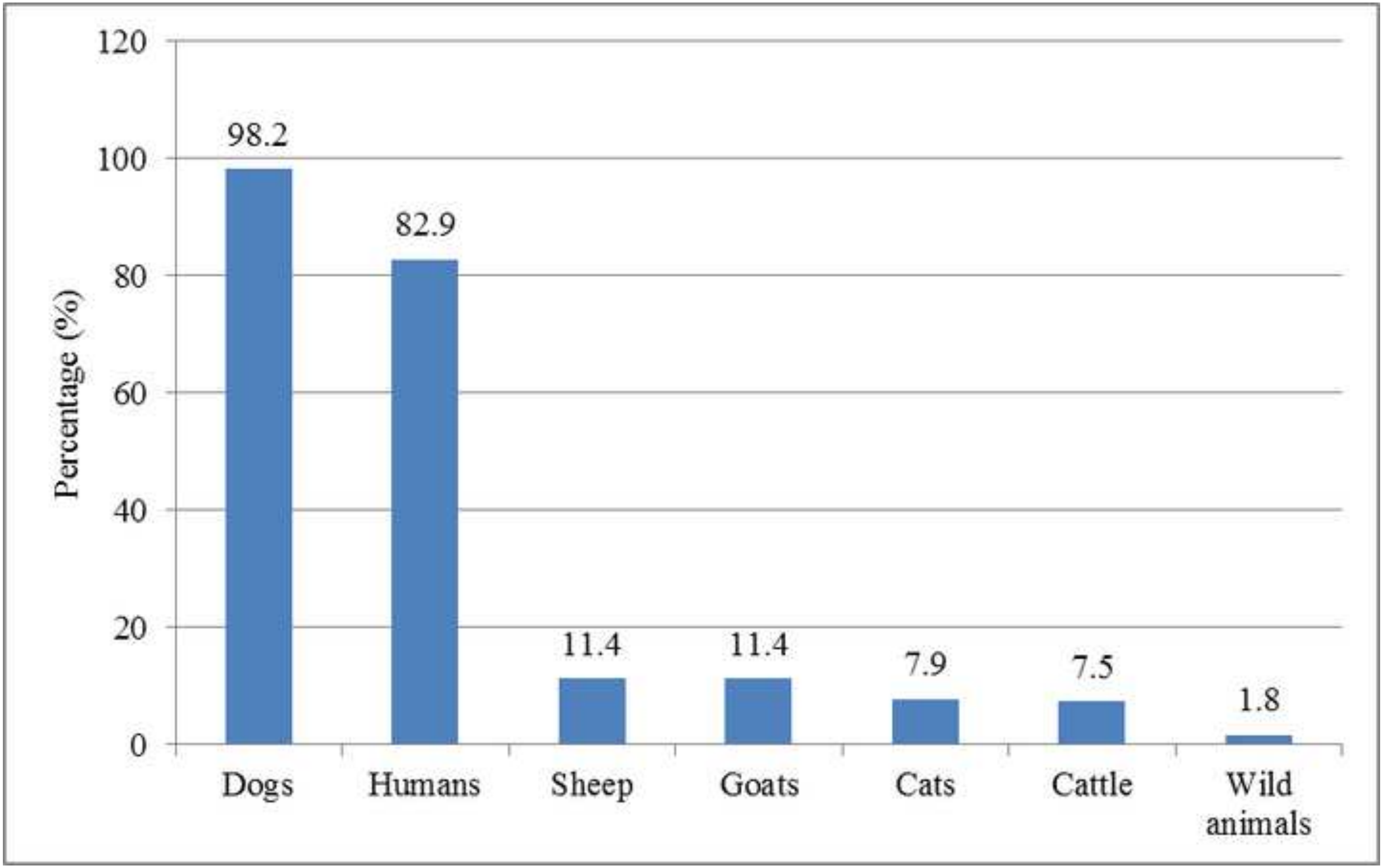

Figure 4. Frequency of households' responses regarding symptoms observed in rabies cases

Click here to access/download;Figure;Fig4.tif

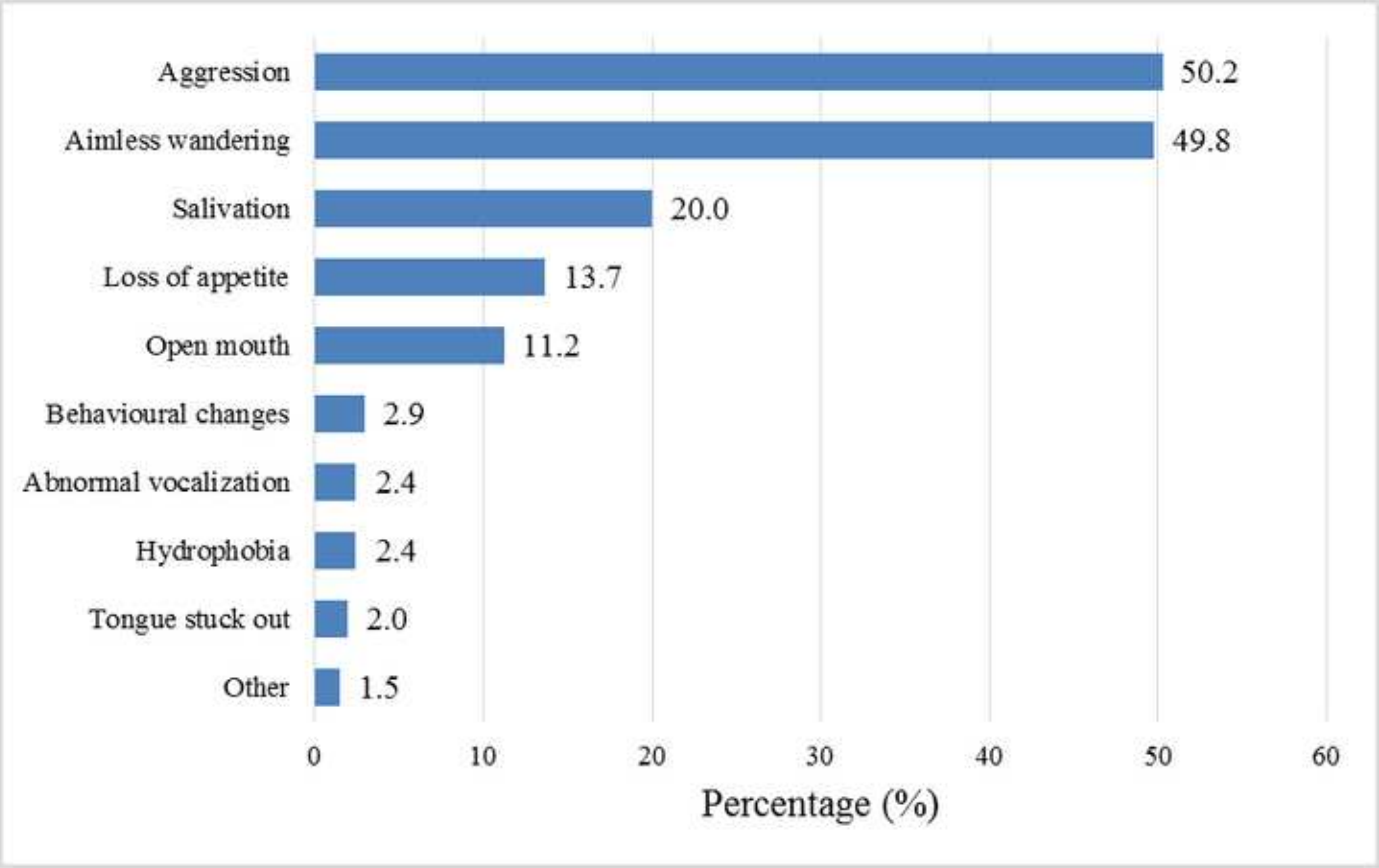

Figure 5. Professional role of Health practitioner respondents of Massingir district

Click here to access/download;Figure;Fig5.tif

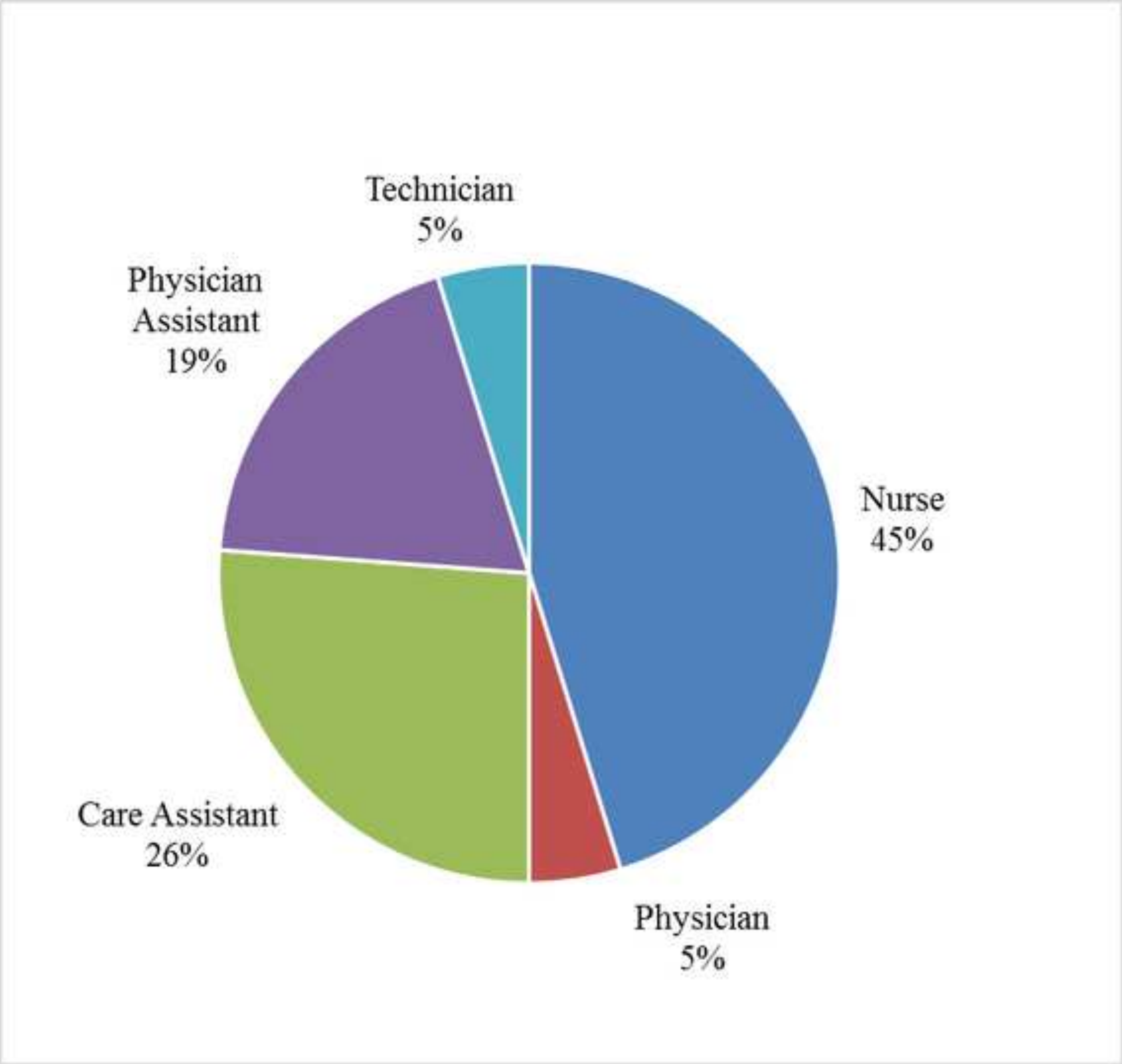

Figure 6. Health practitioners' practices regarding bite wound practices (n=42) Click here to access/download;Figure;Fig6.tif

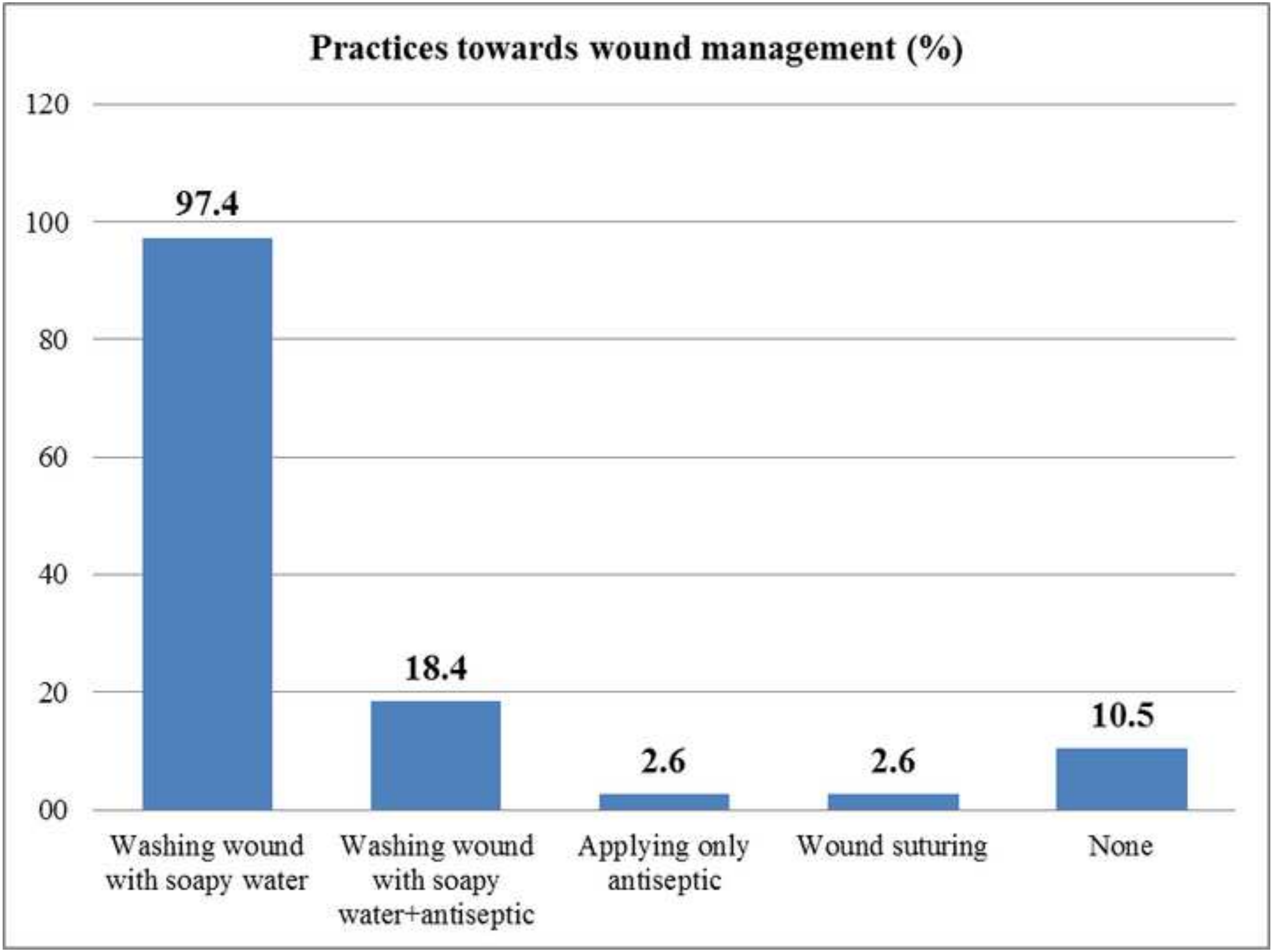

Figure 1. Limpopo National Park Map showing the study area (villages included in the study are indicated by rectangles).

Click here to access/download;Figure;Fig1.tif

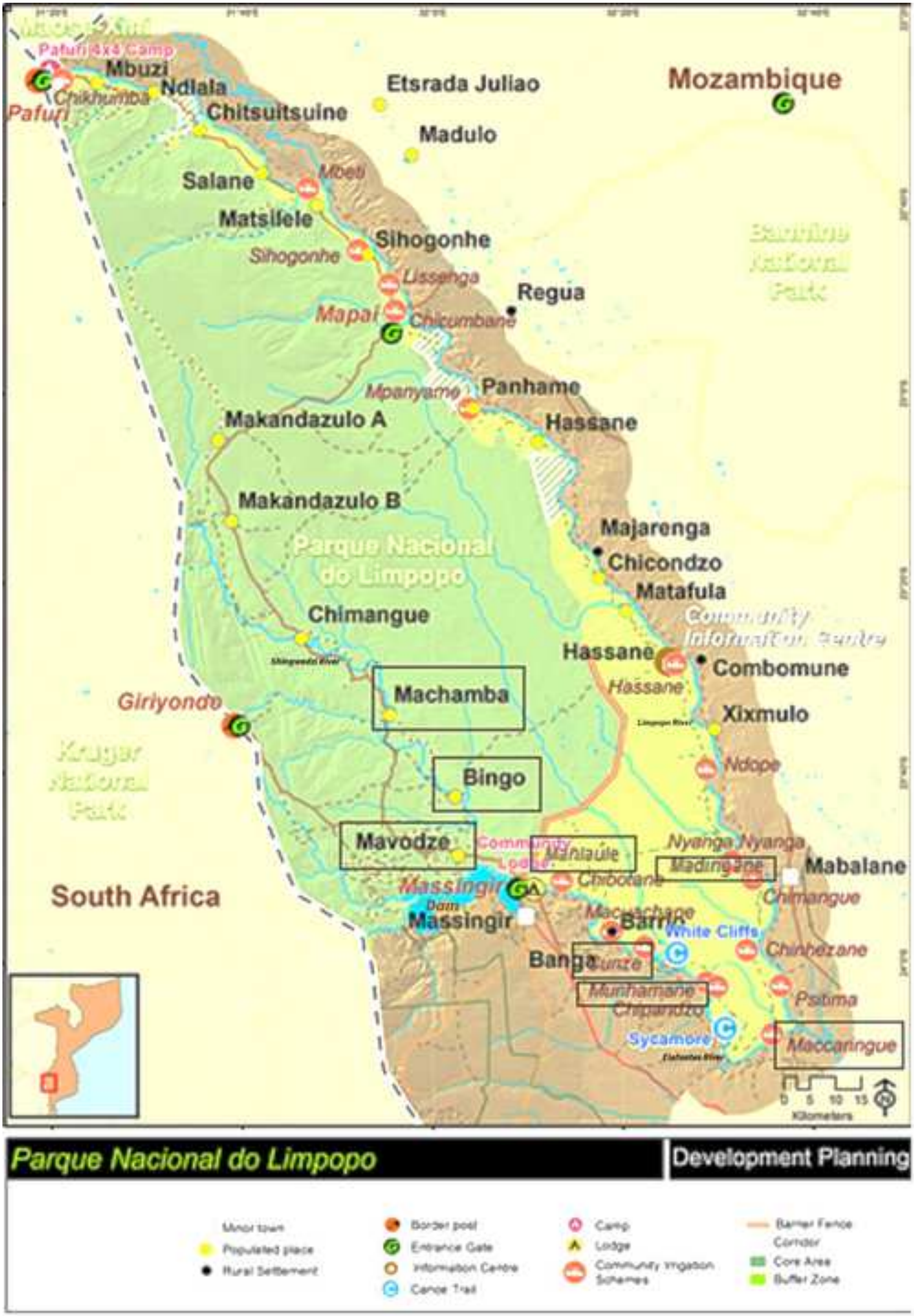

Click here to access/download

Supporting Information - Compressed/ZIP File Archive

Suplemmentary information S1 S2 S3 and S4.zip

2nd June 2021

Daniel Leo Horton,

Associate Editor

PLOS Neglected Tropical Diseases

Dear Prof. Horton

Subject: **PNTD-D-20-01733**

The authors would like to thank you for your correspondence regarding the second review of our manuscript "Knowledge, attitudes, practices (KAPs) and control of rabies among community households and health practitioners at the human-wildlife interface in Limpopo National Park, Massingir District, Mozambique" by Milton Mapatse, Claude Sabeta, José Fafetine, Darrell Abernethy. Responses to the reviewers' comments and changes made to the manuscript are presented below. The reviewers' comments are presented in italics, the author's response in bold text. Pages numbers are based on the revised (with track changes) version of the manuscript.

Major revisions

Methods

Reviewer #1:

This study assessed the knowledge, attitudes and practices towards rabies among selected households and health practitioners in Limpopo National Park, Massingir district, Mozambique. Need to improve on this objective terms like control are not captured in the abstract at least reconstruct the objective in the abstract.

Response: The objective has been improved both in the abstract (Page 1) and along the text on page 6.

The number of household + medical setting samples captured in this study are a small size, why such a small sample , clearly explain the type of sampling was it purposive or convenient sampling. And justify for the small sample size(households + medical settings) obtained in this study .

Response: For the Limpopo National Park scenario, the sample size is representative of the study area or population. In the methodology, we have improved the explanation of how the sample size calculation was done (Page 8). Purposively we selected all those Households and Health practitioners who met the requirements that allowed us to achieve our objectives. Still, it was always a challenge to include some households that met the criteria to be part of the study.

For more details of the difficulties in recruiting and working in a tense political climate in Limpopo National Park villages as observed in this study, please read the section on the study limitations (Page 35) including the following papers: Milgroom J, Spierenburg M. Induced volition: Resettlement from the Limpopo National Park, Mozambique. Journal of Contemporary African Studies 2008;26(4):435-48 and Milgroom, Jessica, Cees Leeuwis, and Janice Jiggins. "Limpopo case: the role of research in conflict over natural resources; informing resettlement negotiations in Limpopo National Park, Mozambique." Knowledge in action. Wageningen Academic Publishers, Wageningen, 2011. 247-276.

Regarding Health professionals, as it is a remote area, human resources are limited (only two doctors and physician's assistant for the entire district). Furthermore, the health network, not only at district, but also at national level, is still below ideal and desirable in terms of human, material and infrastructural resources (Page 9).

Do you think these findings can be used by the authority to implement rabies control programs in River LNP if so explain.

Response: Thank you for this important question. It really was not clearly reflected in the manuscript on how the findings of study could help the authorities in implementing rabies control programmes in Limpopo National Park. Therefore, we have decided to elaborate more on the subject in the conclusions and recommendations chapter (Page 34 and 35).

Being a national park i expected that may be we have other rabies virus transmitters in the wild that have attacked humans and animals apart from domestic dogs.

Response: Human-Wildlife conflict does occur in LNP, but during the period covered by this study, there were no reports or confirmed cases of wild animals attacking humans. In the supplementary table (Table S2) some interviewees claimed to have seen besides dogs, also cats, cattle, sheep and goats with signs of rabies. In the last 12 years prior to the study, only cases of people being bitten by dogs were reported.

I have looked through the document, the results and conclusions are clearly presented, my concern as scientist before you set out to conduct this study. 1) what was your research question or hypothesis that you want to prove right or wrong about rabies in Limpopo national park.

Response: The Limpopo National Park (LNP) is a unique human, domestic animal and wildlife interface region characterised by a complex epidemiological relationship involving a diversity of pathogens, including rabies virus. Compared to what is observed in the bordering and neighbouring Kruger National Park, rabies research in LNP is poor and challenging due to poor infrastructure,

dispersed communities and uncertainty around local commitment. The lack of rabies information in communities in the park has prompted the following research question: What do the population and health professionals in the LNP know about rabies and how can this baseline knowledge be used to support the design and implementation of disease control strategies in the human population, domestic animals and wildlife in the region?

Reviewer #2:

1. It is difficult to read. One can hardly grasp the idea of the paper because of so many typo and clerical errors.

Response: Thank you for the comments. The authors have thoroughly revised the manuscript and to address typo and clerical errors, it was heavily edited by a native English speaker.

2. There is inconsistency in the presentation of numerical data in the narrative.

Response: We have standardised the presentation of numerical data throughout the manuscript by prioritising the presentation of the numerical data in percentage form.

3. The Tables are not of sufficient quality to be acceptable to an average reader.

Response: The authors have improved the presentation of the information in the Tables (Table 1, Table 2 and Table 3 and Table 6) to facilitate the understanding and interpretation of the same by readers.

4. There are issues in the Discussion section that are emphasized to be part of the study but are not shown in the Results section, e.g. sex of the dogs.

Response: Thank you very much for the comment. The corresponding paragraph related to sex of the dogs has been deleted (Page 27).

<u>Conclusions, Summary and General Comments</u>

Reviewer #1: Yes the conclusions are supported by the small set of data presented regarding the smaller number of medical professions that is fine. But i dont think the households in the LNP were limited as well. Clear explanation for this need to be presented. The authors have discussed the topic well and have shown how the data collected can be useful if certain situations are improved in the area.

Response: The authors are grateful for the comments left by Reviewer #1. We believe that in the study limitation section (Page 34) we have elucidated the reasons for the limited participation of households in this study. We hope that we have addressed and taken on board your suggestions and recommendations.

Minor revisions <u>for the comments found in the manuscript</u>

Comment 1. Line 166-186: There was too much discussion on the calculation of the sample size but missed the two most important things readers would be interested in—the margin of error and confidence interval. Suggest to remove the explanation on the calculation. Report instead the calculated ME and 95%CI of the sample. The shorter and simpler the explanation is, the better, and is much easier to understand.

Response: We have improved the description of how the sample size was established and the margin of error and confidence interval were elucidated (Page 8).

Comment 2.

Line 239-240: 95% CI; P value <.05

Response: The correction has been made in the manuscript (Page 11).

Comment 3. Table 1: Suggested arrange of the variables

Response: Table 1, Table 4 have been rearranged according to your suggestion (Page 11, 12 and 18).

Comment 4. Line 243-250: Just place the percentages. Not necessary to write down the numerators and denominators in the narrative. They are already mentioned in the Tables. Do this all throughout the paper.

Response: The authors are grateful for the suggestion and have proceeded as recommended throughout the manuscript.

Comment 5 . Line 259:...single dog (22.2%)…multiple dogs (16.5%) (P=.605). (No need to place 0 before .605). Follow this pattern throughout.

Comment 6. Line 280: Same as above.

Response: Your suggestion has been taken into account throughout the paper. We have followed the pattern recommended above.

Comment 7. Line 286: The frequency of dog vaccination per village and reasons for non-vaccination are shown on Table 3.

Response: The sentence has been updated in the text (Page 14).

Comment 8. Line 324: Only 1.6% of respondents….

Response: The text has been corrected (Page 16).

Comment 9. Line 332: A high proportion (93.0%) of respondents said they would euthanize their dog if it were rabid.

Response: The text has been corrected (Page 16).

Comment 10. Line 337-338: How come you say the sample having has good knowledge and practices on rabies with only 18.9% and 13.3%? What is the basis for saying good?

Response: We have adopted a scoring system to rank the responses as such (see the topic on Data entry and analysis) (Pages 10 and 11).

Comment 11. Line 358: Knowledge, attitudes and practices on rabies of the study participants in LNP based on location, gender, education, occupation

Response: The caption of Table 3 has been updated as suggested (Page 18).

Comment 12. Line 370: The knowledge…and incubation period…were elucidated…

Response: The text has been edited (Page 20).

Comment 13. Line 379-380: …believed they knew the symptoms…and most of the health practitioners knew the disease was fatal.

Response: The text was updated (Page 20).

Comment 14. Line 407: …were aware of the advice…

Response: The text has been updated (Page 22).

Comment 15. Line 440: All respondents? But there is only one respondent.

Response: We agreed with your comment and we have reworded the text (Page 24).

Comment 16. Line 442: There were more male respondents who had good knowledge…

Response: We have corrected the sentence (Page 24).

Comment 17. Line 461: Why emphasize on the sex of the dog when it was not part of the questionnaire? What data in your study supports this claim about male dogs? I think it is better to delete that statement if your study could not prove it objectively.

Response: Thank you very much for the comment. The statement has been deleted (Page 27).

Click here to access/download
Revised Article with Changes Highlighted
PNTD-D-20-01733 KAPs Rabies II versionTrack changes.docx
