# Supplementary material for: Knowledge, attitudes, practices (KAP) and control of rabies among community households and health practitioners at the human-wildlife interface in Limpopo National Park, Massingir District, Mozambique
Source: PLoS Negl Trop Dis. 2022 Mar 7;16(3):e0010202. doi: 10.1371/journal.pntd.0010202 (PMC8929695; doi:10.1371/journal.pntd.0010202)
Supplement: Attachment — Submitted filename: KAPs Rabies households and health practictioners Mozambique GM.docx [file pntd.0010202.s004.docx]

**Knowledge, attitudes, practices (KAPs) and control of rabies among community households and health practitioners at the human-wildlife interface in Limpopo National Park, Massingir District, Mozambique**

Milton Mapatse^1^**^*^**, Claude Sabeta^2,3^, Darrell Abernethy^4,5^, José Fafetine^1,6^

^1^ Veterinary Faculty, University Eduardo Mondlane, Maputo, Mozambique

^2^ OIE Reference Laboratory, Rabies Unit, Agricultural Research Council-Onderstepoort Veterinary Institute, Pretoria, South Africa

^3^ Department of Veterinary Tropical Diseases, University of Pretoria, Pretoria, South Africa

^4^ Centre for Veterinary Wildlife Studies, University of Pretoria, Pretoria, South Africa

^5^ Aberystwyth School of Veterinary Science, Institute of Biological, Environmental and Rural Sciences, Aberystwyth University, Aberystwyth, SY 23 3EB, United Kingdom

^6^ Centre of Biotechnology (CB-UEM), Maputo, Mozambique

***** Corresponding author

E-mail: [miltonkitovet@gmail.com](mailto:miltonkitovet@gmail.com)

**Abstract**

**Background**

Rabies is a deadly viral and zoonotic disease that kills more than 26,000 people each year in Africa. In Mozambique, poverty and inadequate surveillance exacerbate the situation, resulting in ineffective rabies control and many dog cases and human deaths. This study assessed the knowledge, attitudes and practices towards rabies among households and health practitioners in Limpopo National Park, Massingir district in order to contribute to improve strategies for intervention including awareness, educational programs and disease control.

**Methodology**

A cross-sectional study was conducted during 2016 and 2018 in households residing in eight Limpopo National Park villages and among health practitioners from eight health facilities in Massingir District. A questionnaire was administered to collect information on dog ownership and knowledge of rabies, including existence of disease, range of species affected, mode of transmission, symptoms, recommended treatment and methods of prevention. The survey included 233 households and 42 health practitioners.

**Results**

In general, 44 (18.9%) and 31 (13.3%) of the households respectively had good knowledge and practices of rabies. For health practitioners, seven (16.7%) had good knowledge, whilst 14 (33.3%) adopted adequate attitudes/practices towards the disease.

**Conclusions/significance**

Lack of community-based education and professional retraining courses were determinant factors contributing to the poor awareness of rabies. This study therefore provides an opportunity to enhance public health knowledge and reduce dog-mediated human rabies.

**Author summary**

In Mozambique, rabies has been endemic since 1908 and is maintained by the domestic dog, the principal vector responsible for the majority of human cases. Traditionally, animal rabies control has been driven by the government veterinary authorities, but with limited collaboration from other sectors such as health and environment. In the Massingir District in general, and in LNP in particular, parenteral dog rabies mass-vaccination campaigns and the provision of post-exposure prophylaxis are not practiced. Consequently, limited resources and funds for the implementation of vaccinations in the field, insufficient veterinary field staff and inefficient or non-existent disease notification procedures and inadequate training of health practitioners constraint effective rabies control in Mozambique. The awareness of the local population and health practitioners about good practices regarding management of bite wounds is crucial to minimize human deaths from rabies. The results obtained here will inform government policy on practical interventions in the control of animal and human rabies.

**Introduction**

Rabies is one of the oldest neglected infectious diseases known in medical history, and affects all warm-blooded animals, including humans. Today, rabies remains a major public and veterinary health problem in developing countries, especially in the tropical and subtropical regions of Africa and Asia [1,2], where in some areas it is considered a re-emerging disease [3,4]. Upon entry into a susceptible host, the highly neurotropic virus induces an acute, progressive and fatal encephalomyelitis mainly through bite contact with infectious saliva, contact with mucous membranes and also through scratches [5]. The causative agent responsible for more than 95% of human rabies deaths is a classical rabies virus (RABV) that belongs to the genus *Lyssavirus*, family *Rhabdoviridae* [6-8]. Members of this genus are characterized by non-segmented negative-sense RNA genomes of approximately 12 kb in size [9]. Globally, human mortality is estimated to be at least 60,000 deaths per year, 56% of these occurring in Asia and 44% in Africa, especially in rural areas where large populations of stray dogs are found [10-12]. Poverty and poor awareness of the disease are associated with increased vulnerability to rabies and are, consequently, major obstacles in preventing rabies spread, especially in rural areas [13,14]. The general understanding of the rural communities about dog behaviour, responsible pet ownership, appropriate health service-seeking attitudes following dog bites and rabies prevention are crucial to raising the general public awareness of the disease and reducing the number of human rabies deaths [15,16]. The National Strategy for the Control of Rabies in Mozambique was approved by the Council of Ministers in 2010 with a view to controlling rabies in the animal population by limiting its expansion and spread to humans. Despite this, rabies deaths have been reported every year from all provinces of the country [17,18]. In a retrospective investigation of rabies covering a 29-year period (1989 to 2017) in Mozambique, 1,001 human cases were documented [19]. According to this same study, there was an increase in human rabies deaths, ~~from 44~~ in 2010 to about 2-fold more cases annually between 2016 (n=94) and 2017 (n=89). Furthermore, in the last four decades, there has been population movement to occupy some of the conservation areas including Massingir [20]. These remote communities residing in human-wildlife interface areas, where they rely on their dogs for hunting and guarding [21,22] and also face the risk of contracting rabies either directly from rabid wild carnivores such as African wild dogs, bat-eared fox, jackals, mongoose and hyenas [17,23] or through spillover of wildlife rabies into dogs. This worrying reality about the risk of rabies is described by Osofsky et al. [24] using the LNP scenario as an example, a situation arising from the lack of control and vaccination of dogs. There is a paucity of data in Mozambique on the knowledge of, and attitudes to, rabies [25,26] and like other African countries, children under 15 years, who are the main victims are unaware of where to report incidents involving bite contact to receive proper treatment [27,28]. In these cases, the majority of contact victims seek help from traditional healers instead of visiting the nearest health centres [29,30]. Furthermore, in such settings, suspected rabid dogs are destroyed without first being quarantined [31-33]. In countries such as Tanzania, bite management centres have been set up and have improved the surveillance of rabies in dogs [34,35]. Enhancing public health awareness of rabies in the complex socio-ecological systems of protected wildlife will address the identified gaps, cultural beliefs and behaviour patterns, designing relevant public health educational campaigns, and provide baseline data for planning, implementation and evaluation of national control programmes [36,37]. These may assist in the elimination of dog-mediated human rabies in the country and on the continent. Despite the implementation of the Mozambique National Health Plan for strengthening primary health care, the shortage of health facilities, poor infrastructure and inadequate training of medical staff, particularly concerning the management of dog bites and rabies cases, remains a concern [38]. In the Massingir District, the medical doctor to population in 2018 was ~~recorded as~~ 0.1 physicians per 1000 population, which like the rest of the country (0.07 physicians/1000 population), means that healthcare services are mostly provided by technicians with elementary to intermediate skills in medical care [39,40]. It is crucial that these caregivers have the correct level of knowledge on the management of animal bites and prevention of rabies. Hence, the objectives of this study were to determine the degree of knowledge, attitudes, practices and awareness towards dog bites and rabies among households and healthcare practitioners in the protected Limpopo National Park (LNP), in Massingir district.

**Materials and Methods**

**Characteristics of the study area and population**

The study was carried out in the Limpopo National Park (LNP), Mozambique, (Figure 1) for the period 2016 to 2018. The LNP is located in the west of Gaza Province and is delimited by 190 km of fenced international border with South Africa (Kruger National Park) to the west, and by both Limpopo (about 260 km) and Elefantes (about 85 km) rivers in east and south, respectively [41,42]. The LNP contains 13 villages situated along the three major rivers, namely the Limpopo River (Macaringue, Munhamane, Cunze, Chipanzo and Maconguele villages); the Shingwedzi River (Bingo, Machamba, Chimangue and Mavoze villages) and Elefantes River (Madingane, Mahlaúle, Chibotane and Macuachane villages). Machamba, Bingo and Mavoze are located inside the park, while the remaining villages are located in the Buffer Zone, an area still considered to be within the boundaries of the park (Fig 1). They include settlements, agricultural lands (crop) and 70% of the livestock of the park [43]. The Buffer Zone extends westwards from the Limpopo River for eight to ten kilometres and northwards from the Elefantes River in the area between the latter’s confluence with the Limpopo and Massingir Dam [44].

**Fig 1. Limpopo National Park Map showing the study area (study villages are indicated in the rectangles).**

The human population of LNP was estimated to be approximately 27,000 in 2001 [45] with most inhabitants concentrated in areas with arable alluvial soils[44]. A 2015 census of animals by the District Services of Economic Activities of Massingir, revealed the population of domestic animals as 38,147 cattle; 19,338 goats; 6,838 sheep; 2,337 pigs; 1,551 dogs and 51 donkeys.

In Massingir District, there are eight health facilities, namely three inside the park, two outside the park and three in the Buffer Zone (Fig 2).

**Fig 2. Location of all eight Health Facilities of Massingir District (Courtesy of District Services of Economic Activities of Massingir).**

**Study design and sampling framework**

**Household (HHs) Survey**

A cross-sectional study was undertaken among 233 households selected within eight of the 13 LNP villages. The villages were selected by stratified multi-stage random sampling. A sampling frame was constructed of all villages and the “RAND” function in Microsoft Excel 2010 used to generate random entries. The selected villages were Machamba, Bingo and Mavoze (inside the park) and Macaringue, Mahlaúle, Madingane, Cunze and Munhamane (in the buffer zone) each representing the three main LNP riverine domains (Fig 1). A random sample scheme was used to identify the participating households, and the coverage of all village circuits was ensured until the intended number of respondents was reached.

**Sample size**

The sample size was calculated using the formula given by Dohoo et al. [46] taking into account the only two surveys conducted in the country. A weighted measure (87%) of respondents having heard of rabies based on two community-based cross-sectional studies of rabies’ awareness in central and northern Mozambique was used to calculate the target sample size with 95% confidence and 5% margin of error [25,47].

n=Z^2^ _*_ (*pq*/L^2^)

Where: n = required sample size; Z=1.96 is the value required for confidence of 95%; p = a prior estimate of the proportion (0.87); q = 1-p the complementary of prior estimate; L = Margin of error (0.05).

With 1968 households in the study area [48], the sample size was estimated to be 174, but it was extended to 233 to include another 59 households who later and voluntarily agreed to participate in the study.

**Sample selection**

This cross-sectional study included as the first eligibility criterion, respondents’ willingness, oral consent to participate and availability at the time of the visit to take part in the same study. The second eligibility criterion was those representatives of households aged 18 or over.

**Questionnaire preparation, recruitment and interviews**

Pre-survey visits were undertaken to obtain consent from the village leaders and to request their assistance in encouraging their communities to participate in the study. The selected participants were informed of the study objectives prior to completing the questionnaire. Thereafter, face-to-face interviews were conducted in Changana, the local language spoken in the region of Massingir for appropriateness and ease among study participants, but answers were recorded in Portuguese.

**Health practitioner (HPs) survey**

A cross-sectional study was carried out in all eight health facilities, in Massingir District, Gaza Province (2016 to 2018) and among all 42 personnel (doctors, nurses and caregivers) who worked there. Participants were purposively selected [49] among those who deal directly with patients on a daily basis (clinical, nursing and laboratory personnel). The investigator personally visited the HPs at their workplaces, explained the purpose of the study, assured them of confidentiality with regard to any information they might provide and obtained their oral consent. The medical personnel then completed the questionnaire during the investigator’s visit.

The study was approved by the Biotechnology Centre scientific board (University Eduardo Mondlane, Mozambique) (21.10.16) and by Animal Ethics Committee of University of Pretoria (V133-16).

**Data entry and analysis**

Two databases were created in Epi-Info, version 7 (CDC, USA) for the two study populations. Data were exported and analysed using SPSS Statistics for Windows, version 18.0 (SPSS Inc., Chicago, Ill., USA). The analysis focused on descriptive statistics to calculate the frequency, proportions, medians and means of the answers regarding both target groups (households and health practitioners).

To assess the dimension of responses (knowledge, attitudes and practices), all potential factors were grouped and scored according to the model proposed elsewhere [37,50-52], with some modifications. These proposed model scoring is according to the completeness and accuracy of respondents’ answers as well as the nature of the question.

Depending on the type of question, scores ranged from 0 to 3. For example, for questions on households’ knowledge assessment, the maximum overall score stipulated was 13, while it was 10 and 8 respectively, for attitudes and practices. Knowledge scores were categorized into “Poor” (0-6) and “Good” (7-13); attitudes into “Negative” (0-5) and “Positive” (6-10) and practice scores into “Poor” (0-3) and “Good” (4-8).

For health practitioners, the maximum overall score was 27 for “knowledge” and five for attitudes/practices. Knowledge scores regarding health practitioners were categorized into “Poor” (0-13) and “Good” (14-27); attitudes/practices into “Inadequate” (0-2) and “Adequate” (3-5).

The assessment of associations of knowledge, attitudes and practices was carried out according to some demographic characteristics, which for the case of households included village, sex and educational level, while for health practitioners, privileges were given to location of the health post/centre, years of service and professional qualifications. For the evaluation of such associations, the chi-square test was used and p<0.05 were considered as statistically significant.

**Results**

**Households (HHs) characteristics and dog ownership**

Of the 233 respondents the majority were male (63.1%). Most of the interviewees were aged between 50 and 60 years, comprising 40.3% of study population. Over 60% of respondents (n=141) had at least attended primary schooling, 193 were farmers (82.8%) and only 21 (9.0%) were unemployed. Among the respondents, 208 (89.3%) households across all villages owned at least one dog. For 64 households with a single dog, there was a significant difference in the proportion of those with male dogs compared to female (73.4% versus 26.4% respectively; χ^2^ test; p<0.05). This was also observed in households with multiple dogs (n = 144 households; number of dogs 2 – 28), where the mean number of male to female dogs was 2.6 versus 1.5 respectively (~~two sample t-test;~~ p<0.05). Only 42 (20.2%) households had their dogs sterilized. For those households with dogs, the most frequent reason for keeping one was for protecting crop fields against monkeys (n=143; 68.8%) while the least popular reason was for a pet (n=2; 1.0%) (Fig 3).

|  | Percentage (%) |
| --- | --- |
| Protect crop fields against monkeys | 68.8 |
| Security | 21.6 |
| Herding | 14.4 |
| Hunting | 4.8 |
| As a pet | 1 |

**Fig 3. Frequency of households’ responses regarding the reasons to keep dogs**

**Dog confinement**

Of the households with dogs, 167 (80.3%) never confined them while in the remaining seven (3.4%) restricted them to a yard or enclosure and 34 (16.3%) had them tied. There was no difference in the proportion of households with a single dog that confined them compared to those with multiple dogs (22.2% versus 16.5% respectively; χ^2^ test; p=0.605).

**Dog bite experience and practices towards wound bite management**

The majority of the respondents (n=212; 91%) first sought help at the health centres for the treatment of wounds, while 11 (4.7%) consulted traditional healers. At the home level, the majority of households 151 (64.8%) said they would wash their wounds with water, while 57 (24.5%) would wash with soapy water.

Among all study respondents, 41 (17.6%) had experienced a member being bitten by a dog in the previous 12 months of which the majority (66.7%) were by the neighbour’s dog(s). Only one household reported a bite by a stray dog.

Treatment of the wound bite area was the most common response to a dog bite (68.3%; n = 28) while four (9.8%) sought traditional remedies. Five (12.2%) households sought post-exposure vaccination against rabies following a dog bite. In most cases (55.7%; n = 23), a nurse provided the treatment, while a traditional healer assisted in four cases (9.8%; n = 4). Where the respondent knew the status of the dog after the incident (n = 37), in 35% of cases, the dog had died while in the remaining cases had stayed healthy.

**Animal health care seeking behaviour including dog vaccination status**

Approximately one third (30.7%) of households owning dogs (n=208) visited an animal care health centre at least once a year. There was no statistical difference in the proportion of households who visited the animal health officer based on whether they had one or multiple dogs (29.7% versus 31.3% respectively; χ^2^ test; p=0.829). The most common reason for visiting the veterinarian was for vaccination (82.8% of visits) while suspicion of rabies or aggressive behaviour accounted for 9.3%. Concerning the vaccination status of their dogs, few (25.8%) respondents had their dogs vaccinated against rabies while, the majority (63.5%) who had not their dogs vaccinated advised this was due to the lack of rabies vaccination campaigns held in their villages. The extent of dog vaccination differed significantly among villages (p=0.001; ~~χ2=39.311; Df=14; LR =46.709~~).

**Households’ rabies awareness**

Of 233 respondents, 228 (97.9%) had heard of rabies and half of these had heard through family elders (n=118; 51.8%). When asked about the source of rabies, 208 (96.5%) respondents correctly identified dogs, while 4.8% identified cats, but significantly fewer correctly identified other species to be susceptible to virus infection (Fig 4). Twenty-three (10.1%) ~~of~~ respondents mentioned correctly a virus as the causative agent of rabies, whilst 31.1% incorrectly said the disease was of psychological origin. Almost all respondents (n=224; 98.2%) stated that dogs could be affected by rabies, but significantly fewer correctly identified other species as being susceptible to infection. More than eighty per cent (n=189; 82.9%) of households were aware of the zoonotic nature of rabies and bites from an infected animal was an important transmission route to humans correctly identified by 167 (88.4%) of respondents (Fig 4). Two hundred and five (87.9%) interviewees stated they had seen a case of rabies. In almost all cases (89.9%), this was in dogs, with 7.9% (n = 18) observed in other species, including three cases in humans, ten in livestock and none in wildlife. Aggression and aimless wandering were the most commonly reported symptoms, accounting for 50% of all observed symptoms (Fig 5). Of the study respondents, 187 (82%) were aware that rabies is a fatal disease.

|  |  |  |
| --- | --- | --- |
| Dogs | 224 | 4 |
| Humans | 189 | 22 |
| Sheep | 26 | 202 |
| Goats | 26 | 202 |
| Cats | 18 | 210 |
| Cattle | 17 | 211 |
| Wild animals | 4 | 224 |

**Fig 4. Frequency of households’ responses regarding animals affected by rabies**

|  | Percentage (%) |
| --- | --- |
| Other | 1.5 |
| Tongue stuck out | 1.95122 |
| Hydrophobia | 2.43902 |
| Abnormal vocalization | 2.43902 |
| Behavioural changes | 2.92683 |
| Open mouth | 11.2195 |
| Loss of appetite | 13.6585 |
| Salivation | 20 |
| Aimless wandering | 49.7561 |
| Aggression | 50.2439 |

**Fig 5. Frequency of households’ responses regarding symptoms observed in rabies cases**

**Knowledge and attitudes towards rabies prevention**

Concerning the prevention of rabies, 54.8% (n=125) of the respondents stated that it could be prevented, usually by parenteral dog vaccination (n=106; 84.8%), and half (50.0%) were aware that, after the onset of symptoms, rabies was inevitably fatal, while 59 (25.9%) thought it was curable through oral and injectable drugs (45.8%). ~~Few respondents, that is,~~ only two (1.6%) were aware of population control or confining dogs as a preventive measure. When asked about the practices taken in case of an animal with behavioural signs or changes resembling those of rabies, 220 (95.5%) of respondents said they would either kill or expel it from their homes or neighbourhood, while 3.5% would do nothing. Almost all respondents expressed a willingness to have their dogs registered (n=200; 95.7), to gladly participate in future rabies vaccination campaigns (n=231; 99.1%) and 222 (95.3%) to notify the health or veterinary authorities if bitten by a suspect rabid dog. A similar proportion advised they would euthanize their dogs is it was ever rabid.

**Global results regarding the level of knowledge, attitudes and practices towards rabies within households**

The overall level of knowledge, attitudes and practices towards rabies among households in LNP revealed that in general, 44 (18.9%) and 31(13.3%) of respondents had good knowledge and practices of rabies, whilst 203 (87.1% ) adopted positive attitudes towards the disease.

**Associations between variables under study within households**

~~The results in~~ Table 1 show that there were statistically significant differences (p <0.05) between knowledge of rabies and ~~independent variables such as~~ the location of villages and education. With about 30%, 91% and 26%, respondents living in the village of Mavoze were respectively the ones who most showed good knowledge, adopted positive attitudes and most implemented good practices towards rabies. No households in Madingane and only one (3.4%) in Bingo had good knowledge and good practices of rabies, respectively. More male respondents had good knowledge 28 (19%), positive attitudes 130 (88.4%) and good practices 20 (13.6%) than females. The 36 to 42 years age group was the one with the most respondents 6 (26.1%) demonstrating good knowledge of rabies, while for attitudes 28 (93.3%) and good practices 5 (19.2%), the highest results were observed in individuals with ages between 22-28 and 43-49 years, respectively. With regard to education, two (28.6%) and seven (100%) of respondents with secondary education, had good knowledge and demonstrated positive attitudes towards rabies, respectively. Few respondents, that is, 9 (10.6%) of those with no education and none with secondary education, adopted respectively good practices in relation to rabies. ~~With a distribution of 25%,~~ one public servant ~~was the one who~~ had the best level of knowledge of rabies. Those who were in a lower proportion with regard to positive attitudes and good practices were, ~~among others~~, unemployed 16 (76.2%) and students (none), respectively.

**Table 1. Test of (χ^2^) associations of households’ level of knowledge, attitudes and practices towards rabies and some independent variables in LNP, Mozambique**

| **Factors** | **Knowledge** | | | | | **Attitudes** | | | | | **Practices** | | | | |
| --- | --- | --- | --- | --- | --- | --- | --- | --- | --- | --- | --- | --- | --- | --- | --- |
|  | Good (%) | Poor (%) | χ^2^ | Df | P value | Positive (%) | Negative (%) | χ^2^ | Df | P value | Good (%) | Poor (%) | χ^2^ | Df | P value |
| Villages |  |  |  |  |  |  |  |  |  |  |  |  |  |  |  |
| Macaringue | 12 (29.3) | 29 (70.7) | 15.331 | 7 | 0.032 | 35 (85.4) | 6 (14.6) | 2.721 | 7 | 0.910 | 5 (12.2) | 36 (87.8) | 11.134 | 7 | 0.133 |
| Machamba | 6 (26.1) | 17 (73.9) |  |  |  | 20 (87.0) | 3 (13.0) |  |  |  | 1 (4.3) | 22 (95.7) |  |  |  |
| Munhamane | 5 (17.2) | 24 (82.8) |  |  |  | 25 (86.2) | 4 (13.8) |  |  |  | 5 (17.2) | 24 (82.8) |  |  |  |
| Mavoze | 12 (27.9) | 31 (72.1) |  |  |  | 39 (90.7) | 4 (9.3) |  |  |  | 11 (25.6) | 32 (74.4) |  |  |  |
| Madingane | 0 (0) | 26 (100) |  |  |  | 21 (80.8) | 5 (19.2) |  |  |  | 3 (11.5) | 23 (88.5) |  |  |  |
| Mahlaúle | 1 (5.9) | 16 (94.1) |  |  |  | 15 (88.2 | 2 (11.8) |  |  |  | 1 (5.9) | 16 (94.1) |  |  |  |
| Cunze | 5 (20.0) | 20 (80.0) |  |  |  | 21 (84.0) | 4 (16.0) |  |  |  | 4 (16.0) | 21 (84.0) |  |  |  |
| Bingo | 3 (10.3) | 26 (89.7) |  |  |  | 27 (93.1) | 2 (6.9) |  |  |  | 1 (3.4) | 28 (96.6) |  |  |  |
| Gender |  |  |  |  |  |  |  |  |  |  |  |  |  |  |  |
| Male | 28 (19.0) | 119 (81.0) | 0.007 | 1 | 0.934 | 130 (88.4) | 17 (11.6) | 0.610 | 1 | 0.435 | 20 (13.6) | 127 (86.4) | 0.031 | 1 | 0.860 |
| Female | 16 (18.6) | 70 (81.4) |  |  |  | 73 (84.9) | 13 (15.1) |  |  |  | 11 (12.8) | 75 (87.2) |  |  |  |
| Age |  |  |  |  |  |  |  |  |  |  |  |  |  |  |  |
| 18-21 | 4 (13.8) | 25 (86.2) | 2.733 | 6 | 0.842 | 25 (86.2) | 4 (13.8) | 1.786 | 6 | 0.938 | 1 (3.4) | 28 (96.6) | 8.593 | 6 | 0.198 |
| 22-28 | 5 (16.7) | 25 (83.3) |  |  |  | 28 (93.3) | 2 (6.7) |  |  |  | 2 (6.7) | 28 (93.3) |  |  |  |
| 29-35 | 5 (18.5) | 22 (81.5) |  |  |  | 23 (85.2) | 4 (14.8) |  |  |  | 2 (7.4) | 25 (92.6) |  |  |  |
| 36-42 | 6 (26.1) | 17 (73.9) |  |  |  | 20 (87.0) | 3 (13.0) |  |  |  | 3 (13.0) | 20 (87.0) |  |  |  |
| 43-49 | 3 (11.5) | 23 (88.5) |  |  |  | 23 (88.5) | 3 (11.5) |  |  |  | 5 (19.2) | 21 (80.8) |  |  |  |
| 50-60 | 20 (21.3) | 74 (78.7) |  |  |  | 81 (86.2) | 13 (13.8) |  |  |  | 18 (19.1) | 76 (80.9) |  |  |  |
| > 60 | 1 (25.0) | 3 (75.0) |  |  |  | 3 (75.0) | 1 (25.0) |  |  |  | 0 (0) | 4 (100) |  |  |  |
| Education |  |  |  |  |  |  |  |  |  |  |  |  |  |  |  |
| Primary | 35 (24.8) | 106 (75.2) | 9.968 | 2 | 0.007 | 123 (87.2) | 18 (12.8) | 1.153 | 2 | 0.562 | 22 (15.6) | 119 (84.4) | 2.264 | 2 | 0.322 |
| Secondary | 2 (28.6) | 5 (71.4) |  |  |  | 7 (100) | 0 (0) |  |  |  | 0 (0) | 7 (100) |  |  |  |
| None | 7 (8.2) | 78 (91.8) |  |  |  | 73 (85.9) | 12 (14.1) |  |  |  | 9 (10.6) | 76 (89.4) |  |  |  |
| Occupation |  |  |  |  |  |  |  |  |  |  |  |  |  |  |  |
| Farmer | 36 (18.7) | 157 (81.3) | 1.372 | 4 | 0.849 | 170 (88.1) | 23 (11.9) | 3.854 | 4 | 0.426 | 27 (14.0) | 166 (86.0) | 1.596 | 4 | 0.809 |
| Unemployed | 5 (23.8) | 16 (76.2) |  |  |  | 16 (76.2) | 5 (23.8) |  |  |  | 2 (9.5) | 19 (90.5) |  |  |  |
| Public servant | 1 (25.0) | 3 (75.0) |  |  |  | 4 (100) | 0 (0) |  |  |  | 1 (25.0) | 3 (75.0) |  |  |  |
| Student | 0 (0) | 4 (100) |  |  |  | 4 (100) | 0 (0) |  |  |  | 0 (0) | 4 (100) |  |  |  |
| Other | 2 (18.2) | 9 (81.8) |  |  |  | 9 (81.8) | 2 (18.2) |  |  |  | 1 (9.1) | 10 (90.9) |  |  |  |

**Health Practitioners (HPs)**

Forty two medical staff, from eight health centres completed the questionnaire. Nurses comprised the greatest proportion (n=19; 45%) (Fig 6). Twenty-four (57.1%) of respondents were female. The average length of service was 4.57 years and the median number of years of service was two (range: 1 to 30 years).

|  |  |
| --- | --- |
| Nurse | 19 |
| Physician | 2 |
| Care Assistant | 11 |
| Physician Assistant | 8 |
| Technician | 2 |

**Fig 6. Demographic characteristics of Massingir’s health practitioners**

**Health practitioners’ knowledge of rabies ~~disease~~**

Thirty two (76.2%) of the personnel correctly identified rabies as being caused by a virus, while 36 (85.7%) stated that the dog was the most important source of rabies in Mozambique. Of those who didn’t identify the dog correctly, five were in their first year of service and one was in their second year. When asked about transmission routes, 37 (88.1%) correctly identified dog bites as an important transmission route for rabies. However, only 11 (26.2%) correctly identified saliva and one (2.4%) identified raw meat as a threat. Almost all (n = 40; 95.2%) respondents believed they knew symptoms associated with rabies and most (n = 35; 87.5%) knew the disease was fatal. However, only 16 (40.0%) correctly identified the early, flu-like symptoms of rabies in humans. Similarly, half of interviewees correctly indicated hydrophobia as a symptom but only six (14.3%) identified paralysis. In all these cases, there was no significant difference between those with more advanced training (e.g. nurses) and the assistants (~~Fishers exact test,~~ p>0.05).

**HPs knowledge of wound categorization**

Twenty nine of the 42 interviewees ~~(69.0%)~~ confirmed they knew about the WHO classification of rabies exposure and, of these, most (n=19; 65.5%) were correct in identifying mild (Category I) exposure. However, only three personnel (10.3%) correctly identified all exposure categories (Table 2). Concerning the knowledge of the relationship between the site/ severity of the bite wound and the period of RABV incubation, over half of HPs (57.1%) were not aware about this relationship.

**Table 2. HPs’ knowledge of wound categorization in Massingir District (n=42)**

| **Variables** | **Frequency (%)** | | | | **Guidelines** |
| --- | --- | --- | --- | --- | --- |
|  | **CAT I** | **CAT II** | **CAT III** | **Don’t know** |  |
| Rabies exposure categories |  |  |  |  |  |
| Touching or feeding animals, licks on intact skin | 19 (65.5) | 0 (0) | 0 (0) | 23 (35.5) | CAT I |
| Nibbling of uncovered skin, minor scratches/abrasions without bleeding | 0 (0) | 9 (27.6) | 0 (0) | 33 (73.4) | CAT II |
| Single transdermal bites or scratches, licks on broken skin | 0 (0) | 0 (0) | 11 (34.5) | 31 (66.5) | CAT III |
| Multiple transdermal bites or scratches, licks on broken skin | 0 (0) | 0 (0) | 17 (58.6) | 25 (42.4) |  |
| Contamination of mucous membranes or broken skin with saliva | 0 (0) | 0 (0) | 8 (27.6) | 34 (73.4) |  |

**HPs attitudes and practices towards wound bite**

Thirty-eight respondents confirmed they knew about wound management; Thirty-seven (97.4%) advised bite wounds should be treated with soapy water, but significantly fewer (n = 7; 18.4%) felt that antiseptic was required. Interviewees with more than two years’ experience were more likely to know this than their less experienced colleagues (33.3% versus 12.5%), but this was not significantly different (95% CI = 6.6% - 44.2%; p = 0.1359) (Fig 7). Seven interviewees (16.7%) reported that they knew the importance of the bite location and severity and its association with the incubation period and onset of symptoms.

Attitudes and practices towards bite wound management (%)

|  |  |
| --- | --- |
| Washing wound with soapy water | 97.3684 |
| Washing wound with soapy water+antiseptic | 18.4211 |
| Applying only antiseptic | 2.63158 |
| Wound suturing | 2.63158 |
| None | 10.5263 |

**Fig 7. Frequency of health practitioners’ responses regarding bite wound care management practices (n=42)**

Thirty four HPs (89.4%) indicated they were aware of advice to be given about a suspect rabid dog. However, only thirteen (30.9%) correctly identified the necessity for isolating suspect dogs up to ten days. A significantly higher proportion of interviewees with more than two years’ experience were more likely to know this than their less experienced colleagues (56.3% versus 22.2%), (95% CI = 1.5% - 58.6%; p = 0.044).

**Knowledge of rabies prevention methods**

In respect of rabies prevention, most respondents 35 (83.3%) correctly reported that parenteral dog vaccination was a valuable preventive measure, however, substantially fewer identified other useful measures such as destruction of suspect animals (n = 14; 33.3%), public education (n = 11; 26.2%) or pre-exposure prophylaxis (n = 7; 16.7%).

**Knowledge of WHO guidelines for the recommended route(s) and site(s) for the anti-rabies vaccination**

Based on the World Health Organization (WHO) recommendations for the route and sites for rabies vaccine administration, 59.5% of HPs stated properly that rabies vaccine could be administered via intramuscular (IM), while 21.4% correctly reported intradermal (ID) as another route of application. On the other hand, 24 (57.1%) of HPs, ~~that is, the majority,~~ correctly answered that the site of application of the anti-rabies vaccine was in the deltoid muscle and nine (21.4%) also mentioned correctly the thigh as the other region for the vaccine application.

**Knowledge of post-exposure prophylaxis (PEP) management according to the category of exposure**

When asked about the best strategy to handle Category I, II and III patients, relatively few respondents identified the correct measures~~:~~. for Category I – three (7.1%), for Category II - seven (16.7%) and for Category III – 12 (28.6%) (Table 3).

Table 3. HPs’ knowledge of post-exposure prophylaxis (PEP) management according to the category of exposure in Massingir District, Mozambique (n=42)

| **Variables*** | **CAT I** | **CAT II** | **CAT III** | **Guidelines** |
| --- | --- | --- | --- | --- |
|  | **~~Frequency (%)~~** | | |  |
| ~~DK~~ | ~~39 (92.9)~~ | ~~35 (83.3)~~ | ~~30 (71.4)~~ | CAT I (N)  CAT II (WM+V)  CAT III (WM+V+RIG) |
| N | 3 (7.1) | - | - |  |
| WM+V | - | 7 (16.7) | - |  |
| WM+V+RIG | - | - | 12 (28.6) |  |

*Variables (DK-Don’t know; N-Nothing; WM-Wound management; V-Vaccine; RIG-Rabies immunoglobulin)

**Global results regarding the level of knowledge, attitudes/practices within health practitioners**

~~For global results of the level of knowledge and attitudes/practices towards rabies,~~ in general, of 42 interviewees, seven respondents (16.7%) had good knowledge, whilst 14 (33.3%) adopted adequate attitudes/practices towards the disease.

**Associations between variables under study within health practitioners**

The results in Table 4 show that there was a statistically significant difference ~~(p <0.05)~~ between knowledge of rabies, gender and occupation. All respondents (100%) of Machamba and Cubo Health posts showed good knowledge and adopted adequate attitudes/practices of rabies. More male respondents had good knowledge (33.3%) and adequate attitudes/practices (50%) than females. The age group between 36 and 40 years was the one with the most respondents (50%) demonstrating good knowledge of rabies, while for attitudes/practices, highest results were observed in individuals aged between 31-35 years (66.7%), respectively. With regard to years of service, those with 11-15 years (100%) and four (100%) HPs with 11 years onwards of practice had good knowledge and adequate attitudes/practices towards rabies, respectively. All physicians (100%) and five physician assistant (62.5%) were the ones who had the best levels of knowledge and adequate attitudes/practices of rabies~~, respectively~~.

Table 4. ~~Test of (χ~~^~~2~~^~~) associations of~~ HPs’ level of knowledge, attitudes and practices towards rabies and some independent variables in Massingir district, Mozambique ~~(n = 42)~~

| **Factors** | **Knowledge** | | | | | **Attitudes/Practices** | | | | |
| --- | --- | --- | --- | --- | --- | --- | --- | --- | --- | --- |
|  | Good (%) | Poor (%) | χ^2^ | Df | P value | Adequate (%) | Inadequate (%) | χ^2^ | Df | P value |
| Villages |  |  | 9.382 | 7 | 0.226 |  |  |  |  |  |
| Macaringue | 0 (0) | 4 (100) |  |  |  | 2 (50.0) | 2 (50.0) | 8.591 | 7 | 0.283 |
| Machamba | 1 (100) | 0 (0) |  |  |  | 0 (0) | 1 (100) |  |  |  |
| Massingir-Sede | 5 (22.7) | 17 (77.3) |  |  |  | 8 (36.4) | 14 (63.6) |  |  |  |
| Mavoze | 0 (0) | 2 (100) |  |  |  | 0 (0) | 2 (100) |  |  |  |
| Cubo | 0 (0) | 1 (100) |  |  |  | 1 (100) | 0 (0) |  |  |  |
| Chibotane | 0 (0) | 3 (100) |  |  |  | 2 (66.7) | 1 (33.3) |  |  |  |
| Mucatine | 0 (0) | 6 (100) |  |  |  | 0 (0) | 6 (100) |  |  |  |
| Zulo | 1 (33.3) | 2 (66.7) |  |  |  | 1 (33.3) | 2 (66.7) |  |  |  |
| Gender |  |  |  |  |  |  |  |  |  |  |
| Male | 6 (33.3) | 12 (66.7) | 6.300 | 1 | 0.031~~*~~ | 9 (50.0) | 9 (50.0) | 3.938 | 1 | 0.096* |
| Female | 1 (4.2) | 23 (95.8) |  |  |  | 5 (20.8) | 19 (79.2) |  |  |  |
| Age |  |  |  |  |  |  |  |  |  |  |
| 18-24 | 1 (9.1) | 10 (90.9) | 3.055 | 4 | 0.549 | 2 (18.2) | 9 (81.8) | 4.074 | 4 | 0.396 |
| 25-30 | 4 (16.4) | 20 (83.3) |  |  |  | 9 (37.5) | 15 (62.5) |  |  |  |
| 31-35 | 1 (33.3) | 2 (66.7) |  |  |  | 2 (66.7) | 1 (33.3) |  |  |  |
| 36-40 | 1 (50.0) | 1 (50.0) |  |  |  | 1 (50.0) | 1 (50.0) |  |  |  |
| >40 | 0 (0) | 2 (100) |  |  |  | 0 (0) | 2 (100) |  |  |  |
| Years of service |  |  |  |  |  |  |  |  |  |  |
| 1-5 | 4 (12.9) | 27(87.1) | 6.630 | 3 | 0.085 | 9 (29.0) | 22 (71.0) | 6.829 | 3 | 0.078 |
| 6-10 | 2 (28.6) | 5 (71.4) |  |  |  | 5 (71.4) | 2 (28.6) |  |  |  |
| 11-15 | 1 (100) | 0 (0) |  |  |  | 0 (0) | 1 (100) |  |  |  |
| > 15 | 0 (0) | 3 (100) |  |  |  | 0 (0) | 3 (100) |  |  |  |
| Occupation |  |  |  |  |  |  |  |  |  |  |
| Nurse | 2 (10.5) | 17 (89.5) | 11.770 | 4 | 0.019 | 6 (31.6) | 13 (68.4) | 5.457 | 4 | 0.241 |
| Care assistant | 1 (9.1) | 10 (90.9) |  |  |  | 2 (18.2) | 9 (81.8) |  |  |  |
| Physician assistant | 2 (25.0) | 6 (75.0) |  |  |  | 5 (62.5) | 3 (37.5) |  |  |  |
| Physician | 2 (100) | 0 (0) |  |  |  | 1 (50.0) | 1 (50.0) |  |  |  |
| Technician | 0 (0) | 2 (100) |  |  |  | 0 (0) | 2 (100) |  |  |  |

* Fisher Exact test

**Discussion and conclusion**

Establishing effective rabies prevention and control programs involves the assessment of levels of knowledge, attitudes and practices of the disease within local communities [53,54]. As long as the population of the LNP does not know or has doubts that rabies is a preventable disease, it can mean that not only their dogs, but also their livestock, which are their sources of wealth, are at serious risk of exposure to the disease. In fact, in 2018, the average vaccination coverage achieved in the country was very low, ~~that is,~~ 8.2% of about 3 011 656 animals at risk [55].

The predominance of males dogs observed in this study may be due to the fact that owners wanted to avoid the birth of undesirable puppies and the nuisance of a bitch in oestrus that attracts intact males and results in fighting. A low number of respondents with sterilised dogs demonstrated not only a lack of awareness of the importance of this procedure on animal rabies control [56], but also a lack of financial and logistical resources among LNP communities. According to Kitala et al. [57] surgical sterilization of dogs is a specialized procedure and conceivably out of reach of most of rural populations.

Most of LNP dogs spend their time roaming freely, foraging for food, but they always return to their household of origin. Moreover, the low socio-economic status, cultural and rural settlement (houses with no fences) characteristics of the LNP communities also hinders the practice of dog confinement. These practices were not dissimilar to elsewhere such as in Kenya (69%) [57,58], 79% in Madagascar [59] and 55% in Mali [60]. Confinement, rather than a measure of responsible dog ownership, can also minimize the risk of contracting or spreading of rabies, in addition to helping to plan more effective parenteral vaccination campaigns.

The general willingness shown by most respondents to seek a health centre in cases of bites is a good starting point for mitigating the risk of rabies. However, these actions must be reinforced and improved, mainly in relation to wound washing with soapy water, including the minimum washing time. In fact, in this survey, as also reported in other studies in Ethiopia [61,62], few respondents (24.5%) correctly indicated that they would wash their wounds with soapy water, a simple first aid measure that could substantially reduce (for a five-fold decrease) the risk of contracting rabies [63].

Poor knowledge and practices with regards to rabies within the LNP communities are matters of a great concern, especially concerning the causative agent, clinical signs and methods of prevention. Awareness and educational campaigns should be carried out more routinely in the region. World Rabies Day could be one of the most important events to achieve these objectives, but in LNP and similarly to what was reports in communities in Ethiopia by Alie et al. [64] and Digafe et al. [61], with about 100%, respectively, the elders of the family, seem to play a key role in disseminating knowledge about the disease. Nevertheless, it should be noted that most study respondents correctly mentioned the mode of transmission of the disease to humans. The higher level of perception observed may due to the fact that most respondents attended primary school at least. Despite good knowledge of the mode of transmission and the main vector of rabies, the role of other species and wild animals was little known, which is extremely worrying given the location of these villages at the human-wildlife interface. In the study there was also a lack of general knowledge of rabies clinical signs in both animals and humans. Although death is the final outcome after the onset of rabies symptoms, greater awareness and community education on early detection could reduce the risk of exposure and enhance the communities’ abilities to make early and correct decisions in suspected cases. Although over three quarters of respondents were aware of vaccination of dogs as a preventive measure, this did not translate to action as fewer than 20% were able to demonstrate a vaccination certificate for the previous year. This was consistent with other published studies in Kenya [65], Sri Lanka [66] and Rwanda [67] where a range of 73.9% to 88.1% respondents were awareof rabies prevention through dog vaccination. Regardless of the reasons behind the low proportion, a minimum goal of 70% vaccination coverage and, together with PEP to reduce to zero cases of dog-mediated human rabies by 2030, joint actions between the local government and various partners are urgently required. This should include community education (for example, radio, television and community meetings) on the importance of vaccinating dogs and the provision of free or subsidised vaccines, especially in rural areas where low income is a major constraint. Destruction of an animal suspected of being rabid is not unique to Mozambique but has also been reported in Tanzania, Kenya and the DRC [65,68,69]. This negative practice contributes to under-reporting of rabies cases, since the cadaver will not be examined by an expert and samples will not be taken for laboratory confirmation. Cleaveland et al. [68] also pointed out that the attitude of elimination of suspected rabies dogs reduces the number of people who should receive the PEP.

Mavoze village showed the best results in terms of rabies knowledge, attitudes and practices. This is likely due to the fact that it is the most populous and the most developed of the eight villages in the study. Our results are in agreement with those of Awuni et al. [70] in Ghana, highlighting the influence of the location of households ~~(village) (χ~~^~~2~~^~~=112.59; P=0.001)~~ on variables related to knowledge of rabies. In addition to the influence of villages’ location, the probability of those with some level of education to have best knowledge of rabies was more significant than those who never attended school. Similar results in agreement with those found in our study were also reported from studies conducted in Nigeria (P=0.000) [71], Brazil (P<0.001) [50], Morocco (P<0.05) [72] and in Ethiopia (P=0.022) [64]. Epidemiological surveillance can be greatly improved if rural veterinary extension services together with community participation are actively involved in rabies control. For instance, over 95% of the study population were willing to register and vaccinate their dogs, thus providing opportunities for progress if government agencies were able to provide vaccination. In LNP, the points of great gathering of the population and their livestock, including dogs, are limited to points of vaccination, acaricidal baths as well as crop fields. These can be considered, alongside churches and schools, as strategic places for the implementation of community-based awareness and education campaigns of rabies.

**Health practitioners (HPs)**

One purpose of this investigation was to raise awareness and inform the political actors and managers of the health sectors about the need for training and strengthening the skills of the HPs in matters of rabies prevention and PrEP/PEP guidelines. The implementation of One-Health approaches, which may in turn improve the capacity for epidemiological surveillance and the control and prevention of cases of bites and rabies within the LNP communities, cannot be overemphasised. As a way of showing the existing gaps on rabies, the study revealed that nearly a quarter of HP respondents were not aware of the causative agent of rabies and the major animal source of the disease in Mozambique. Other studies have shown similar results for the “viral” cause (62%-77.5%) in India and in Pakistan [75,76] respectively, while awareness of the dog as the primary vector responsible for human rabies cases was higher in India [77] than in this study (98% versus 85.7%). For a health professional, mastery of such concepts mentioned previously is of paramount importance for making correct decisions about the best therapeutic or prophylactic approaches to the disease. Again, this underscores that training, retraining courses and capacity building actions are essential. The fact that not all HPs in Massingir knew of the primary route of rabies transmission to humans linked with some misconception (e.g. blood contact) on the subject also illustrated to need for health authorities to promote educational courses that can improve the knowledge levels of their professionals. Such programs, in turn, would help to save resources for PEP treatment and minimise the concerns that would result from non-exposure to rabies. More than recognizing clinical signs of rabies in humans, it is important to take precautions in patients with history of wound bites, scratches, or other contact with potentially infectious vector. Apart from death, a few respondents mentioned other clinical signs indicative of encephalitis due to rabies. Our findings are in complete disagreement with those found out in a study carried out by Kishore et al. [52], where hydrophobia was one of the clinical signs most recognized by the health workers of Uttarakhand, in India (95.7%). These gaps can be justified by the confusion attributed to the disease compared to others of an encephalitic nature such as malaria and also due to the rare admission of suspected cases of rabies to local health centers. A study conducted by Mallewa et al. [78] in Malawi, pointed out that a central nervous system infection should not only be attributed to cerebral malaria, although most of the clinical signs are similar to those of rabies. Wound management and rabies PEP depend on an appropriate pre-assessment and correct wound classification [79], but the lack of general awareness among HPs in this regard may delay or worsen timeous and appropriate measures. The low proportion of HPs who would correctly advise the monitoring of biting animals for ten days may be due to the fact that most LNP households do not have a fenced back yard, let alone kennels. In addition, the apparent lack of communication and coordination between HPs and animal health officers seems to complicate rabies prevention actions, as it is up to the veterinary sector's staff to confine and closely monitor animals suspected of rabies or those responsible for human bites. The measures or advice given unilaterally by the HPs to patients or victims without addressing the one-health approach were also mentioned and observed by Malhotra et al. [80], Malhotra et al. [81] in India, and by Niang et al. [82] in Senegal. The association between location of the bite and the wound severity (scratch versus deep skin penetration) is likely to affect the outcome into clinical rabies [30]. In the present study, there was a lack of perception of the association between location of the bite and onset of clinical signs. This is very worrying scenario because it can result in death of many patients due to the lack of immediate administration of PEP according to the observed situation. Furthermore a considerable percentage of the study participants were unaware of guidelines concerning both site and route of rabies vaccinations, including the pre- and post-exposure prophylaxis scheme. The lack of refresher and updating courses on neglected diseases including rabies may be behind the concerned gaps. In Massingir District, less than half of HPs had specific background in the nursing area, and they do not always have access to practical training on anti-viral prophylaxis. The weak and slow level of development of the health sector, including the scarcity of rabies vaccine and RIGs, may also influence the lack of awareness of its use within the health staff. According to Tiwari et al. [83], the same scenario is also worrying in India, where it was reported that physicians are not adequately informed about the importance of administering vaccinations and human rabies immunoglobulins. In 2004, MISAU (Ministry of Health) launched a rabies handbook edited by Barreto et al.[84], in which all the basic information about the disease was described in a simple way. Surprisingly, the health practitioners still have gaps on knowledge of rabies and its prevention, as well as in the management of bites.

**Study limitations**

A major limitation of this study was the fact that there is still a tension between the government and the residents of LNP as regards the resettlement process started in 2006. In order to better understand the difficulties in accessing the villages and the recruitment of participants for this study, it is advised to read the research work carried out by Milgroom and Spierenburg [85]. The conclusions also take into account the low number of health professionals interviewed, an aspect derived from the shortage of human resources in the Massingir district health sector. This aspect has been a decisive factor that delays the efforts of the Government of Mozambique in the expansion and improvement of the provision of basic health services.

**Acknowledgements**

We are grateful to Dr Sérgio Salomão Bié (Limpopo National Park) for providing some data related to the Limpopo National Park, to Dr Guilhermina Sitoe (Former Health Director of the Massingir District) for allowing us to work at health post/centres of Massingir District. We are thankful to the communities of all the villages and to the health workers of all post and health centers in Massingir, especially to those who orally consented to participate in this study. We thank the Biotechlogy Centre for the logistics and to dr Remígio Mungói (District Services for Economic Activities of Massingir), Dr Vlademiro Magaia, Dr Ofélia Nhambirre, André Nhambir and Iara Gomes for their help in the field. We are grateful to Dr Abel Chilundo and Professor Alberto Pondja for assisting in the insertion, organisation and interpretation of the statistical data.

**Author Contributions**

M M: conceived and wrote the manuscript. D A: participated in the study design, statistical analysis, and reviewing of the manuscript. C T S: participated in the study design, and helped to write and edit the manuscript. J M F: participated in the project management and helped to write and edit the final manuscript.

**Conflicts of Interest**

The authors declare no conflict of interest. The funders had no influence in the design of the study, in the collection, analyses, or interpretation of data, in the writing of the manuscript, or in the decision to publish the results.

**Funding**

This research was funded by the “Communities on the move: animal and human health challenges” project, University of Pretoria and Institute of Tropical Medicine of Antwerp.

**References**

**1.**	Banyard AC, Horton DL, Freuling C, Müller T, Fooks AR. Control and prevention of canine rabies: The need for building laboratory-based surveillance capacity. Antiviral Research 2013 Jun;98(3):357-64.

**2.**	Chiou HY, Hsieh CH, Jeng CR, Chan FT, Wang HY, Pang VF. Molecular Characterization of Cryptically Circulating Rabies Virus from Ferret Badgers, Taiwan. Emerg Infect Dis 2014 May;20(5):790-8.

**3.**	Daszak P, Cunningham AA, Hyatt AD. Emerging infectious diseases of wildlife--threats to biodiversity and human health. science 2000;287(5452):443-9.

**4.**	Rupprecht CE, Hanlon CA, Hemachudha T. Rabies re-examined. The Lancet Infectious Diseases 2002;2(6):327-43.

**5.**	Mani RS, Madhusudana SN. Laboratory diagnosis of human rabies: recent advances. The Scientific World Journal 2013;2013.

**6.**	Horton DL, McElhinney LM, Marston DA, Wood JL, Russell CA, Lewis N, et al. Quantifying antigenic relationships among the lyssaviruses. J Virol 2010 Nov;84(22):11841-8.

**7.**	Wallace RM, Mehal J, Nakazawa Y, Recuenco S, Bakamutumaho B, Osinubi M, et al. The impact of poverty on dog ownership and access to canine rabies vaccination: results from a knowledge, attitudes and practices survey, Uganda 2013. Infectious diseases of poverty 2017;6(1):97.

**8.**	Weyer J, Szmyd-Potapczuk AV, Blumberg LH, Leman PA, Markotter W, Swanepoel R, et al. Epidemiology of human rabies in South Africa, 1983-2007. Virus Res 2011 Jan;155(1):283-90.

**9.**	Tordo N, Poch O, Ermine A, Keith G+. Primary structure of leader RNA and nucleoprotein genes of the rabies genome: segmented homology with VSV. Nucleic Acids Research 1986;14(6):2671-83.

**10.**	Hampson K, Coudeville L, Lembo T, Sambo M, Kieffer A, Attlan M, et al. Estimating the global burden of endemic canine rabies. PLoS Negl Trop Dis 2015;9.

**11.**	Jackman J, Rowan AN. Free-roaming dogs in developing countries: The benefits of capture, neuter, and return programs. In: Deborah Salem AR, editor. The state of the animals IV.Washington DC: Humane Society Press; 2007. p. 55-78.

**12.**	Singh R, Singh KP, Cherian S, Saminathan M, Kapoor S, Manjunatha Reddy GB, et al. Rabies - epidemiology, pathogenesis, public health concerns and advances in diagnosis and control: a comprehensive review. Vet Q 2017 Dec;37(1):212-51.

**13.**	Dodet B, Goswami A, Gunasekera A, de GF, Jamali S, Montalban C, et al. Rabies awareness in eight Asian countries. Vaccine 2008 Nov 25;26(50):6344-8.

**14.**	Felgate M. An Update from Mozambique. Rabid Bytes 2011;(21):6.

**15.**	Hasanov E, Zeynalova S, Geleishvili M, Maes E, Tongren E, Marshall E, et al. Assessing the impact of public education on a preventable zoonotic disease: rabies. Epidemiol Infect 2018 Jan;146(2):227-35.

**16.**	Nilsson M. Effect of rabies education programs on rabies awareness, attitudes towards dogs and animal welfare among children in Lilongwe, Malawi. Uppsala: Swedish University of Agricultural Sciences; 2014.

**17.**	Government of Mozambique. Strategy for the Control of Rabies (2010-2014). Council of Ministers 2010;42nd Session(23 November):1-19.

**18.**	Ministry of Agriculture and Food Security. National Agriculture Investment Plan 2014-2018. (Comprehensive Africa Agriculture Development Programme) 2014;1-100.

**19.**	Bilaide SF. Retrospective study of animal and human rabies occurences in Mozambique [Monograph] University Eduardo Mondlane. Maputo; 2019.

**20.**	Cuco ES, Liesegang G. Conflito homem fauna bravia: Caso do Parque nacional de Limpopo University Eduardo Mondlane; 2012.

**21.**	Bekker JL, Hoffman LC, Jooste PJ. Wildlife-associated zoonotic diseases in some southern African countries in relation to game meat safety: a review. The Onderstepoort journal of veterinary research 2012 Dec 5;79(1):E1-E12.

**22.**	Vial F, Cleaveland S, Rasmussen G, Haydon DT. Development of vaccination strategies for the management of rabies in African wild dogs. Biological Conservation 2006;131(2):180-92.

**23.**	WHO. Mozambique. Rabies country profile. World Health Organization 2013;https://[www.who.int/rabies/epidemiology/Rabies_CP_Mozambique_09_2013.pdf](http://www.who.int/rabies/epidemiology/Rabies_CP_Mozambique_09_2013.pdf).

**24.**	Osofsky SA, Cumming DHM, Kock MD. Transboundary management of natural resources and the importance of a 'One Health' approach. In: Fearn E, Woods W, editors. State of the Wild 2008-2009: A Global Portrait of Wildlife, Wildlands, and Oceans.Washington DC: Island Press; 2008. p. 89-98.

**25.**	Bragança LFJ. Caracterização do conhecimento da Raiva pelos estudantes da EP2 e Informantes chave na cidade e dois distritos da província de Nampula. Contributo para o Desenvolvimento de Estratégias de profilaxia da doença em Moçambique. Maputo: Universidade Eduardo Mondlane; 2005.

**26.**	Guimarães ELAM, Moreira SM. Variação da incidência da raiva humana no Brasil e em Moçambique de 2001 a 2007. Revista Científica Eletrônica de Medicina Veterinária 2012;18.

**27.**	Dzikwi AA, Ibrahim AS, Umoh JU. Knowledge, attitude and practice about rabies among children receiving formal and informal education in Samaru, Zaria, Nigeria. Global journal of health science 2012 Aug 9;4(5):132-9.

**28.**	Lembo T, Hampson K, Kaare MT, Ernest E, Knobel D, Kazwala RR, et al. The feasibility of canine rabies elimination in Africa: dispelling doubts with data. PLoS Negl Trop Dis 2010;4(2):e626.

**29.**	Tamirat K, Alemayehu L, Mulualem T. Community Perception towards Traditional Healers and Health Centers on Management of Dog Bites and its Relation with Veterinary Public Health Activities. J Vet Sci Ani Husb 2016;4(2):202.

**30.**	Tschopp R, Bekele S, Aseffa A. Dog Demography, Animal Bite Management and Rabies Knowledge-Attitude and Practices in the Awash Basin, Eastern Ethiopia. PLoS Negl Trop Dis 2016 Feb 22;10(2):e0004471.

**31.**	Ghosh S, Chowdhury S, Haider N, Bhowmik RK, Rana M, Prue Marma AS, et al. Awareness of rabies and response to dog bites in a Bangladesh community. Vet Med Sci 2016 Mar 11;2(3):161-9.

**32.**	Salomao C, Nacima A, Cuamba L, Gujral L, Amiel O, Baltazar C, et al. Epidemiology, clinical features and risk factors for human rabies and animal bites during an outbreak of rabies in Maputo and Matola cities, Mozambique, 2014: Implications for public health interventions for rabies control. PLoS Negl Trop Dis 2017 Jul;11(7):e0005787.

**33.**	WHO. Rabies. World Health Organization 2019.

**34.**	Lushasi K, Steenson R, Bernard J, Changalucha JJ, Govella NJ, Haydon DT, et al. One Health in practice: using Integrated Bite Case Management to increase detection of rabid animals in Tanzania. Frontiers in public health 2020;8:13.

**35.**	Sambo M, Cleaveland S, Ferguson H, Lembo T, Simon C, Urassa H, et al. The burden of rabies in Tanzania and its impact on local communities. PLoS Negl Trop Dis 2013;7(11):e2510.

**36.**	Hudson EG, Dhand N, Dürr S, Ward MP. A Survey of Dog Owners in Remote Northern Australian Indigenous Communities to Inform Rabies Incursion Planning. PLoS Negl Trop Dis 2016 Apr 26;10(4):e0004649.

**37.**	Sambo M, Lembo T, Cleaveland S, Ferguson HM, Sikana L, Simon C, et al. Knowledge, attitudes and practices (KAP) about rabies prevention and control: a community survey in Tanzania. PLoS Negl Trop Dis 2014;8(12):e3310.

**38.**	Schwitters A, Lederer P, Zilversmit L, Gudo PS, Ramiro I, Cumba L, et al. Barriers to health care in rural Mozambique: a rapid ethnographic assessment of planned mobile health clinics for ART. Glob Health Sci Pract 2015 Mar;3(1):109-16.

**39.**	INE. Anuário Estatístico 2016 - Moçambique (Statistical Yearbook 2016 – Mozambique). Instituto Nacional de Estatísticas (National Statistics Institute) 2017;9-108.

**40.**	INE. Anuário Estatístico 2018 - Província de Gaza. Instituto Nacional de Estatísticas (National Statistics Institute) 2018;1-56.

**41.**	Cambule AH, Smaling EMA. Assessment of soil organic carbon stocks in the Limpolo National Park: from legacy data to digital soil mapping University of Twente; 2013.

**42.**	Stalmans M, Gertenbach WPD, Carvalho-Serfontein F. Plant communities and landscapes of the Parque Nacional do Limpopo, Moçambique. Koedoe 2004;47(2):61-81.

**43.**	Massé F. Wildlife, cattle, and people in the Limpopo National Park: A more-than-human political ecology of conservation-induced displacement and resettlement. Toronto, Ontario: York University; 2013.

**44.**	Le Bel S, Murwira A, Mukamuri B, La Grange M, Czudek R, Taylor R. Human wildlife conflicts in southern Africa: riding the whirl wind in Mozambique and in Zimbabwe. In: J.López-Pujol, editor. The Importance of Biological Interactions in the Study of Biodiversity.Rijeka, Croatia: INTECH Open Access Publisher; 2011. p. 283-322.

**45.**	Witter R. Elephant-induced displacement and the power of choice: moral narratives about resettlement in Mozambique's Limpopo National Park. Conservation and Society 2013;11(4):406.

**46.**	Dohoo IR, Martin W, Stryhn HE. Veterinary epidemiologic research. Charlottetown: AVC Inc.; 2003.

**47.**	Simone E. Evaluation of the level of protection against Rabies in dogs and the awareness of dog owners regarding the disease in Manica Province, Mozambique [Preventive Veterinary Medicine]. Maputo. Mozambique: Faculty of Veterinary. University Eduardo Mondlane; 2016.

**48.**	Parque Nacional do Limpopo. Composição de agregados familiares de algumas zonas do Parque Nacional do Limpopo no âmbito do reassentamento (2014-2015). Ministério da Terra, Ambiente e Desenvolvimento Comunitário 2017.

**49.**	Thrusfield M. Veterinary Epidemiology. 3 ed. Oxford, UK: Blackwell Publishing company; 2007.

**50.**	da Costa LJC, Fernandes MEB. Rabies: knowledge and practices regarding rabies in rural communities of the Brazilian Amazon Basin. PLoS Negl Trop Dis 2016;10(2):e0004474.

**51.**	Kapoor P, Baig VN, Kacker S, Sharma M, Sharma M. A cross-sectional study of knowledge regarding rabies among attendees of anti-rabies clinic of a teaching hospital, Jaipur. Journal of family medicine and primary care 2019;8(1):194.

**52.**	Kishore S, Singh R, Ravi SK. Knowledge, attitude and practice assessment in health workers regarding rabies disease and its prevention in district Dehradun of Uttarakhand. Indian Journal of Community Health 2015;27(3):381-5.

**53.**	Kisiel LM, Jones-Bitton A, Sargeant JM, Coe JB, Flockhart DT, Palomar AR, et al. Owned dog ecology and demography in Villa de Tezontepec, Hidalgo, Mexico. Preventive veterinary medicine 2016;135:37-46.

**54.**	Morters MK, McKinley TJ, Restif O, Conlan AJ, Cleaveland S, Hampson K, et al. The demography of free-roaming dog populations and applications to disease and population control. J Appl Ecol 2014 Aug;51(4):1096-106.

**55.**	National Livestock Development Directorate. Mozambique relevant country data. Ministry of Agriculture and Rural Development; 2019. Report No.: In Timeframe (e.g. annual reports, total over the past 2 years).

**56.**	Reece JF, Chawla SK. Control of rabies in Jaipur, India, by the sterilisation and vaccination of neighbourhood dogs. Veterinary Record 2006;159(12):379-83.

**57.**	Kitala P, McDermott J, Kyule M, Gathuma J, Perry B, Wandeler A. Dog ecology and demography information to support the planning of rabies control in Machakos District, Kenya. Acta tropica 2001;78(3):217-30.

**58.**	Muriuki BJ. Knowledge, Attitude and Practices on Rabies in Kisumu and Siaya Counties, Kenya. MSc Thesis University of Nairobi 2016;52-5.

**59.**	Ratsitorahina M, Rasambainarivo JH, Raharimanana S, Rakotonandrasana H, Andriamiarisoa MP, Rakalomanana FA, et al. Dog ecology and demography in Antananarivo, 2007. BMC Veterinary Research 2009;5(1):21.

**60.**	Mauti S, Traoré A, Sery A, Bryssinckx W, Hattendorf J, Zinsstag J. First study on domestic dog ecology, demographic structure and dynamics in Bamako, Mali. Preventive veterinary medicine 2017;146:44-51.

**61.**	Digafe RT, Kifelew LG, Mechesso AF. Knowledge, attitudes and practices towards rabies: questionnaire survey in rural household heads of Gondar Zuria District, Ethiopia. BMC Res Notes 2015;8(1):400.

**62.**	Kabeta T, Deresa B, Tigre W, Ward MP, Mor SM. Knowledge, Attitudes and Practices of Animal Bite Victims Attending an Anti-rabies Health Center in Jimma Town, Ethiopia. PLoS Negl Trop Dis 2015 Jun 26;9(6):e0003867.

**63.**	Hampson K, Dobson A, Kaare M, Dushoff J, Magoto M, Sindoya E, et al. Rabies Exposures, Post-Exposure Prophylaxis and Deaths in a Region of Endemic Canine Rabies. PLoS Negl Trop Dis 2008 Nov 25;2(11):e339.

**64.**	Alie A, Assefa A, Derso S, Ayele B. Assessment of knowledge, attitude and practice on rabies in and around Debretabor, South Gondar, Northwest Ethiopia. Int J Basic Appl Virol 2015;4(1):28-34.

**65.**	Mucheru GM, Kikuvi GM, Amwayi SA. Knowledge and practices towards rabies and determinants of dog rabies vaccination in households: a cross sectional study in an area with high dog bite incidents in Kakamega County, Kenya, 2013. The Pan African medical journal 2014;19.

**66.**	Matibag GC, Kamigaki T, Kumarasiri PV, Wijewardana TG, Kalupahana AW, Dissanayake DR, et al. Knowledge, attitudes, and practices survey of rabies in a community in Sri Lanka. Environ Health Prev Med 2007 Mar;12(2):84-9.

**67.**	Ntampaka P, Nyaga PN, Niragire F, Gathumbi JK, Tukei M. Knowledge, attitudes and practices regarding rabies and its control among dog owners in Kigali city, Rwanda. PloS one 2019;14(8).

**68.**	Cleaveland S, Kaare M, Tiringa P, Mlengeya T, Barrat J. A dog rabies vaccination campaign in rural Africa: impact on the incidence of dog rabies and human dog-bite injuries. Vaccine 2003 May 16;21(17ΓÇô18):1965-73.

**69.**	Mbilo C+, Kabongo JB, Pyana PP, Nlonda L+, Nzita RW, Luntadila B, et al. Dog Ecology, Bite Incidence, and Disease Awareness: A Cross-Sectional Survey among a Rabies-Affected Community in the Democratic Republic of the Congo. Vaccines (Basel) 2019 Aug 26;7(3):98.

**70.**	Awuni B, Tarkang E, Manu E, Amu H, Ayanore MA, Aku FY, et al. Dog OwnersΓÇÖ Knowledge about Rabies and Other Factors That Influence Canine Anti-Rabies Vaccination in the Upper East Region of Ghana. Tropical medicine and infectious disease 2019;4(3):115.

**71.**	Ameh VO, Dzikwi AA, Umoh JU. Assessment of knowledge, attitude and practice of dog owners to canine rabies in Wukari Metropolis, Taraba State Nigeria. Global journal of health science 2014;6(5):226.

**72.**	Bouaddi K, Bitar A, Bouslikhane M, Ferssiwi A, Fitani A, Mshelbwala PP. Knowledge, Attitudes, and Practices Regarding Rabies in El Jadida Region, Morocco. Veterinary sciences 2020;7(1):29.

**73.**	Mbaipago N, Mindekem R, Oussiguere A, Moyengar R, Naïssengar K, Madjadinan A, et al. Rabies knowledge and practices among human and veterinary health workers in Chad. Acta tropica 2020;202:105180.

**74.**	Bourhy H, Troupin C+, Faye O, Meslin FX, Abela-Ridder B, Kraehenbuhl JP. Customized online and onsite training for rabies-control officers. Bulletin of the world Health Organization 2015;93:503-6.

**75.**	Bhalla S, Mehta JP, Singh A. Knowledge and practice among general practitioners of Jamnagar city regarding animal bite. Indian Journal of Community Medicine 2005;30(3):94.

**76.**	Shah SF, Jawed M, Nooruddin S, Afzal S, Sajid F, Majeed S, et al. Knowledge and practices among the general practitioners of Karachi regarding dog bite management. JPMA The Journal of the Pakistan Medical Association 2009;59(12):861-4.

**77.**	Amrita S, Sudip B, Chintan B, Aniruddha G, Naresh M, Dipesh P. An assessment of knowledge of prevention and management of Rabies in interns and final year students of Shri MP Shah Government Medical College, Jamnagar, Gujarat. Journal of Research in Medical and Dental Science 2013;1(2):62.

**78.**	Mallewa M, Fooks AR, Banda D, Chikungwa P, Mankhambo L, Molyneux E, et al. Rabies encephalitis in malaria-endemic area, Malawi, Africa. Emerg Infect Dis 2007 Jan;13(1):136-9.

**79.**	WHO. Rabies vaccines: WHO position paper, April 2018 Recommendations. Vaccine 2018;36(37):5500-3.

**80.**	Malhotra V, Balgir RS, Watts A, Kaur S, Nirwan PS, Cheema R. Awareness regarding animal bite management among resident doctors of tertiary care institute of Northern India. Medical Journal of Dr DY Patil University 2017;10(4):359.

**81.**	Malhotra V, Singh A, Balgir RS. Knowledge and practices about animal bite management among government doctors posted at primary health-care settings of district Patiala in Punjab. Journal of Current Research in Scientific Medicine 2018;4(1):47.

**82.**	Niang K, Tine JA, Ndao AB, Diongue FB, Diallo AI, Faye A, et al. Knowledge and Attitudes of Health Care Providers and the Population about Rabies in Sokone Health District, Senegal. Open Journal of Preventive Medicine 2020;10(04):63.

**83.**	Tiwari HK, Vanak AT, O'Dea M, Robertson ID. Knowledge, attitudes and practices towards dog-bite related rabies in para-medical staff at rural primary health centres in Baramati, western India. PloS one 2018;13(11):e0207025.

**84.**	Barreto A, Matos CS, Gujral L. MANUAL DA RAIVA - PROFILAXIA E VIGILÂNCIA EPIDEMIOLÓGICA. 1^st^ ed. Maputo: Imagem Global; 2004.

**85.** Milgroom J, Spierenburg M. Induced volition: Resettlement from the Limpopo National Park, Mozambique. Journal of Contemporary African Studies 2008;26(4):435-48.
