# Supplementary material for: Knowledge, attitudes, practices (KAP) and control of rabies among community households and health practitioners at the human-wildlife interface in Limpopo National Park, Massingir District, Mozambique
Source: PLoS Negl Trop Dis. 2022 Mar 7;16(3):e0010202. doi: 10.1371/journal.pntd.0010202 (PMC8929695; doi:10.1371/journal.pntd.0010202)
Supplement: Attachment — Submitted filename: PNTD-D-20-01733 LETTER REVIEWERS COMMENTS TRACK CHANGES.docx [file pntd.0010202.s005.docx]

**29^th^ January 2021**

Daniel Leo Horton,

Associate Editor

PLOS Neglected Tropical Diseases

Dear Prof. Horton

Subject: **PNTD-D-20-01733 - [EMID:5c2c80d822c7013d]**

The authors would like to thank you for your correspondence regarding the review of our manuscript “Knowledge, attitudes, practices (KAPs) and control of rabies among community households and health practitioners at the human-wildlife interface in Limpopo National Park, Massingir District, Mozambique” by Milton Mapatse, Claude Sabeta, José Fafetine, Darrell Abernethy. Responses to the reviewers' comments and changes made to the manuscript are presented below. The reviewers’ comments are presented in italics, the author’s response in bold text and changes to the text are indicated in red. Pages numbers are based on the revised (with track changes) version of the manuscript.

**Major revisions**

Reviewer #1:

*The objective should be refined, its too long delete unnecessary words.*

**Response: The objective has been shortened both in the abstract (Page 2) and along the text on page 6.**

*I haven't seen the testable hypothesis in the manuscript. The study design is appropriate, well planned by the investigator. these two types of population are clearly discussed in the document. The sample size is fine but unfortunately no hypothesis is tested in this study.*

**Response: The authors agree that no hypothesis was tested, which reflected the lack of information in the communities of the Limpopo National Park, and the primary need to gather descriptive data. Thus the major goal of this study was to help the health and veterinary authorities design more effective education, awareness and prevention programmes for rabies according to this baseline information. This will enable future studies to develop and test relevant hypotheses.**

*looking at the abstract, methodology section . what criteria did you use choose the households please include it in the abstract.*

**Response: The study objectives were first explained to community leaders of the villages who then identified households for participation in the study. Representatives aged ≥18 years from each household were then selected purposively after being asked for their oral consent. The authors included these criteria in the abstract as suggested (Page 2).**

*still under the abstract the data analysis tool is not mentioned .*

**Response: This has now been included in the abstract (Page 2).**

*i suggest we should have a subtitle for ethical or regulatory requirements.*

**Response: We have added a subtitle on the subject of ethical requirements (Page 11).**

*According line 207,What is that model proposed elsewhere? we need it to know that name of that model.*

**Response: In fact, it was a misuse of the term "model". The authors intended to say “scoring system” used elsewhere in similar studies. We have therefore improved (proofreading) the sentence by removing the word “model” and replacing it in the manuscript with the correct expression (scoring system) (Page 11).**

**Results**

*Reviewer #1: line 34, statements such as in general are vague. Does such a finding reflect on the entire population.*

**Response:** **Reviewer #1 is absolutely right. We thank him for suggesting that we use scientifically correct terms to express our ideas and/or results. We have therefore replaced the word “in general”, with “Almost” (Page 2).**

*More work needs to be done, some results can be put in a tabular form. and concentrate on the key findings that you think can bring out the practical aspect, that can advocate for policy formulation and control of rabies in LNP.*

**Response: The Reviewer #1 is absolutely right. We have made the appropriate changes and some of the results have been tabulated. Some of the tabulated results will be published as supplementary information. This will make it easier for the Veterinary and Health authorities to understand and make decisions, as the Reviewer #1 rightly said. Among the tabulated results are those relating to dog bite experience, treatment applied and health-seeking behaviours to be adopted within households (Table 2) (Page 15); Table 3 (Frequency of dog vaccination by village and reasons for non-vaccination against rabies; Page 16); For the supplementary tables we will include Table S1 (Knowledge of rabies, its transmission, clinical signs, and disease outcome are presented in the supporting information; stated in Page 17); Table S2 (Knowledge and attitudes towards rabies prevention, Page 20) and Table S3 (Knowledge of rabies causative agent, main animal source of rabies in Mozambique, mode of transmissions, clinical features, incubation period including concept of rabies categories of exposure (Page 26).**

*Line 313 & 314 Global results regarding the level of knowledge, attitudes and practices towards rabies within households. is this true? Are you conducting this research on a global scale or in LNP ?*

**Responses: We have deleted these terms (Page 21).**

*Please throw more light on this subtopic.*

**Response: We appreciate this comment and to spice up the subtopic, the authors also included the main variables used to assess the level of knowledge, attitudes and practices towards rabies control and prevention (Page 21).**

**Conclusions**

*Reviewer #1: The results and conclusions are disconnected. Please ensure that your document flows logically.*

**Response: The authors included a heading “Conclusions and recommendations” regarding households and health practitioners (Page 39).**

*Discussion and conclusion line 437 & 438 the emphasis is more about livestock. Please consider the public health relevance in your discussion, since your study is about rabies at human-wildlife interphase.*

**Response: We have added the word “community” to elucidate the risk that the local population poses with regard to rabies (Page 33).**

**Summary and General Comments**

*Reviewer #1: This is good work and can be publishable , the abstract should be written well to reflect the major components of the study. clearly outline the research ethics let it stand out. reduce on the wording in the entire document. Major revisions should be done.*

**Response: We are grateful for the comments. The authors carried out the corrections and the major revision as suggest throughout the manuscript.**

**Major revisions**

*Reviewer #2*

*This is a significant study particularly in Africa where rabies is prevalent. However, I have some issues on the sample size and method on respondent selection. The authors need to describe them fully in the paper.*

**Response: Thank you for the advice. We have provided more details in the paper in respect of sample size (formulas, calculations) and selection of respondents (Pages 9-10).**

*In addition, the paper needs to be fully proofread to be acceptable for publication.*

**Response: The authors thank the Reviewer #2 for the observation and recommendation. Two of the co-authors are native English speakers, and have made the manuscript proofreading and English review to meet all acceptable language requirements for publication.**

**Minor revisions for the comments found in the manuscript**

Comment [A1]: *What is LNP?*

**Response: We have included the meaning in the text (Page 3). “Limpopo National Park”**

Comment [A2]: *here*

**Response: we replaced the word “*here.”* by “…in this study…” (Page 3).**

Comment [A3]: *(n=44)*

**Response: Page 4, “(n=44)” was inserted.**

Comment [A4]: *ratio of*

**Page 5. The following words were incorporated in the text “ratio of”.**

Comment [A5]: *is*

**Page 6. After proofreading “is” was not added to the text (should be on page 6).**

**.**

Comment [A6]: *This*

**After proofreading “This” was not added to the text (should be on page 6).**

Comment [A7]: *Explain why weight the sample. Most people know about rabies. That is the reason why the sample size is so small. Using online sample calculators, which uses the same formula like you did, a sample of 322 would be necessary at the same CL and ME. With 233, the ME is 6%*

**Pages 9 and 10. The sample was weighted due to the difference of absolute households within the eight randomly selected villages. There are estimated 1968 HHs in LNP. We assumed a 95.0% CI, 80.0% power, an estimated 87.0% knowledge of rabies based on previous studies and 9.0% of non-response and we obtained a minimum sample size of 174. From all villages of LNP (N=13), eight (n=8) were randomly selected and the households number, within each village were drawn by using weighting for the total households in each village.**

Comment [A8]: *With this sample size, is this representative of the national or regional areas?*

**Response: The sample size is representative of the region (LNP).**

Comment [A9]: *Describe how households and participants were selected. If the method used were standardized, selection bias will be low.*

**Response: The participants were purposively selected. The community leaders identified each of the household representatives, and the study team subsequently explained the objectives to them and asked for their consent to participate. The respondents’ willingness, oral consent to participate and availability at the time of the visit were the first eligibility criteria. The second eligibility criterion was those representatives of households aged ≥18.**

**The authors also included these criteria in the abstract as suggested by Reviewer #1 (Page 2).**

Comment [A10]: *How many persons each?*

**Response: Page 11. The information has been included in the manuscript: two doctors, eight physician’s assistant, 19 nurses, 11 caregivers and two technicians.**

Comment [A11]: *Self-administered*

**Response: Page 11. The word “…self-administered…”, was inserted in the text.**

Comment [A12]: *For easy comprehension, you may also say, “the KAP on rabies (dependent variables) were compared against HH respondents’ village, gender, and educational level, and health practitioners’ health post, years of service (independent variables).”*

**Response: The authors are grateful for the suggestion and have proceeded as recommended (Page 12).**

Comment [A13]: *Chi-square*

**Response: The correction has been made in the manuscript. Page 12.**

Comment [A14]: *Is the sex of the dog relevant to the KAP on rabies or rabies incidence?*

**Response: To answer this legitimate question, and because the authors consider that the sex of the dog really has no relevance on rabies incidence including the knowledge, attitudes and practices on the same disease, we have deleted the text from page 13.**

Comment [A15]: *(n=?; %)*

**Response: The following values were included (64.9%; 24/37), page 15.**

Comment [A16]: *Was vaccination card shown as proof?*

**Response: Some respondents showed the certificate issued by the Massingir veterinary authorities. Those who claimed to have vaccinated their dogs but did not show the certificate, their animals were subjected to blood sampling to assess the level of antibodies.**

Comment [A17]: *Showing the p value alone is okay. Not necessary to mention degrees of freedom and other values.Is this p value referring to vaccination campaigns in villages or the comparison between vaccinated and unvaccinated dogs?*

**Response: Page 16. The text has been updated to reflect the suggestions contained in the comments.**

**The *P*-value refers to the comparison between the villages.**

Comment [A18]: *Very long sentence. Split into half for easy understanding.*

**Response: Page 17. The sentence was split into half.**

Comment [A19]: *Be consistent in this style of reporting throughout the whole paper.*

**Response: Pages 17 and 18. In accordance with the above suggestion, the authors decided to standardise the format of presentation of all numerical results that appear throughout the manuscript. Preference were given to placing first the percentages and in brackets the number of members of sample (n).**

Comment [A20]: *Wrong value presentation. State the %, not the n.*

**Response: Page 19. The values presented in Fig 4 were corrected.**

Comment [A21]: *How many?*

**Response: Page 20. (50.0%; 114/228).**

Comment [A22]: *Be consistent which value to place inside the parenthesis, n or %?*

**Response: Pages 20 and 21. In accordance with the above suggestion, the authors decided to standardise the format of presentation of all numerical results that appear throughout the manuscript. Preferences were given to placing first the percentages and in brackets the number of members of sample (n).**

Comment [A23]: *How many?*

**Response: Page 20. 27 out of 59.**

Comment [A24]: *respondents*

**Response: The word “respondents” was added. Page 20.**

Comment [A25]: *their*

**Response: The word “their” was added. Page 20.**

Comment [A26]: *How many?*

**Response: Page 20. (93.0%; 212/233).**

Comment [A27]: *if*

**Page 20. Corrected and “is” was deleted and replaced by “if”.**

Comment [A28]: *In general, the respondents had good knowledge (n=44; 18.%) and practices (n=31; 13.3%) on rabies, and a positive attitude (n=203; 87.1%) toward the disease.*

**Response: Page 21 and 22. The new updated text has been added to the manuscript as suggested.**

Comment [A29]: *shows*

**Response: Page 21. Corrected.**

Comment [A30]: *respondents’ education*

**Response: Page 21. Corrected.**

Comment [A31]: *place these values beside their KAPs for easy reference*

**Response: The authors appreciate the suggestion and prompt correction was made. Page 21.**

Comment [A32]: *show values*

**Response: Page 21. The following values were added in the manuscript (18.6%, 84.9% and 12.8%, respectively).**

Comment [A33]: *Split into 2 sentences.*

**Response: Page 21. The text was split into 2 sentences.**

Comment [A34]: *Refrain from using respectively style repeatedly. It becomes confusing and difficult to understand. Place values close to the that which it describes.*

**Response: The authors thank you once again for the observation made. They were considered not only on page 21, but throughout the entire manuscript.**

Comment [A35]: *Of what?*

**Response: The text was updated. Page 22.**

Comment [A34, 38, 39]: *Remove columns*

**Response: Columns removed. Page 23 (Table 4).**

Comment [A37]: *P value*

**Response: Corrected and written in italics (Page 23, Table 4).**

Comment [A40]: *The average length of service was 4.57 years (median: 2 years; range: 1 to 30 years).*

**Response: The above sentence was added to the paragraph. Page 25.**

Comment [A41]: *Health practitioner respondents*

**Response: The title was updated. Page 25.**

Comment [A42]: *Study participants*

**Response: Page 26. The words “the personnel” were replaced by “Study participants”.**

Comment [A43]: *How many?*

**Response: 50.0% (n=21). Page 26.**

Comment [A44]: *p*

**Response: Corrected. Page 26.**

Comment [A45]: *Health practitioners’*

**Response: Corrected. Page 26.**

Comment [A46]: *(69.0%)*

**Response: Value inserted. Page 26.**

Comment [A47]: *site/severity*

**Response: Corrected. Page 26.**

Comment [A48]: *n=?*

**Response: (57.1%; n=24). Page 26.**

Comment [A49]: *Do not abbreviate unless you introduced it earlier.*

**Response: Corrected by removing the abbreviation. Page 26.**

Comment [A50]: *Same as above*

**Response: Corrected by removing the abbreviation. Page 27.**

Comment [A51]: *%*

**Response: (90.5%). Page 27.**

Comment [A52]: *while*

**Response: The word “but” was replaced by “while”. Page 27.**

Comment [A53]: *respondents*

**Response: The word “respondents” was added to the text. Page 27.**

Comment [A54]: *Interviewees with more than two years’ experience (33.3%) were more likely to know this than their less experienced colleagues (12.5%) (p=0.136)*

**Response: The sentence was corrected and updated. Page 27.**

Comment [A55]: *Only seven respondents*

**Response: The sentence was deleted because it was a repetition of the one stated in the previous paragraph (Page 26) but with different numbers.**

Comment [A56]: *How can this be attitude? Perhaps practices only for wound management.*

**Response: The figure 7 heading was corrected and updated. Fig 7. Page 28.**

Comment [A57]: *Better to place here the question asked.*

**Response: The question asked was placed and the title was updated. Page 28.**

Comment [A58]: *Place this near the groups they are describing.*

**Response: The authors proceeded according to the suggestion. Page 28.**

Comment [A59]: *Either you put the CI or the P value but be consistent throughout the paper.*

**Response: As previously mentioned, the authors have updated the format of the presentation of the results. In this case we only left the *P* value (Page 28).**

Comment [A60]: *Regarding*

**Response: The word “In respect of” were replaced by “Regarding”. Page 28.**

Comment [A61]: *(n-35; 83.3%)*

**Response: Corrected. Page 28.**

Comment [A62]: *Study participants*

**Response: The word “Study participants” were added. Page 28.**

Comment [A63]: *Split into 2 sentences.*

**Response: Page 28. The paragraph was split into 2 sentences.**

Comment [A64]: *n=?*

**Response: (n=25). Page 29.**

Comment [A65]: *route*

**Response: The word was added. Page 29.**

Comment [A66]: *n=?*

**Response: (n=9). Page 29.**

Comment [A67]: *route*

**Response: The word was added. Page 29.**

Comment [A68]: *method*

**Response: The word “route” was replaced by “method”. Page 29.**

Comment [A69]: *treatments*

**Response: The word “measures” was replaced by “treatments”. Page 29.**

Comment [A70]: *for*

**Response: The word “for” was updated to “For”. Page 29.**

Comment [A71]: *Health professionals who answered correctly about WHO recommended rabies treatment*

**Response: The sentence was updated. Page 29.**

Comment [A72]: *Remove this. It is confusing the readers. Show only those who answered correctly.*

**Response: Table 6 was corrected and only those who answered correctly are shown. Page 29.**

Comment [A73]: *n=? Show n of all % to be consistent throughout the report.*

**Response: The authors have updated the format of the presentation of the results. Page 30.**

Comment [A74]: *Answered correctly*

**Response: The words “had the best level of” was replaced by “answered correctly about”. Page 30.**

Comment [A75]: *According to location, age, gender, occupation, etc*

**Response: The sentence was updated. Page 31.**

Comment [A76, A77. A78]: *Remove*

**Response:** **χ2 and Df were removed. Page 31 (Table 7).**

Comment [A79]: *Arrange systematically*

**Response:** **Table 7 was arranged. Page 32 (Table 7).**

Comment [A80]: *Remove. Chi-square and Fisher’s are the same. The only difference is Fisher’s is used when popualtions are very small. So, it is understood.*

**Response:** **Chi-square and Fisher’s were removed. Page 32.**

Comment [A81]: *Was this proven in this study?*

**Response:** **No, it was not proven that the predominance of males dogs observed in this study was due to the fact that owners wanted to avoid the birth of undesirable puppies and the nuisance of a bitch in oestrus that attracts intact males and results in fighting. To prove this trend more demographic studies are needed. Page 33.**

Comment [A82]: *Treatment from*

**Response:** **The word was added. Page 34.**

Comment [A83]: *Not necessary to state % in discussion. They were already stated in the results section.*

**Response:** **The authors appreciate the observation. Corrected. Page 34.**

Comment [A84]: *As few as*

**Response: The words “as fewer than” was replaced by “as few as”. Page 35.**

Comment [A85]: awareof

**Response:** **The word was corrected. Page 35.**

Comment [A86]: *Not necessary to state P values in the Discussion section*

**Response:** **The authors appreciate the observation. Corrected. Page 36.**
