# Supplementary material for: Knowledge, attitudes, practices (KAP) and control of rabies among community households and health practitioners at the human-wildlife interface in Limpopo National Park, Massingir District, Mozambique
Source: PLoS Negl Trop Dis. 2022 Mar 7;16(3):e0010202. doi: 10.1371/journal.pntd.0010202 (PMC8929695; doi:10.1371/journal.pntd.0010202)
Supplement: Attachment — Submitted filename: Comments.docx [file pntd.0010202.s006.docx]

Comments:

1. Line 166-186: There was too much discussion on the calculation of the sample size but missed the two most important things readers would be interested in—the margin of error and confidence interval. Suggest to remove the explanation on the calculation. Report instead the calculated ME and 95%CI of the sample. The shorter and simpler the explanation is, the better, and is much easier to understand.
2. Line 239-240: 95% CI; *P* value <.05
3. Table 1: Suggested arrange of the variables

Age:

≤19

20-29

30-39

And so forth

Occupation:

Farmer

Non-farmer (Public servant, student, etc.)

All other variables not mentioned are okay.

4. Line 243-250: Just place the percentages. Not necessary to write down the numerators and denominators in the narrative. They are already mentioned in the Tables. Do this all throughout the paper.
5. Line 259: ...single dog (22.2%)…multiple dogs (16.5%) (*P*=.605). (No need to place 0 before .605). Follow this pattern throughout.
6. Line 280: Same as above.
7. Line 286: The frequency of dog vaccination per village and reasons for non-vaccination are shown on Table 3.
8. Line 324: Only 1.6% of respondents….
9. Line 332: A high proportion (93.0%) of respondents said they would euthanize their dog if it were rabid.
10. Line 337-338: How come you say the sample having has good knowledge and practices on rabies with only 18.9% and 13.3%? What is the basis for saying good?
11. Line 358: Knowledge, attitudes and practices on rabies of the study participants in LNP based on location, gender, education, occupation
12. Line 370: The knowledge…and incubation period…were elucidated…
13. Line 379-380: …believed they knew the symptoms…and most of the health practitioners knew the disease was fatal.
14. Line 407: …were aware of the advice…
15. Line 440: All respondents? But there is only one respondent.
16. Line 442: There were more male respondents who had good knowledge…
17. Line 461: Why emphasize on the sex of the dog when it was not part of the questionnaire? What data in your study supports this claim about male dogs? I think it is better to delete that statement if your study could not prove it objectively.
