# Supplementary material for: Knowledge, attitudes, practices (KAP) and control of rabies among community households and health practitioners at the human-wildlife interface in Limpopo National Park, Massingir District, Mozambique
Source: PLoS Negl Trop Dis. 2022 Mar 7;16(3):e0010202. doi: 10.1371/journal.pntd.0010202 (PMC8929695; doi:10.1371/journal.pntd.0010202)
Supplement: Attachment — Submitted filename: PNTD-D-20-01733 II LETTER REVIEWERS COMMENTS TRACK CHANGES.docx [file pntd.0010202.s007.docx]

**2^nd^ June 2021**

Daniel Leo Horton,

Associate Editor

PLOS Neglected Tropical Diseases

Dear Prof. Horton

Subject: **PNTD-D-20-01733**

The authors would like to thank you for your correspondence regarding the second review of our manuscript “Knowledge, attitudes, practices (KAPs) and control of rabies among community households and health practitioners at the human-wildlife interface in Limpopo National Park, Massingir District, Mozambique” by Milton Mapatse, Claude Sabeta, José Fafetine, Darrell Abernethy. Responses to the reviewers' comments and changes made to the manuscript are presented below. The reviewers’ comments are presented in italics, the author’s response in bold text. Pages numbers are based on the revised (with track changes) version of the manuscript.

**Major revisions**

**Methods**

*Reviewer #1:*

*This study assessed the knowledge, attitudes and practices towards rabies among selected households and health practitioners in Limpopo National Park, Massingir district, Mozambique. Need to improve on this objective terms like control are not captured in the abstract at least reconstruct the objective in the abstract.*

**Response: The objective has been improved both in the abstract (Page 1) and along the text on page 6.**

*The number of household + medical setting samples captured in this study are a small size, why such a small sample , clearly explain the type of sampling was it purposive or convenient sampling. And justify for the small sample size(households + medical settings) obtained in this study .*

**Response:** **For the Limpopo National Park scenario, the sample size is representative of the study area or population. In the methodology, we have improved the explanation of how the sample size calculation was done (Page 8). Purposively we selected all those Households and Health practitioners who met the requirements that allowed us to achieve our objectives. Still, it was always a challenge to include some households that met the criteria to be part of the study. For more details of the difficulties in recruiting and working in a tense political climate in Limpopo National Park villages as observed in this study, please read the section on the study limitations (Page 35) including the following papers: Milgroom J, Spierenburg M. Induced volition: Resettlement from the Limpopo National Park, Mozambique. Journal of Contemporary African Studies 2008;26(4):435-48 and Milgroom, Jessica, Cees Leeuwis, and Janice Jiggins. "Limpopo case: the role of research in conflict over natural resources; informing resettlement negotiations in Limpopo National Park, Mozambique." Knowledge in action. Wageningen Academic Publishers, Wageningen, 2011. 247-276.**

**Regarding Health professionals, as it is a remote area, human resources are limited (only two doctors and physician’s assistant for the entire district). Furthermore, the health network, not only at district, but also at national level, is still below ideal and desirable in terms of human, material and infrastructural resources (Page 9).**

*Do you think these findings can be used by the authority to implement rabies control programs in River LNP if so explain.*

**Response: Thank you for this important question. It really was not clearly reflected in the manuscript on how the findings of study could help the authorities in implementing rabies control programmes in Limpopo National Park. Therefore, we have decided to elaborate more on the subject in the conclusions and recommendations chapter (Page 34 and 35).**

*Being a national park i expected that may be we have other rabies virus transmitters in the wild that have attacked humans and animals apart from domestic dogs.*

**Response: Human-Wildlife conflict does occur in LNP, but during the period covered by this study, there were no reports or confirmed cases of wild animals attacking humans. In the supplementary table (Table S2) some interviewees claimed to have seen besides dogs, also cats, cattle, sheep and goats with signs of rabies. In the last 12 years prior to the study, only cases of people being bitten by dogs were reported.**

*I have looked through the document, the results and conclusions are clearly presented, my concern as scientist before you set out to conduct this study. 1) what was your research question or hypothesis that you want to prove right or wrong about rabies in Limpopo national park.*

**Response: The Limpopo National Park (LNP) is a unique human, domestic animal and wildlife interface region characterised by a complex epidemiological relationship involving a diversity of pathogens, including rabies virus. Compared to what is observed in the bordering and neighbouring Kruger National Park, rabies research in LNP is poor and challenging due to poor infrastructure, dispersed communities and uncertainty around local commitment. The lack of rabies information in communities in the park has prompted the following research question: What do the population and health professionals in the LNP know about rabies and how can this baseline knowledge be used to support the design and implementation of disease control strategies in the human population, domestic animals and wildlife in the region?**

*Reviewer #2:*

*1. It is difficult to read. One can hardly grasp the idea of the paper because of so many typo and clerical errors.*

**Response: Thank you for the comments. The authors have thoroughly revised the manuscript and to address typo and clerical errors, it was heavily edited by a native English speaker.**

*2. There is inconsistency in the presentation of numerical data in the narrative.*

**Response: We have standardised the presentation of numerical data throughout the manuscript by prioritising the presentation of the numerical data in percentage form.**

*3. The Tables are not of sufficient quality to be acceptable to an average reader.*

**Response: The authors have improved the presentation of the information in the Tables (Table 1, Table 2 and Table 3 and Table 6) to facilitate the understanding and interpretation of the same by readers.**

*4. There are issues in the Discussion section that are emphasized to be part of the study but are not shown in the Results section, e.g. sex of the dogs.*

**Response:** **Thank you very much for the comment. The corresponding paragraph related to sex of the dogs has been deleted (Page 27).**

**Conclusions, Summary and General Comments**

*Reviewer #1: Yes the conclusions are supported by the small set of data presented regarding the smaller number of medical professions that is fine. But i dont think the households in the LNP were limited as well. Clear explanation for this need to be presented. The authors have discussed the topic well and have shown how the data collected can be useful if certain situations are improved in the area.*

**Response: The authors are grateful for the comments left by Reviewer #1. We believe that in the study limitation section (Page 34) we have elucidated the reasons for the limited participation of households in this study.** **We hope that we have addressed and taken on board your suggestions and recommendations.**

**Minor revisions for the comments found in the manuscript**

*Comment 1.	Line 166-186: There was too much discussion on the calculation of the sample size but missed the two most important things readers would be interested in—the margin of error and confidence interval. Suggest to remove the explanation on the calculation. Report instead the calculated ME and 95%CI of the sample. The shorter and simpler the explanation is, the better, and is much easier to understand.*

**Response: We have improved the description of how the sample size was established and the margin of error and confidence interval were elucidated (Page 8).**

*Comment 2.*

*Line 239-240: 95% CI; P value <.05*

**Response: The correction has been made in the manuscript (Page 11).**

*Comment 3. Table 1: Suggested arrange of the variables*

**Response: Table 1, Table 4 have been rearranged according to your suggestion (Page 11, 12 and 18).**

*Comment 4.	Line 243-250: Just place the percentages. Not necessary to write down the numerators and denominators in the narrative. They are already mentioned in the Tables. Do this all throughout the paper.*

**Response: The authors are grateful for the suggestion and have proceeded as recommended throughout the manuscript.**

*Comment 5 .	Line 259:...single dog (22.2%)…multiple dogs (16.5%) (P=.605). (No need to place 0 before .605). Follow this pattern throughout.*

*Comment 6.	Line 280: Same as above.*

**Response: Your suggestion has been taken into account throughout the paper. We have followed the pattern recommended above.**

*Comment 7. Line 286: The frequency of dog vaccination per village and reasons for non-vaccination are shown on Table 3.*

**Response: The sentence has been updated in the text (Page 14).**

*Comment 8.	Line 324: Only 1.6% of respondents….*

**Response: The text has been corrected (Page 16).**

*Comment 9.	Line 332: A high proportion (93.0%) of respondents said they would euthanize their dog if it were rabid.*

**Response: The text has been corrected (Page 16).**

*Comment 10.	Line 337-338: How come you say the sample having has good knowledge and practices on rabies with only 18.9% and 13.3%? What is the basis for saying good?*

**Response: We have adopted a scoring system to rank the responses as such (see the topic on Data entry and analysis) (Pages 10 and 11).**

*Comment 11.	Line 358: Knowledge, attitudes and practices on rabies of the study participants in LNP based on location, gender, education, occupation*

**Response: The caption of Table 3 has been updated as suggested (Page 18).**

*Comment 12.	Line 370: The knowledge…and incubation period…were elucidated…*

**Response: The text has been edited (Page 20).**

*Comment 13.	Line 379-380: …believed they knew the symptoms…and most of the health practitioners knew the disease was fatal.*

**Response: The text was updated (Page 20).**

*Comment 14.	Line 407: …were aware of the advice…*

**Response: The text has been updated (Page 22).**

*Comment 15.	Line 440: All respondents? But there is only one respondent.*

**Response: We agreed with your comment and we have reworded the text (Page 24).**

*Comment 16.	Line 442: There were more male respondents who had good knowledge…*

**Response: We have corrected the sentence (Page 24).**

*Comment 17.	Line 461: Why emphasize on the sex of the dog when it was not part of the questionnaire? What data in your study supports this claim about male dogs? I think it is better to delete that statement if your study could not prove it objectively.*

**Response: Thank you very much for the comment. The statement has been deleted (Page 27).**
