# Supplementary material for: Knowledge, attitudes, practices (KAP) and control of rabies among community households and health practitioners at the human-wildlife interface in Limpopo National Park, Massingir District, Mozambique
Source: PLoS Negl Trop Dis. 2022 Mar 7;16(3):e0010202. doi: 10.1371/journal.pntd.0010202 (PMC8929695; doi:10.1371/journal.pntd.0010202)
Supplement: Attachment — Submitted filename: Response Reviewer 2.docx [file pntd.0010202.s009.docx]

**24^th^ August 2021**

Gino C. Matibag,

Reviewer #2

PLOS Neglected Tropical Diseases

Dear Prof. Matibag,

Subject: **PNTD-D-20-01733R2 Response to Reviewer**

On behalf of myself and the co-authors, I would like to thank you for the time you have devoted to reviewing the manuscript. In this version, we had to improve the presentation of the results, especially those that should be presented in the format of tables. As a reflection of this, the discussion has also been improved, taking your comments into account.

We hope we have met your expectations. Please see the responses below.

Kind regards

Milton Mapatse

**Major revisions**

**Methods**

*Reviewer #2:*

*Reviewer #2: The objectives are clear, methods appropriate. However, due to the smallness of the sample, there needs to be weighting of the sample, which means tweaking of the numbers. Had the sample been robust or met the minimum, a straight forward calculation of the required sample size would have been easy.*

**Response: Reviewer #2’s comment is timely. The final sample size turned out to be small. The calculations were tweaked taking into account the number of potential respondents who subsequently ended up consenting to participate in the study. In addition, the tweaks also took into account not only the minimum sample size calculated, but included the 9% of non-responses.**

**Results**

*Reviewer #2: As this is the third or fourth draft of this paper that I have reviewed, I am disappointed to say that although it is better than the earlier versions, the present quality of the presentation is not good for publication at this stage.*

*For example, some data are better presented in Tables rather than in Figures. Other data are presented in the narrative but could not be found in Tables of Figures.*

*The Tables are not uniformly well presented. The level of significance are not shown in some Tables (e.g., Table 4 and 5) where they are necessary.*

*Additional comments are in the paper.*

**Response: We would like to thank Reviewer #2 for his contributions to the improvement of all manuscript drafts submitted so far. To meet his suggestions, we have improved the presentation of the tables, i.e. their standardization and indentation. We have also inserted P values and confidence intervals where applicable. Some results summarised as narratives were edited and presented in full as tables. Some figures with results were transformed into tables.**

*Reviewer #2: The conclusion partially answered the objectives. It is lacking in explanation why HP level of KAP is lower than that of the community. Limitations are described. Relevance to public health is described, however, the paper is poorly organized and presented that the association with public health measures is weak.*

**Response: Given the scarcity of published scientific information on rabies in Mozambique in general and in Massingir in particular, we decided to proceed with this baseline study. The ultimate goal of this work is to contribute to increasing the awareness of all stakeholders about anger through the gaps and recommendations presented here. The study had two components, one that only focused on households and one that focused on health practitioners. Therefore, the issue of public health was addressed in a way that met the expectations of the academics and the Reviewer.**

**As the Reviewer #2's question is crucially important we think it makes us reflect that the specific objectives were not well clarified in the manuscript for the two distinct target populations of this study.**

**Briefly, the specific objectives of this work for the two groups were distinctively as follows. Regarding households: (i) to assess the knowledge, attitudes and practices of the households related to rabies and its control; (ii) to assess the knowledge, attitudes and practices of the households related to animal bites; (iii) to assess the general practices of the households related to veterinary care seeking behaviour.**

**As for the health practitioners they were: (i) to assess the knowledge about rabies and guidelines for its prevention; (ii) to assess the knowledge, attitudes and practice related to bite wounds and its management and finally (iii) to assess the knowledge the WHO rabies vaccination guidelines including the sites and routes. Therefore, and according to each objective, two questionnaires with distinct questions were developed, one for households and the other for health practitioners.**

*Editorial and Data Presentation Modifications/Summary and General Comments*

*Reviewer #2: There are still major revisions that need to be addressed particularly in the Discussion section.*

**Response: After organising the results according to each KAP, we improved the discussion. To do this, we paid attention to the significance of the results of this study, we sought to explain our results by comparing them with those of other studies carried out in other regions, and we also explained the reasons for such differences. We tried to bring the implication of the results presented here, explaining the impacts they may bring. And because no fieldwork is perfect, we have also brought the difficulties we encountered while conducting it.**

**Comments found in the manuscript**

*Page 3, line 46-49: Briefly explain why HH respondents have higher KAP level than health practitioners.*

**Response: We have already explained that it is difficult to compare differences in outcomes between two completely different groups. However, the justification found was incorporated into the manuscript. “…Village location and education level *(P*<.05) were found to be statistically associated with knowledge of rabies among households as compared to HPs”.**

*Page 8, line 171: Is this correct ir typo?*

**Response: It was a typo. Corrected.**

*Page 8, Lines 183-185: What is the relevance of the language used in the interviews and recording of responses?*

*If there is no significant relationship, better to rephrase this.*

**Response: The sentence was rephrased in accordance with the Reviewer's suggestion.**

*Be consistent in your writing style.*

*Page 10, lines 215-218: Is it "Poor" or "knowledege" or attitudes/practices?*

**Response: For a better consistency in the textual writing, we have made the necessary corrections according to the Reviewer's observation.**

*Page 11, Table 1. Rename to Socio-demographic characteristics pf study participants*

*(Move all variables in this column to the left. In all Tables.)*

**Response:** **We have proceeded as recommended by the Reviewer.**

*Page 11, Table 11: Place all variables in the first column. There should be only two columns in total for easy reading.*

*Mind the indents for headings and variables under each heading. Follow this in all Tables.*

**Response: We have proceeded as recommended by the Reviewer.**

*Page 11, Line 245: P value?*

**Response: We have included it.**

*Page 12, Table 2: Where is the p value column?*

**Response: After the reconfiguration of the tables, the *P* values were included.**

*Page 13, Lines 270-272: p value?*

**Response: *P* values were included in the respective paragraph.**

*Page 13, Line 274: Better to present the data in Tables than in Figures.*

**Response: The Reviewer's comment was noted with thanks and promptly complied with.**

*Page 13, Line 278: These percentages do not match those in Fig 3.*

**Response: There were shortcomings in the numbering and correct alignment of the figures with the text. Nevertheless, most of the results presented in the figures have been transformed into text or tables, as suggested by the Reviewer. Hence it is all corrected.**

*Page 13, Line 280: Where is the Figure for this?*

**Response: We have corrected it and instead of figure, it should be Table 2.**

*Page 14, Line 286 to 291: Where is the Figure for this?*

**Response: We have corrected it and it should be Table 2.**

*Page 14-15, Lines 298-315: Where is the Table for all these data?*

**Response: All the results are in the supplementary information, in Table 2.**

*Page 15, lines 315-329: To better present the results, discuss the independent variables according to each KAP.*

*For example, what independent variables showed good/bad results according to knowledge, attitude, and practices?*

*Divide each level in paragraphs so that readers will better undertand you..*

**Response:** **Thank you very much for this important comment. Reviewer #2 suggestions have been followed to the letter.**

*Page 22, line 414: What is the data for females?*

**Response:** **Thank you very much for this important comment. Reviewer #2 suggestions have been followed to the letter.**

*Page 22, line 414: What is the data for females?*

**Response: Data is presented in Table 7.**

*Page 25, line 424:There was too much discussion of studies abroad, which should have been cited in the Introduction section.*

*This section of the paper needs to discuss in great detail the findings of the survey.*

*What are the plausible explanations for such findings? If this were fully explored, it would justify the possible conclusion and recommendations that you may have.*

**Response: After organising the results according to each KAP, we improved the discussion. To do this, we paid attention to the significance of the results of this study, we sought to explain our results by comparing them with those of other studies carried out in other regions, and we also explained the reasons for such differences. We tried to bring the implication of the results presented here, explaining the impacts they may bring. And because no fieldwork is perfect, we have also brought the limitations we encountered while conducting it.**

*Page 25, line 429-432: Check grammar. Very long sentence. Consider revising.*

**Response: Revision made.**

*Page 25, lines 433-440: Most of the on-going discussion is a review of literature that should be placed in the Introduction section.*

*What is important to discuss in this section are the explanation of the results of the KAP survey.*

*Explain why health practitioners have lower level of KAP than household participants.*

*What multi-sectoral approach could do to improve the KAP in both cohorts? It is not only the government's responsibility to educate them. Other sectors need to be involved as well.*

**Response: Reviewer #2 is absolutely right. As stated above, we focused only on discussing the main results of KAP. Therefore, the appropriate changes were considered and some paragraphs with unclear or unnecessary content to be included in the discussion were either rephrased or deleted.**

*Page 31, lines 567-569: This sentence is backed up by the findings? Were there actual questions asked about how HPs gathered information about rabies?*

*If not, revise it to appear as a possible cause for the poor KAP.*

**Response: Possible causes revised as suggested by the Reviewer #2.**

*Is this recommendation the one that applies for the communities?*

*Consider discussing recommendations for HPs and communities in separate paragraphs for clarity.*

**Response: We have separated recommendations according to the Reviewer's suggestions.**
